# Population Pharmacokinetics of the Antituberculosis Agent Pretomanid

**DOI:** 10.1128/AAC.00907-19

**Published:** 2019-09-23

**Authors:** David H. Salinger, Vishak Subramoney, Daniel Everitt, Jerry R. Nedelman

**Affiliations:** a Certara, Inc., Princeton, New Jersey, USA; b TB Alliance, New York, New York, USA

**Keywords:** *Mycobacterium tuberculosis*, antibiotic resistance, multidrug resistance, tuberculosis

## Abstract

A population pharmacokinetic (PopPK) model for pretomanid was developed using data from 14 studies in the pretomanid development program: six phase 1 studies, six phase 2 studies, and two phase 3 studies. The final analysis data set contained 17,725 observations from 1,054 subjects, including healthy subjects and subjects with drug-sensitive, multidrug-resistant, or extensively drug-resistant pulmonary tuberculosis dosed pretomanid in monotherapy or combination therapy for up to 6 months. Pretomanid pharmacokinetic behavior was described by a one-compartment model that at a given dose was linear in its absorption and clearance processes but where the rate of absorption and extent of bioavailability changed with dose. Clearance and volume of distribution scaled allometrically with weight. Apparent clearance in females was 18% less than in males. Among HIV-positive subjects, absent the effect of CYP3A4-inducing antiretrovirals, apparent clearance was 6% higher. Some effects of total bilirubin and albumin were found, but the impacts on exposure were small. Bioavailability in the fasted condition was about half that in the fed condition. Relative bioavailability decreased with increasing dose in the fasted condition, but not for doses of ≤200 mg in the fed condition. HIV-positive subjects taking efavirenz and lopinavir/ritonavir had exposures that were reduced by 46 and 17%, respectively. There was little evidence for noteworthy effects of regimen partners on pretomanid. Standard diagnostics indicated that the model described the voluminous, diverse data well, so that the model could be used to generate exposure metrics for exposure/response analyses to be reported elsewhere.

## Abstract (precis)

A population pharmacokinetic (PopPK) model for pretomanid was developed using data from 14 studies in the pretomanid development program: six phase 1 studies, six phase 2 studies, and two phase 3 studies. The final analysis data set contained 17,725 observations from 1,054 subjects, including healthy subjects and subjects with drug-sensitive, multidrug-resistant, or extensively drug-resistant pulmonary tuberculosis dosed pretomanid in monotherapy or combination therapy for up to 6 months.

## TEXT

Tuberculosis (TB), caused by Mycobacterium tuberculosis, is one of the top 10 causes of death and the world’s leading cause of death from a single infectious disease. Although globally distributed, in 2017 two-thirds of cases occurred in eight countries: India, China, Indonesia, the Philippines, Pakistan, Nigeria, Bangladesh, and South Africa. Less than 10% of cases were in the WHO European Region and the WHO Region of the Americas, combined (1).

First-line treatment for TB is a combination of the four drugs rifampin, isoniazid, pyrazinamide, and ethambutol. TB caused by a strain of M. tuberculosis with no resistance to these four drugs is considered drug susceptible (DS). In 2017, approximately 558,000 people developed TB that was resistant to rifampin; 82% of these subjects were also resistant to isoniazid, a condition known as multidrug-resistant TB (MDR-TB) (1). Among the most difficult-to-treat M. tuberculosis strains are those causing extensively drug-resistant TB (XDR-TB), defined as MDR-TB with additional resistance to at least one fluoroquinolone and a second-line injectable agent (amikacin, capreomycin, or kanamycin). Among the cases of MDR-TB in 2017, 8.5% were estimated to have XDR-TB (1). Therefore, the need for innovative antituberculosis regimens is great.

Pretomanid (Pa), a new chemical entity of the nitroimidazooxazine class, is under investigation for the treatment of TB. It continues to be studied in defined regimens in various combinations with partners bedaquiline (B), clofazimine (C), moxifloxacin (M), linezolid (L), and pyrazinamide (Z). These defined regimen combinations include BPaL in Nix-TB (phase 3, completed [2]), TB-PRACTECAL (phase 2/3, ongoing [https://clinicaltrials.gov/ct2/show/record/NCT02589782]), and ZeNix (phase 3, ongoing [https://clinicaltrials.gov/ct2/show/NCT03086486]); BPaCL and BPaML in TB-PRACTECAL; and BPaMZ in NC-005 (phase 2b, completed [3]) and SimpliciTB (phase-2c, ongoing [https://clinicaltrials.gov/ct2/show/NCT03338621]). Across these studies, patients with all types of TB, including DS, monoresistant, MDR, and XDR, are included.

The objective of this work was to develop a population pharmacokinetics (PopPK) model that quantifies the behavior of pretomanid in the diversity of populations represented by the ongoing clinical studies and expected future clinical use. Characterizing the dose/response relationship of a new drug is a key challenge of drug development (4). PopPK can help to meet this challenge in several ways: identifying covariates that affect PK variability and hence treatment response, deriving PK exposure metrics from sparse data in clinical trials for use in exposure/response modeling, and simulating exposures under new dosing regimens (5).

Prior to the development of this PopPK model, the pretomanid development program had already identified several notable features of pretomanid PK including: a high-fat, high-calorie breakfast increased the area under the concentration-time curve (AUC) following a single-dose of pretomanid by 88% (6); under fasting conditions, pretomanid exposure increased less than proportionally with dose, with little additional increase after 1,000 mg (7); multiple doses of rifampin, efavirenz, and ritonavir-boosted lopinavir reduced the AUC of pretomanid by 66, 35, and 17%, respectively (8). However, these results were based primarily on studies in healthy volunteers, and they had not been combined into a comprehensive model that accounted simultaneously for other intrinsic and extrinsic factors that might affect PK. Although a PopPK model has previously been developed for pretomanid (9), it was based on two phase 2a studies in 122 DS-TB subjects where pretomanid was administered as monotherapy under fasted conditions. The present work considers 12 additional studies that include phase 2b and phase 3 studies of pretomanid administered as part of multidrug regimens to a more diverse collection of subjects, 1,054 in total, including patients with MDR-TB and XDR-TB, under fed and fasted conditions (see below and Tables S1 and S2 in the supplemental material for more details on study conduct and subject diversity). Thus, the present work provides a PopPK model more broadly useful for addressing dose/exposure/response relationships of pretomanid.

## RESULTS

### Data.

Data were pooled from six phase 1, six phase 2, and two phase 3 studies. There were 17,725 observations from 1,054 subjects. A total of 162 healthy subjects (HS) received single oral doses of pretomanid ranging from 50 to 1,500 mg, and 49 received multiple oral doses ranging from 200 to 1,000 mg once daily (q.d.) for 7 or 8 days. A total of 122 subjects with DS-TB received pretomanid alone at 50 to 1,200 mg q.d. for 14 days; and 623 subjects with DS-TB or MDR-TB received pretomanid at doses of 100 mg or 200 mg q.d. in combination with one or more of bedaquiline, clofazimine, moxifloxacin, and pyrazinamide for 8 weeks to 6 months. Finally, 98 subjects with XDR or treatment intolerant or nonresponsive (TI/NR) MDR-TB from the Nix-TB study received 200 mg pretomanid q.d. in combination with bedaquiline and linezolid.

All HS were from North America, and more than 95% of the subjects with TB were from sub-Saharan Africa. No HS were HIV^+^, but 24% of the subjects with TB were HIV^+^. Overall, 65% were male. The median (range) of age and weight were 27 (18 to 50) years and 75 (47 to 102) kg for HS and 31 (18 to 77) years and 53 (29 to 121) kg for subjects with TB.

See Tables S1 and S2 and Fig. S1 in the supplemental material for more details.

### Final model.

The pharmacokinetics of pretomanid was best characterized by a one-compartment drug disposition model with absorption lag represented by three transit compartments (Fig. S2) that at a given dose was linear in its absorption and clearance processes but where the rate of absorption and extent of bioavailability changed with dose. (Transit compartments are mathematical constructs used to generate a delayed peak in the modeled concentration-time curve, but they do not represent specific physiological compartments [10].) The model was parameterized in terms of relative bioavailability (F1), where 200 mg administered with fed status was given the reference value of 1; clearance (CL); volume of distribution (V2); mean transit time (MTT = 3/KTR, where KTR is the first-order rate constant between transit compartments); and first-order absorption (KA).

Interindividual random effects of the form exp(η) multiplied on all parameters. For F1, KA, and MTT, the η values were normally distributed; for CL and V2, Box-Cox transformations (11) were applied to normal η’s to characterize the long-tailed distributions. (See the final model code in the supplemental material for more details.) Random effects on F1, KA, and MTT were correlated.

Interoccasion random effects of the form exp(η), with η’s normally distributed and correlated, were multiplied on F1 and CL. Up to three occasions were defined for each trial, depending on trial length and sampling schedule (see Table S1 for details).

The residual error model was a mixture of an additive and a power model (12).

After an exhaustive, prespecified, search, many intrinsic factors were found to significantly (*P* < 0.001) impact model parameters. Clearance and volume of distribution scaled allometrically with weight (13). Apparent clearance in females was 18% less than in males. Among HIV-positive subjects not taking CYP3A4-inducing antiretrovirals apparent clearance was 6% higher. Some effects of total bilirubin and albumin were found, but the impacts on exposure were small.

Covariates that were considered but not retained as significant in the final model were: age, race, body mass index, metrics of renal function, AST, and ALT. More than 99% of the subjects had normal or only mildly impaired renal function by eGFR.

Regarding extrinsic factors, bioavailability in the fasted condition was about half that in the fed condition. Bioavailability decreased with increasing dose in the fasted condition, but not for doses ≤200 mg in the fed condition. HIV-positive subjects taking efavirenz and lopinavir/ritonavir had exposures that were reduced by 46 and 17%, respectively. There was little evidence for noteworthy effects of regimen partners on pretomanid.

Some study effects were considered per plan on absorption and bioavailability because phase 1 food effect studies, with a standard high-fat meal, had found approximate doubling of bioavailability under fed versus fasted conditions, but meal conditions were not strongly controlled in some phase 2 and phase 3 studies. Effects for NC-003 and NC-005 were retained. Other effects for Nix-TB, which was added in a final stage of modeling (see Materials and Methods), were found necessary *post hoc* to achieve an acceptable fit.

The code for the final model is provided in the supplemental material, and the parameters are provided in Table 1 . From these components, the covariate contributions may be reconstructed in detail. Because each of the model parameters F1, KA, MTT, CL, and V2 was allowed a flexible covariate structure, the impact of covariates on exposure is best understood from the simulation results described below.

**TABLE 1:** Final model parameter estimates^*a*^

Model parameter terms	Estimate	Confidence interval
THETA terms on fixed effects		
F1 (fixed)	1	
F1∼FASTED	0.513	0.485 to 0.541
F1∼DOSE&FASTED	−0.264	−0.302 to −0.226
F1∼FED&1,000 mg	−0.00302	−0.148 to 0.142
F1∼MOX*PZA	0.925	0.848 to 1.00
F1∼BDQ*MOX*PZA	1.25	1.04 to 1.46
F1∼EFV	1.24	1.03 to 1.45
F1∼HIV	0.789	0.721 to 0.856
F1∼TBIL (ref=5)	0.0880	0.0495 to 0.126
F1∼NIX	1.54	1.28 to 1.80
KA, h^−1^	1.38	1.25 to 1.52
KA∼FASTED	0.482	0.453 to 0.511
KA∼DOSE	−0.128	−0.158 to −0.0990
KA∼NC5	0.186	0.150 to 0.221
MTT, h	1.25	1.14 to 1.35
MTT∼FASTED	0.311	0.293 to 0.329
MTT∼DOSE&FASTED	−0.155	−0.187 to −0.123
MTT∼NC5	6.95E−07	−0.0350 to 0.0350
CL, liters/h	3.30	3.14 to 3.46
CL∼WT (fixed)	0.75	
SS CL, liters/h	0.175	0.135 to 0.214
CL∼HS	1.16	1.09 to 1.23
CL∼MOX	0.967	0.902 to 1.03
CL∼MOX*PZA	0.733	0.663 to 0.804
CL∼MDR, TI/NR MDR, or XDR	1.15	1.04 to 1.27
CL∼BDQ*MOX*PZA	1.32	1.12 to 1.51
CL∼EFV	2.17	1.89 to 2.45
CL∼LPVR	1.14	1.10 to 1.18
CL∼HIV	0.842	0.779 to 0.905
CL∼INDUC	1.35	1.24 to 1.46
CL∼FEMALE	0.837	0.808 to 0.867
CL∼ALB (ref=35)	0.200	0.0789 to 0.322
CL NIX WK ≥ 6, liters/h	0.466	0.285 to 0.646
V2, liters	90.4	85.8 to 94.9
V2∼WT (fixed)	1	
V2∼DOSE	0.111	0.0845 to 0.137
V2∼MDR or TI/NR MDR	1.44	1.24 to 1.64
V2∼XDR	1.75	1.39 to 2.11
F1 Var∼NIX	0.919	0.558 to 1.28
MTT Var∼NC3	−0.645	−1.06 to −0.226
Box-Cox V2 non-NIX	9.55	2.25 to 16.9
Box-Cox V2 NIX	26.0	−8.54 to 60.5
Box-Cox CL non-NIX	1.36	0.733 to 1.99
Box-Cox CL NIX	2.78	1.36 to 4.20
Proportional error (variance)	0.548	0.506 to 0.589
Additive error (variance)	11.5	5.84 to 17.2
Error power	0.795	0.789 to 0.800
		
OMEGA matrix terms		
F1∼dose/fasted	0.0274	0.018 to 0.0368
CL	0.0373	0.029 to 0.0455
V2	0.00892	0.0018 to 0.016
KA	0.309	0.252 to 0.366
KA-MTT	0.0649	0.00363 to 0.126
MTT	0.593	0.475 to 0.71
KA-F1	−0.0339	−0.0509 to −0.0168
MTT-F1	0.00391	−0.0184 to 0.0262
F1	0.0227	0.0136 to 0.0319
IOC F1	0.0412	0.0362 to 0.0462
IOC F1-CL	0.0101	0.00697 to 0.0132
IOC CL	0.0185	0.0155 to 0.0214

a ETA shrinkage (%): 6.6E+01, 2.5E+01, 5.8E+01, 4.2E+01, 4.5E+01, and 4.5E+01 (for dose/fasted-F1, CL, V2, KA, MTT, and F1) and 4.2E+01, 5.3E+01, 4.7E+01, 5.2E+01, 5.9E+01, and 6.2E+01 (for interoccasion F1 and CL [times three occasions]). All values are rounded to three significant digits. Units of measure are provided in column 1 where applicable. EPS shrinkage (%): 1.0E+01 and 1.0E+01 (for proportional and additive error terms).

**FIG 1:**
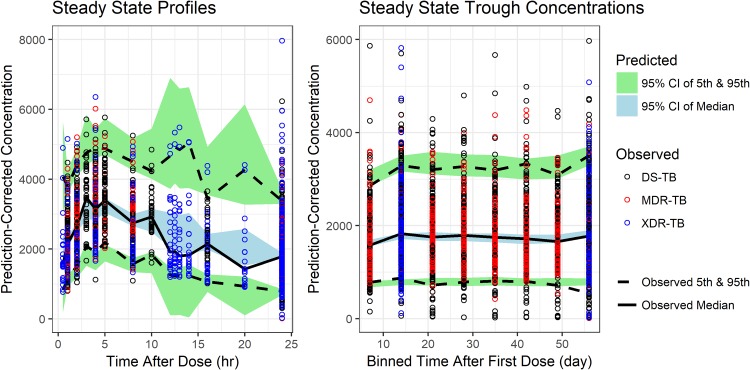
Prediction-corrected visual predictive checks of the final model. The observed and predicted medians and 5th and 95th percentiles are presented. Steady-state profiles are included for studies NC-002, NC-003, NC-005, and NiX-TB. Steady-state troughs were also included for studies NC-001 and NC-006. Additional VPCs are provided in the supplemental material.

Figure 1 shows prediction-corrected visual predictive checks (14) for the final model, indicating a satisfactory fit of the patient data. Predictive checks for other subsets of the data and standard diagnostics (15, 16) are included in the supplemental material.

### Model application.

Simulations incorporating intersubject variability were used to illustrate the effects of various covariate combinations on seven steady-state exposure criteria: the average, trough, and maximum concentrations (*C*_avg,ss_, *C*_24h,ss_, and *C*_max,ss_); the time of maximum concentration (*t*_max,ss_); the half-life (*t*_1/2_); the relative bioavailability (F1); the apparent oral clearance and volume (CL/F1 and V2/F1); and the mean total absorption time (MTT + 1/KA).

**FIG 2:**
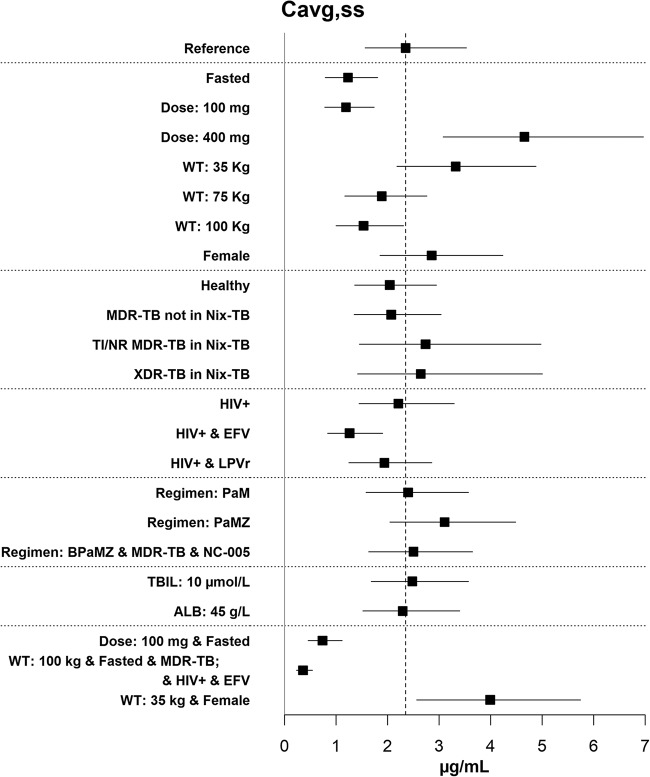
Forest plot of simulation results for *C*_avg,ss_.

The reference subject was a 55-kg, male, HIV-negative, DS-TB subject with baseline total bilirubin (TBIL) of 5 μmol/liter and albumin (ALB) of 35 g/liter administered 200 mg q.d. of pretomanid alone in a fed condition to steady state. Departures from these reference conditions that were considered may be seen in the row labels of Fig. 2, a forest plot of simulation results for *C*_avg,ss_. Forest plots and tabular summaries for this and other criteria are provided in the Fig. S3 and Table S4 in the supplemental material.

### Exposure.

The median *C*_avg_, *C*_max_, and *C*_24h_ values for a reference subject were 2.4, 3.2, and 1.6 μg/ml, respectively. Administration of pretomanid in the fasted condition reduced exposure by about half relative to the reference. The median *C*_avg_ was 22% higher in females, 13% lower in HS, and 6% lower in HIV+ subjects relative to the reference.

After the modeling results were reviewed, one group was defined by selecting covariate values that in combination would yield an extremely low exposure: fasting, HIV^+^, MDR-TB subjects weighing 100 kg, and taking efavirenz. For this group, the median *C*_avg_ was 0.36 μg/ml, and the median *C*_24h_ was 0.22 μg/ml. Otherwise, only two groups on 200 mg in the fed condition had a median *C*_avg_ of <80% of the reference value (i.e., <1.9 μg/ml): (i) weight of 100 kg, 1.5 μg/ml; and (ii) HIV^+^ and taking efavirenz, 1.3 μg/ml. After the modeling results were reviewed, one group was defined by selecting covariate values that in combination would yield an extremely high exposure: females weighing 35 kg. For this group, the median *C*_avg_ was 4.0 μg/ml, and the median *C*_max_ was 5.2 μg/ml. Otherwise, only two categories on 200 mg in the fed condition had median *C*_avg_ greater than 125% of the reference value (i.e., >3.0 μg/ml): (i) weight of 35 kg, 3.3 μg/ml; and (ii) PaMZ regimen, 3.1 μg/ml.

Although, as shown above, subjects in the PaMZ (pretomanid plus moxifloxacin plus pyrazinaminde) regimen had a median *C*_avg_ that was 130% of the reference value, in the BPaMZ (bedaquiline plus PaMZ) regimen in MDR-TB subjects in NC-005, the median *C*_avg_ was essentially the same as that of the reference value. Nix-TB-related covariates had only small effects: the media*n C*_avg_ for TI/NR MDR-TB and XDR-TB subjects was about 15% greater than the reference value. This indicates that bedaquiline and linezolid together had little impact on pretomanid exposure. In the PaM regimen, pretomanid exposure (*C*_avg_) was essentially unchanged from the reference value, and the BPa and PaZ regimens did not impact pretomanid exposure. Thus, overall, there was little evidence for noteworthy effects of regimen partners on pretomanid.

The effect of baseline values of albumin and total bilirubin were retained in the final model, but these had only small effects.

### *T*_max_.

The median *T*_max_ under reference conditions was 4.25 h (time points on quarter-hour intervals were considered). It varied only ±0.25 h for all other examined conditions, except in study NC-005, where it was 6.5 h. This was suspected to be spurious, but since there were no observations between 4 and 8 h postdose in that study it could not be readily rejected.

### Half-life.

The median half-life (*t*_1/2_) for the reference conditions was 18 h. For all other conditions, the median half-life was between 10 and 30 h.

## DISCUSSION

The benefits of modeling are said to include the simplification of complex systems and integration of diverse data (15, 17). Sometimes the pursuits of these benefits collide. This modeling of the PopPK of pretomanid may represent such a collision. At a given dose, under particular conditions and in a particular population, pretomanid PK can be described by a simple linear one-compartment model, although its late *T*_max_ requires the added complexity of transit compartments to characterize absorption. Integrating data from 14 studies with a 30-fold range of doses (50 to 1,500 mg), varying and even uncertain dosing conditions with respect to food, and various populations (healthy subjects and patients with diverse drug resistance levels of TB, with or without HIV and comedications for both diseases), led to a final model with 37 fixed-effect and 21 random-effect parameters. The model’s simplicity may be difficult to discern in Table 1. Nonetheless, in addition to the structural simplicity described above, it may be concluded that the only clinically relevant intrinsic and extrinsic associations at the recommended clinical dose of 200 mg are the approximate halving of exposure for fasted conditions relative to fed subjects and in HIV^+^ subjects taking efavirenz.

As discussed in the introduction, such a PopPK model is a step in the process of characterizing the relationship between dose and response. One subsequent use of the model is therefore to graduate and interpolate the sparsely collected PK data that went into the model in order to quantify exposure/response relationships for simultaneously collected clinical data. The model’s goodness of fit indicates its aptness for such a purpose.

Another planned application of the model is for decision making about doses in future contexts, such as in pediatrics. For such purposes, it is important to gain confidence in the model by its ability to predict new data, so-called external validation (15, 16). Such a test of the model is planned when predictions from it will be compared against data arising from the ongoing ZeNix, SimpliciTB, renal impairment, and hepatic impairment studies, as well as a planned relative bioavailability study for the pediatric formulation.

## MATERIALS AND METHODS

### Data.

Data were pooled from 14 clinical studies: CL-001 and CL-002 (7), CL-003 and CL-009 (6), CL-005 (18), CL-007 (19), CL-010 (20), DMID 10–0058 (https://clinicaltrials.gov/ct2/show/NCT01674218), NC-001 (21), NC-002 (22), NC-003 (23), NC-005 (3), STAND (https://clinicaltrials.gov/ct2/show/NCT02342886), and Nix-TB (2). Further details may be found in Table S1 in the supplemental material.

Observations with missing sample time were excluded from the analysis. Dosing times were not recorded in the four patient studies with dosing durations of ≥8 weeks, except for doses administered immediately after trough PK samples. Unrecorded dosing times were imputed based on an assumed, regular pattern of q.d. dosing or based on the recorded times of adjacent PK samples. Data below the quantification limit (BQL) were excluded from the analysis. Only 3.3% of post-first-dose observations were BQL.

Baseline values for covariates were used in data set construction except for time-dependent histories of antiretroviral comedications (on/off). For the remarkably few baseline covariate values that were missing, attempts were made to replace the missing value with a screening (or other pre-first-dose) value or to determine a reasonable replacement value. In cases where no reasonable replacement value was found, the covariate was imputed as the median (for continuous covariates) or mode (for categorical covariates) of all subjects within the same study having at least one non-BQL concentration record.

Outliers were identified by visual inspection of the raw data and were excluded. Examples of conditions that might have led to such identification were supposed trough values that were unusually high, as if the samples were collected postdose and not predose, or values supposedly around *T*_max_ that were unusually low, as if the sampling time was erroneous. Some outliers were also identified by large values of conditional weighted residuals. These were assessed visually for potential removal.

Winsorization was applied to all continuous covariates to limit the impact of extreme covariate values. For each covariate, values outside the median ± five times the standard deviation were censored to the boundary of that range. Only 21 such cases were found out of 9 continuous covariates for 1,054 subjects, which was 0.22% of the total covariate values used.

### Modeling methodology.

The approximate maximum-likelihood FOCE INTER method of NONMEM version 7.3 was used to estimate the parameters of the models. Standard methods for population PK model building and assessment (16) were employed. Model development decisions were based on objective-function values, likelihood ratio tests, parameter plausibility, standard error estimates, and diagnostic checks. More complex models were constructed by successively testing, via backward elimination, terms that contained additional parameters to be estimated. Because there were many such tests, in order to promote parsimony, the additional terms were retained if the chi-square approximation to the likelihood ratio test yielded a *P* value of <0.001. Changes in NONMEM objective function corresponding to *P* = 0.001 for one, two, three, four, five, and six parameters are 10.8, 13.8, 16.3, 18.5, 20.5, and 22.5, respectively, for the nested models.

Because of the large data set with multiple disease-state populations, regimen partners, and a variety of covariates, a staged model-building strategy was prespecified. In this, studies were added to the model in stages with study groupings selected to best inform a limited set of covariates in each stage. Model building began before data were available from the Nix-TB study; therefore, it was decided to undertake complete model development from base through final models using data from all the studies *except* Nix-TB. Then, data from Nix-TB were added, and a final post-Nix model was identified.

The following methodology description contains a mix of the prespecified modeling and key intermediate decision making, thus preserving the Results section for description of the final model.

### Pre-Nix model.

Base model development began with studies (or arms) in which pretomanid was dosed alone (studies CL-001, CL-002, CL-003, CL-005, CL-007, CL-009, and CL-010, as well as the two pretomanid-alone arms of DMID 10-0058). The goal was to select the structural model and the roles of four preselected covariates (food, dose, disease state [DS-TB and HS], and weight) in it.

A one-compartment model with three transit compartments to represent lagged absorption best represented these data. Covariates included the effect of weight (WT) on CL and V2 and the effects of fed/fasted status and dose on F1, KA, and MTT. After diagnostic assessment, further additions to the model included a two-step CL (with initial and steady-state CL), a dose-dependent V2 (possibly as a proxy for concentration-dependent binding), and a generalized power error model (12) of the form: Y = F + F^θ^ε_1_ + ε_2_, where ε_2_ ∼ N(0,σ_2_^2^) is the additive error, ε_1_ ∼ N(0,σ_1_^2^) is the proportional error, and θ is the power. (A value of 1 for θ reverts to the standard additive and proportional error model). An added parameter allowing for an effect on F1 for fed subjects dosed 1,000 mg significantly improved model fit. Since the clinical dose was expected to be around 200 mg, this step was taken to reduce impact of this odd data. A random effect on the dose effect for fed subjects also significantly improved model fit.

The next step in modeling was to examine potential influences on pretomanid PK of coadministered anti-TB agents bedaquiline, moxifloxacin, pyrazinamide, and clofazimine (BDQ, MOX, PZA, and CFZ). The analysis data set was augmented with NC-001; NC-003; the DS-TB arms of NC-002, NC-005, and NC-006; and the pretomanid plus MOX arm of DMID 10-0058. The greatest concerns with drug interactions were effects on clearance, which might impact bioavailability through first-pass effects. Therefore, possible effects of these four drugs were assessed on clearance and bioavailability.

Because of the varying and/or uncertain fed/fasted conditions in studies NC-002, NC-003, NC-005, and NC-006, study indicators were defined as covariates to allow study-specific adjustment to the food effects on F1, KA, and MTT, as well as effects on the variability of these parameters.

To assess the impact of MDR-TB on the PK of pretomanid, the MDR-TB arms of studies NC-002, NC-005, and NC-006 were added to the data set in this modeling step, and the MDR covariate was tested on CL, *V*_2_, KA, and F1. In studies NC-002 and NC-006, MDR patients received PaMZ, as did DS patients. However, in study NC-005, MDR-TB subjects received BPaMZ, whereas DS-TB patients received BPaZ. The impact of regmen BPaMZ on F1 and CL was also assessed at this step.

Next, the impacts of HIV status and antiretroviral (ARV) drugs on the PK of pretomanid were tested in the model. The effects of efavirenz (EFV) on F1 and CL and the effect of lopinavir/ritonavir (LPVR) on CL were added to the model (based on previous analysis). Other, less-prevalent, ARV drugs were planned to be grouped as inducers (INDUC) or inhibitors (INHIB) for testing on CL, but no subjects were on alternative inhibitors.

The effects of age, sex, race, and BMI, were assessed in the next modeling step. So too were baseline values of AST, ALT, total bilirubin, albumin, creatinine clearance (CL_CR_), and eGFR on CL and F1. (Studies of pretomanid in hepatically and renally impaired subjects are ongoing. In two mass-balance studies, <1% of the parent drug was excreted in the urine. Nonetheless, renal impairment can affect nonrenal aspects of drug absorption, metabolism, and distribution [24].) In addition, the effect of albumin on V2 was considered based on possible impact on protein binding; and the effects of a high dose (dose > 200 mg) on female subjects’ CL, KA, and MTT levels were tested based on noted gender differences in half-life at 1,000 mg in study CL-003 that were not seen at lower doses in study CL-009.

Covariances of interindividual random effects and interoccasion variability on F1 and CL were then assessed.

### Final model.

The final step of model building commenced with the availability and addition of the Nix-TB study data. Nix-TB introduced two new elements: (i) new disease states (XDR-TB, TI-MDR-TB, and NR-MDR-TB) and (ii) a new regimen that included linezolid (not previously examined) and bedaquiline. Some ARV drugs that were inhibitors of CYP3A4 were found, but only in two subjects contributing only three observations, so inhibiting ARV drugs were not evaluated as covariates.

The ability of the final pre-Nix model to predict the data from Nix-TB was checked by means of a visual predictive check (VPC) without re-estimation of parameters. The data were deemed not to be sufficiently well characterized by the model. Additional model building was necessary.

Model parameters were added to test the impacts of new disease states (XDR, TI-MDR, and NR-MDR) on CL and V2 and of NIX (study) as a covariate on F1, KA, and MTT (since the fed conditions in the study were uncertain) and of NIX (study) as a covariate on CL and F1 (to test the impact of the new regimen BPaL). After testing, the following were retained: the effect of either XDR-TB or NR-MDR-TB on CL, the effect of XDR-TB on V2, and the effect of study Nix-TB on F1.

This final model candidate was assessed via prediction- and simulation-based diagnostics, but found to be insufficient for prediction of the Nix-TB data in two key ways: (i) for the week 2, 8 and 16 troughs, the model predicted a steady state, but the trough concentration data diminished over time, and (ii) for the week 16 profiles, the model, by design, assumed lognormal distributions for random effects. However, the distribution of data appeared to be more skewed than what would be expected under such assumptions.

One possible explanation for the decreasing trough concentrations was the improved health status, including increased body weights, of subjects over time. Examination of the increased body weights showed that very few subjects had a greater than 10% increase in body weight by week 16, and many subjects lost weight. So, changing weight was insufficient to explain the decreasing concentrations.

Covariates were added to allow the CL (or F1) for Nix-TB study patients to increase (or decrease) at weeks 6 and 12. After testing, only the step-up in CL at week 6 was found to be significant and was retained.

To attempt to accommodate the longer-tailed distribution, Box-Cox-transformed variability was tested on CL and V2, with a separate shape parameter for Nix-TB versus other studies. These were found to provide significant improvement to the objective function (an ∼88-point improvement in objective function for four additional parameters) and to VPCs. With the addition of the week 6 step-up in CL and changes in distributions, the effect of the disease state XDR or NR-MDR on CL was no longer significantly different from the effect of MDR (including TI-MDR) on CL, and the two parameters were combined into one.

In summary, addition of the effect of disease-state subpopulation XDR-TB on V2 and study Nix-TB on F1 and the variability of F1, plus the four Box-Cox transformed variability shape parameters (for CL and V2 in Nix-TB and non-Nix-TB studies), constituted the final post-Nix model.

## Supplementary Material

Supplemental file 1
