# Supplementary material for: Population Pharmacokinetics of the Antituberculosis Agent Pretomanid
Source: Antimicrob Agents Chemother. 2019 Sep 23;63(10):e00907-19. doi: 10.1128/AAC.00907-19 (PMC6761531; doi:10.1128/AAC.00907-19)
Supplement: Supplemental file 1 [file AAC.00907-19-s0001.pdf]

Population Pharmacokinetics of Pretomanid – Supplemental Materials

David H. Salinger and Vishak Subramoney

Certara Inc., Princeton, NJ; under contract with the Bill and Melinda Gates Foundation

Daniel Everitt and Jerry R. Nedelman

TB Alliance

DATA

Table S1 Overview of studies/data included in Analysis data set

- **CL-001** (phase 1, single-ascending-dose study in healthy subjects)
  - **Country:** United States
  - **Treatments:** Pretomanid 50 mg (n=4), 250 mg (n=6), 500 mg (n=6), 750 mg (n=6), 1000 mg (n=6), 1250 mg (n=6), 1500 mg (n=6), placebo
  - **Dosing:** Single dose, fasted
  - **PK:** 0, 0.5, 1, 2, 3, 4, 5, 6, 7, 8, 12, 16, 20, 24, 30, and 36 hours postdose, as well as 7 days postdose
  - **Occasions:** All 1
  - **Exclusions:** Subjects assigned to placebo
- **CL-002** (phase 1, multiple-ascending-dose study in healthy subjects)
  - **Country:** United States
  - **Treatments:** Pretomanid 200 mg (n=6), 600 mg (n=6), 1000 mg (n=6), placebo
  - **Dosing:** QD days 1 – 7, fasted
  - **PK:** predose each day during the treatment period; 1, 2, 3, 4, 5, 6, 7, 8, 12, and 16 hours after dosing on Days 1 and 7; 24, 30, and 36 hours after Day 7 dosing (i.e., during Day 8); daily during washout on Days 9-13, at the time daily dosing would have otherwise occurred; and during Check-out on Day 14
  - **Occasions:** 1: Days 1 – 6;   2: Days 7 – 14
  - **Exclusions:** 1) Subjects assigned to placebo. 2) Observations after Day 5 in the 1000 mg dose group because pretomanid was discontinued after the Day 5 morning dose due to increased creatinine.
- **CL-003** (phase 1, single-dose, crossover, food-effect study in healthy subjects)
  - **Country:** United States
  - **Treatments:** Pretomanid 1000 mg fed and fasted (n=16, crossover)
  - **Dosing:** Single doses on days 1 and 9

    - **PK:** 0, 0.5, 1, 2, 3, 4, 5, 6, 7, 8, 12, 16, 24, 30, 36, 48, 72, 96, 120, 144, and 168 hours after each dose
    - **Occasions:** 1: Days 1 – 8;   2: Days 9 – 16

- **CL-005** (phase 1, multiple-dose study of the effect of pretomanid on renal function in healthy subjects)
    - **Country:** United States
    - **Treatments:** Pretomanid 800 mg (n=21, cohorts 1&2), 1000 mg (n=10, cohort 3), placebo
    - **Dosing:** QD days 1 – 8, fasted
    - **PK:** Plasma samples for PK analysis were collected before dosing each day during the treatment period (i.e., days 1 through 8) on the following schedule: 1, 2, 3, 4, 5, 6, 7, 8, 12, and 16 h after dosing on days 1 and 8; 24, 30, and 36 h after the day 8 dosing (i.e., during day 9); approximately 48, 72, and 96 h after the day 8 dosing (i.e., on days 10, 11, and 12); and during the follow-up visit on day 15 or on early withdrawal from the study. Additionally, for subjects in all cohorts except cohort 1, blood samples for PK were drawn on day 5 at 1, 2, 3, 4, 5, 6, 7, 8, 12, and 16 h after dosing.
    - **Occasions:** 1: Days 1 – 7; 2: Days 8 – 15
    - **Exclusions:** Subjects assigned to placebo

- **CL-007** (phase 2a, 14-day early bactericidal activity (EBA) study in subjects with newly diagnosed, uncomplicated, smear-positive TB)
    - **Country:** South Africa
    - **Treatments:** Pretomanid 200 mg (n=15), 600 mg (n=15), 1000 mg (n=16), 1200 mg (n=15); Control HRZE
    - **Dosing:** QD days 1 – 14, fasted
    - **PK:** 0, 0 .5, 7,2,3,4,5,6, 7,8, 12, and 16 hours after dosing on Days 1, 8, and 14; predose each day during the rest of the treatment period (i.e., Days 2 through 7 and 9 through 13); 24 and 30 hours after Day 14 dosing (i.e., during Day 15); and during the Follow-up visit on (14 days +/- 1 day after Day 14 dosing), or upon early withdrawal
    - **Occasions:** 1: Days 1 – 7; 2: Days 8 – 13; 3: Days 14 – 29
    - **Exclusions:** Control arm

- **CL-009** (phase 1, single-dose, crossover, food-effect study in healthy subjects)
  - **Country:** United States
  - **Treatments:** Pretomanid 50 mg (n=16), 200 mg (n=16)
  - **Dosing:** Single doses on days 1 and 8
  - **PK:** predose, 0.5, 1, 2, 3, 4, 6, 8, 12, 16, 24, 36, 48, 72, and 96 hours after dosing on Days 1 and 8
  - **Occasions:** 1: Days 1 – 7; 2: Days 8 – 14
- **CL-010** (phase 2a, 14-day EBA study in subjects with newly diagnosed, uncomplicated, smear-positive TB)
  - **Country:** South Africa
  - **Treatments:** Pretomanid 50 mg (n=15), 100 mg (n=15), 150 mg (n=15), 200 mg (n=16); Control HRZE
  - **Dosing:** QD days 1 – 14, fasted
  - **PK:** 0, 0.5, 1, 2, 3, 4, 5, 6, 7, 8, 12, 16 hours post dose on Days 1 and 14; predose each day during the rest of the treatment period (i.e., Days 2 through 13); 24 and 30 hours after the Day 14 dose (on Day 15); at Follow-up 14 ± 1 days after Day 14
  - **Occasions:** 1: Day 1 – 7; 2: Days 8 – 13; 3: Days 14 – 29
  - **Exclusions:** Control arm
- **CL-012 (DMID 10-0058)** (phase 1, Thorough QT Study, crossover in healthy subjects)
  - **Country:** United States
  - **Treatments:** Pretomanid 400 mg (n=74), 1000 mg (n=71), 400 mg plus moxifloxacin 400 mg (n=73), placebo, moxifloxacin
  - **Dosing:** Single doses, fasted, days 1, 8, 15, 22, 29
  - **PK:** 0 h (predose) and at 0.25, 1, 2, 3, 4, 5, 6, 7, 8, 10, 12, 24, 48, 72 (±4) and 96 (±4) hours post-dose
  - **Occasions:** 1: Pretomanid 400 mg; 2: Pretomanid 1000 mg; 3: Pretomanid 400 mg plus moxifloxacin
  - **Exclusions:** Placebo and moxifloxacin-alone periods

- **NC-001** (phase 2a, 14-Day EBA study in subjects with newly diagnosed, uncomplicated, smear-positive TB)

  - **Country:** South Africa

  - **Treatments:** Various combinations of bedaquiline (B), pretomanid (Pa), moxifloxacin (M), and pyrazinamide (Z), plus HRZE control.
    The B dosing scheme was 700 mg on Day 1, 500 mg on Day 2, 400 mg on Days 3 – 14. The Pa dose was 200 mg.
    The M dose was 400 mg.
    The Z dose was by weight (≤55 kg: 1000 mg; >55 kg – 75 kg: 1500 mg; >75 kg: 2000 mg).
    Included arms were: BPa (n=15), PaZ (n=15), PaMZ (n=15)

  - **Dosing:** QD days 1 – 14.
    PaZ and PaMZ arms fasted.
    BPa arm: B fed (within 30 minutes after breakfast), Pa fasted (4 hours before breakfast); Pa was dosed fasted; fasting continued four hours; then breakfast was eaten; then B was dosed within 30 minutes after breakfast.

  - **PK:**
    PaZ and PaMZ groups: Days 1 and 8: 0, 1, 2, 3, 4, 6, 8, 12, and 24 hours post dose; Day 14: 0, 1, 2, 5, 8, and 24 hours post dose
    BPa group: Day 1 & 8: 0, 4, 8 hours post dose; Day 14: 0, 1, 2, 5, 8, 24 hours post dose

  - **Occasions:** 1: Days 1-7; 2: Day 8-13; 3: Days 14-15

  - **Exclusions:** Arms without Pa (B, BZ, HRZE)

- **NC-002** (phase 2a, 8-week sputum-culture-conversion study with a 14-day-inpatient EBA sub-study, in subjects with DS-TB or MDR-TB

  - **Countries:** South Africa, Tanzania, Brazil

  - **Treatments:**
    DS1: Pa 100 mg + M 400 mg + Z 1500 mg (n=60, 14 sub-study)
    DS2: Pa 200 mg + M 400 mg + Z 1500 mg (n= 58, 12 sub-study)
    DS3: HRZE control
    MDR: Pa 200 mg + M 400 mg + Z 1500 mg (n= 26, 16 sub-study)

  - **Dosing:** QD, “at least 2 hours after a meal (preferably the morning meal)”. The exposures appeared consistent with the fed condition, and preliminary modeling also found consistency with fed conditions, so the fed condition was initially assumed here for modeling purposes.

  - **PK:** Weekly sampling was done pre-dose, within 2 hours prior to dosing Day 8 through Day 57. For subjects participating in the EBA sub-study,

additional PK samples were drawn pre-dose and 1, 2, 5, 8, and 24 hours post dose on Day 14.

  - **Occasions:** 1: Days 1 – 13; 2: Days 14 – 57.
  - **Exclusions:** Control arm

- **NC-003** (phase 2a, 14-Day EBA study in subjects with newly diagnosed, smear-positive TB)
  - **Country:** South Africa
  - **Treatment:** Various combinations of B, Pa, clofazimine (C), and Z, and HRZE control.
    The B dosing scheme was 400 mg on Day 1, 300 mg on Day 2, 200 mg on Days 3 – 14. The Pa dose was 200 mg.
    The C dosing scheme was 300 mg on Days 1 – 3, 100 mg on Days 4 – 14.
    The Z dose was 1500 mg.
    Included arms were: BPaCZ (n=14), BPaZ (n=14), BPaC (n=15)
  - **Dosing:** QD, fed
  - **PK:** Days 1, 2, 3, 8: 0, 5, 10 hours; Day 14: 0, 1, 2, 3, 4, 5, 10, and 16 post dose; Day 15
  - **Occasions:** 1: Days 1 – 8; 2: Days 9 – 15
  - **Exclusions:** Arms without Pa (BCZ, C, Z, HRZE). Visit #19, nominally Day 28, where all observations should be BQL

- **NC-005** (phase 2b, 8-week sputum-culture-conversion study in subjects with DS-TB and MDR-TB. DS-TB subjects were randomized to one of two BPaZ arms or control (HRZE); MDR-TB subjects received BPaMZ.)
  - **Countries:** South Africa, Tanzania, Uganda
  - **Treatment:**
    DS1: B was dosed as labeled: 400 mg QD for 2 weeks followed by 200 mg TIW.
    DS2 and MDR: B was dosed 200 mg QD.
    The Pa dose was 200 mg.
    The M dose was 400 mg.
    The Z dose was 1500 mg.
    Included arms were: DS1: BPaZ (n=59 incl 14 PK sub-study); DS2: BPaZ (n=59 incl 18 PK sub-study); DS3: HRZE; MDR: BPaMZ (n=60 incl 13 PK sub-study)

- **Dosing:** QD. This was an outpatient study, and the fed vs fasted condition at dose administration was not fully controlled. Subjects were directed to take medication QD “preferably around breakfast time” and “should take IMP with a meal”.

- **PK:** For all patients, pre-dose samples were collected on Days 1, 4, 8, 15, 22, 29, 36, 43, 50 and during site visits on days 57 and 70. Fifteen subjects in each arm participated in a PK sub-study where on Days 14 and 56 samples were collected at 0, 1, 2, 4, 8, and 24 hours post dose.

- **Occasions:** 1: Days 1 – 13; 2: Days 14 – 55; 3: Days 56 – 70.

- **Exclusions:** Arm without Pa (HRZE)

* **NC-006** (phase 3 trial of PaMZ after 4 and 6 months in subjects with DS-TB and after 6 months in subjects with MDR-TB)

  - **Countries:** Georgia, Kenya, Malaysia, Philippines, South Africa, Thailand, Uganda, Ukraine, Tanzania

  - **Treatments:**
    DS1: 4 months Pa(100 mg)MZ (n=65)
    DS2: 4 months Pa(200 mg)MZ (n=71)
    DS3: 6 months Pa(200 mg)MZ (n=67)
    DS4: HRZE
    MDR: 6 months Pa(200 mg)MZ (n=13)

  - **Dosing:** QD. This was an outpatient study, and the fed vs fasted condition at dose administration was not fully controlled. The protocol did not specify the timing of dosing relative to a meal.

  - **PK:** Pre-dose weeks 2 and 8.

  - **Occasions:** 1 for weeks 1 and 2; 2 for weeks >2.

  - **Exclusions:** Control arm

* **Nix-TB** (phase 3, safety and efficacy study of the BPaL regimen for 6 months in subjects with XDR-TB)

  - **Country:** South Africa

  - **Treatment:** B labeled + Pa 200 mg + L 1200 mg/day, either BID or QD (n=98 included here; sub-study n=22)

  - **Dosing:** QD. This was an outpatient study, and the fed vs fasted condition at dose administration was not fully controlled. However, the protocol did

specify that subjects “should take IMP with a meal (generally allow the Subjects a window of 30 minutes before to 30 minutes after a meal)”.

- **PK:** All subjects: Pre-dose weeks 2, 8, 16. Sub-study: pre-dose, 0.5, 1, 2, 4, 8 12, 12.5, 13, 14, 16, 20, and 24 hours post dose at week 16.

- **Occasions:** 1, 2, and 3 for weeks 2, 8, and 16, respectively.

Table S2 Summary of covariates in the final analysis data set

Covariate (units)	Measure	HS	DS	Non-Nix MDR	Nix XDR and MDR	All	All TB
**AGE (years)**	N	211	654	93	98	1056	845
	mn.sd	29.1 ± 8.57	33.2 ± 11	33.6 ± 11.4	35.8 ± 9.84	32.6 ± 10.7	33.5 ± 11
	median	27	31	32	34	30	31
	range	(18 - 50)	(18 - 77)	(18 - 69)	(18 - 60)	(18 - 77)	(18 - 77)
**ALB (g/L)**	N	211	654	93	98	1056	845
	mn.sd	44.8 ± 3.18	35 ± 5.01	35.3 ± 5.04	36.7 ± 4.75	37.2 ± 6.04	35.3 ± 5.01
	median	45	35.5	35.5	37	37	35.8
	range	(37 - 54)	(18.9 - 47.5)	(22.5 - 46)	(22 - 50)	(18.9 - 54)	(18.9 - 50)
**ALT (U/L)**	N	211	654	93	98	1056	845
	mn.sd	20.7 ± 9.82	25.1 ± 17.6	28.6 ± 21.5	28.4 ± 21.8	24.9 ± 17.4	25.9 ± 18.6
	median	18	20.4	21.7	21	20	20.5
	range	(8 - 66)	(3 - 111)	(7 - 111)	(7 - 111)	(3 - 111)	(3 - 111)
**AST (U/L)**	N	211	654	93	98	1056	845
	mn.sd	22 ± 5.53	26.9 ± 14	29.4 ± 16.2	35.6 ± 20.2	26.9 ± 14.2	28.2 ± 15.3
	median	21	23	25	29	23	24
	range	(11 - 55)	(7.28 - 93.2)	(11 - 93.2)	(9 - 93.2)	(7.28 - 93.2)	(7.28 - 93.2)
**BMI (kg/m^2)**	N	211	654	93	98	1056	845
	mn.sd	24.6 ± 2.92	19.7 ± 3.44	19.5 ± 3.61	20.7 ± 4.99	20.7 ± 4.03	19.8 ± 3.68
	median	24.5	19	18.9	19.8	19.8	19.1
	range	(17.1 - 30)	(13.4 - 39.6)	(12.2 - 32.4)	(12.4 - 39.6)	(12.2 - 39.6)	(12.2 - 39.6)
**CRCL (mL/min)**	N	211	654	93	98	1056	845
	mn.sd	121 ± 26.3	116 ± 28.6	119 ± 31.4	112 ± 36	117 ± 29.2	116 ± 29.9
	median	117	114	113	108	114	113
	range	(74.9 - 214)	(36.5 - 260)	(61 - 260)	(41.4 - 260)	(36.5 - 260)	(36.5 - 260)

Covariate (units)	Measure	HS	DS	Non-Nix MDR	Nix XDR and MDR	All	All TB
**EGFR (mL/min/ 1.73m^2)**	N	211	654	93	98	1056	845
	mn.sd	107 ± 16.5	134 ± 18.1	139 ± 19.8	128 ± 21.1	129 ± 21.2	134 ± 18.8
	median	107	135	139	128	129	135
	range	(66.4 - 153)	(59.2 - 190)	(87.1 - 199)	(67.1 - 174)	(59.2 - 199)	(59.2 - 199)
**TBIL (μmol/L)**	N	211	654	93	98	1056	845
	mn.sd	10.9 ± 5.2	5.75 ± 2.76	4.94 ± 2.66	5.14 ± 2.81	6.66 ± 4.01	5.59 ± 2.77
	median	10.3	5	4.1	5	5.5	5
	range	(3.42 - 27.4)	(2 - 26)	(1.8 - 14.5)	(2 - 14)	(1.8 - 27.4)	(1.8 - 26)
**WT (kg)**	N	211	654	93	98	1056	845
	mn.sd	73.9 ± 11.3	54.7 ± 9.8	53.6 ± 10.1	57.6 ± 15.2	58.7 ± 13.2	54.9 ± 10.6
	median	75.3	53.2	53	56	56	53.2
	range	(47.2 - 102)	(31.8 - 121)	(35 - 81.6)	(29 - 112)	(29 - 121)	(29 - 121)
**FED[a]**	FASTED	187 (88.6%)	167 (25.5%)	0 (0%)	0 (0%)	354 (33.5%)	167 (19.8%)
	FED	24 (11.4%)	487 (74.5%)	93 (100%)	98 (100%)	702 (66.5%)	678 (80.2%)
**SEX**	FEMALE	78 (37%)	213 (32.6%)	32 (34.4%)	47 (48%)	370 (35%)	292 (34.6%)
	MALE	133 (63%)	441 (67.4%)	61 (65.6%)	51 (52%)	686 (65%)	553 (65.4%)
**HIV**	HIV-Positive	0 (0%)	111 (17%)	38 (40.9%)	53 (54.1%)	202 (19.1%)	202 (23.9%)
	HIV-Negative	211 (100%)	543 (83%)	55 (59.1%)	45 (45.9%)	854 (80.9%)	643 (76.1%)
**RACE**	Black	55 (26.1%)	414 (63.3%)	71 (76.3%)	77 (78.6%)	617 (58.4%)	562 (66.5%)
	CAU	143 (67.8%)	10 (1.5%)	2 (2.2%)	1 (1%)	156 (14.8%)	13 (1.5%)
	OTR	13 (6.2%)	230 (35.2%)	20 (21.5%)	20 (20.4%)	283 (26.8%)	270 (32%)

[a]Fed/Fasted status was not well controlled for studies NC-002, NC-003, NC-005, NC-006, and Nix-TB. Subjects were assumed Fed and 'study' effects were tested.
The summary statistics are based upon subjects' Baseline values (if available; or "Screening" values).
Note: Covariates have been Winsorized and missing values imputed as described in the text.

Figure S1 Plot of continuous covariates in the analysis dataset by study number

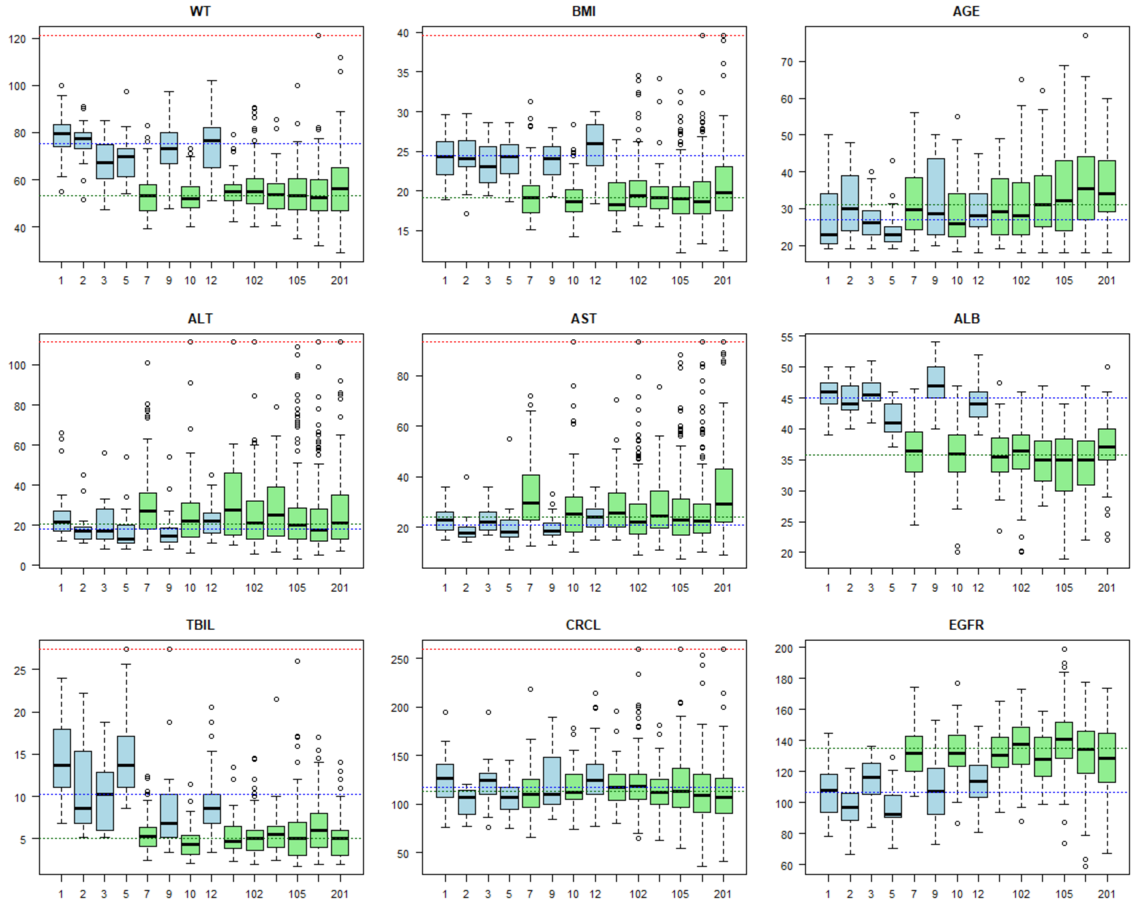

Covariates have been Winsorized and missing values imputed as described in the text. Blue boxes are studies of healthy subjects, green boxes are studies of TB subjects, blue dotted line is median of healthy subjects, green dotted line is median of TB subjects, Red dotted line represents Winsorization bound (for cases where it was utilized).
Study numbers 1 – 10 are Studies CL-001 – CL-010. Study 12 is DMID 10-0058. Study numbers 101 – 106 are Studies NC-001 – NC-006. Study number 201 is the Nix-TB study.

MODEL

Figure S2 Structural form of the population pharmacokinetic model

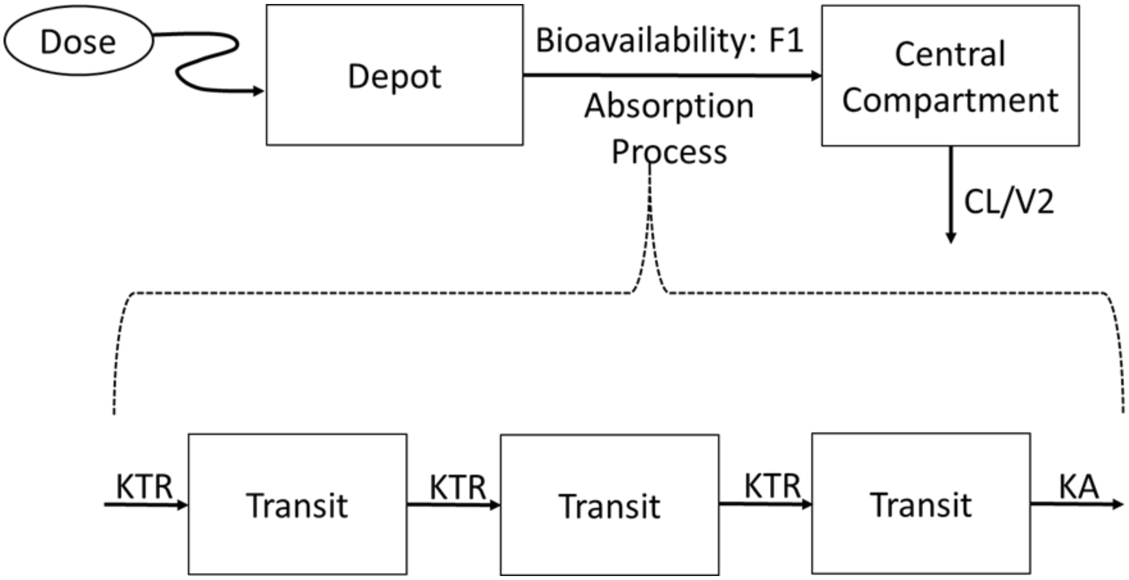

Table S3 Final Model Control File

```
$PROBLEM    PA-824
$INPUT C DROP STUDYNO DROP ID DOSE COHORT DROP STDAY STWEEK NTIME TIME
NTAD ATAD DV AMT BQL CMT EVID MDV FASTED WT BMI AGE FEMALE CAU OTR CRCL
EGFR ALT AST ALB TBIL DS MDR HIV NC2 NC3 NC5 NC6 NIX BDQ CFZ MOX PZA EFV LPVR
DROP INDUC OCC1 OCC2 OCC3 RECNO NIXPT
$DATA  PA824Full.NixPK.2018.10.11.csv IGNORE=C

$SUBROUTINE ADVAN7 TRANS1
$MODEL      COMP(TRANS1,DEFDOSE) ; 1
                      COMP(CENTRAL,DEFOBS) ; 2
                      COMP(TRANS2) ; 3
                      COMP(TRANS3) ; 4
                      COMP(ABS)       ; 5

;---PK-----------------------------------------
$PK
HV = 1 -DS -MDR           ; Healthy Volunteer Indicator
;;;; NOTE:  NIXPT = 1, 2, 3 for (MDR intolerant, MDR nonresponsive, XDR)
MDRIT=0
MDRNR=0
MDRXDR=0
IF(NIXPT.EQ.1) MDRIT=1
IF(NIXPT.EQ.2) MDRNR=1
IF(NIXPT.EQ.3) MDRXDR=1

SSD = 0
IF(STUDYNO.EQ.2.AND.STDAY.GE.5) SSD = 1
IF(STUDYNO.EQ.5.AND.STDAY.GE.5) SSD = 1
IF(STUDYNO.EQ.7.AND.STDAY.GE.5) SSD = 1
IF(STUDYNO.EQ.10.AND.STDAY.GE.5) SSD = 1
IF(STUDYNO.GT.100.AND.STDAY.GE.5) SSD = 1

SSWK6=0
IF(STUDYNO.EQ.201.AND.STWEEK.GE.6) SSWK6 = 1

DOSEIX = 0   ;indicator for 1000 mg fasted
IF(FASTED.EQ.0.AND.DOSE.EQ.1000) DOSEIX=1


TVLF1 = FASTED*LOG(THETA(4)) + ( DOSEIX*THETA(14) +FASTED*(THETA(5)
+ETA(1)) )*LOG(DOSE/200) + log(THETA(16))*MOX*PZA +log(THETA(22))*BDQ*MOX*PZA +
log(THETA(26))*EFV +log(THETA(29))*HIV +THETA(33)*LOG(TBIL/5) +log(THETA(35))*NIX

TVLKA = LOG(THETA(1)) + FASTED*LOG(THETA(6)) + THETA(7)*LOG(DOSE/200)
+log(THETA(19))*NC5

TVLCL = LOG(THETA(2)+SSD*THETA(11) +SSWK6*THETA(38)) + 0.75*LOG(WT/55) +
LOG(THETA(8))*HV  +log(THETA(17))*MOX +log(THETA(18))*MOX*PZA
+log(THETA(23))*(MDR-NIX+MDRIT+MDRNR+MDRXDR) +log(THETA(24))*BDQ*MOX*PZA +
log(THETA(27))*EFV +log(THETA(28))*LPVR +log(THETA(30))*HIV +log(THETA(31))*INDUC
+log(THETA(32))*FEMALE  +THETA(34)*LOG(ALB/35)

TVLV2 = LOG(THETA(3)) + 1*LOG(WT/55) + THETA(13)*LOG(DOSE/200)
+log(THETA(25))*(MDR-NIX+MDRIT+MDRNR) +MDRXDR*LOG(THETA(36))
```

```
TVMTT = LOG(THETA(9)) +FASTED*LOG(THETA(12)) + FASTED*THETA(15)*LOG(DOSE/200) +
LOG(THETA(20))*NC5

IOVF1 = OCC1*ETA(7) + OCC2*ETA(9) + OCC3*ETA(11)
IOVCL = OCC1*ETA(8) + OCC2*ETA(10) + OCC3*ETA(12)

F1V = EXP(THETA(37)*NIX)

F1 = EXP( TVLF1 + F1V*ETA(6) + IOVF1)
KA = EXP( TVLKA + ETA(4))

;;BOX-COX-transform V
BXPAR = THETA(39)*(1-NIX) + THETA(40)*NIX ;;value >0 or <0 (but fails at 0)
PHI = EXP(ETA(3))
ETATR = (PHI**BXPAR -1)/BXPAR
V = EXP( TVLV2 + ETATR)

;;BOX-COX-transform CL
BXPAR2 = THETA(41)*(1-NIX) + THETA(42)*NIX;;value >0 or <0 (but fails at 0)
PHI2 = EXP(ETA(2))
ETATR2 = (PHI2**BXPAR2 -1)/BXPAR2
CL = EXP( TVLCL + ETATR2 + IOVCL)

MTTV3 = EXP(THETA(21)*NC3)
MTT = EXP( TVMTT + MTTV3*ETA(5) )
KTR = 3/MTT

KEL = CL/V

S2 = V
K13 = KTR
K34 = KTR
K45 = KTR
K52 = KA
K20 = KEL

$ERROR (ONLY OBSERVATIONS)
IPRED =  A(2)/V

W = SQRT(IPRED**( 2*THETA(17) )*SIGMA(1,1)**2 + SIGMA(2,2)**2)

IWRES = (DV-IPRED)/W

Y = IPRED + EPS(1) *(IPRED+.0001)**THETA(10)  + EPS(2)            ; .01 to safeguard against
0^pow with pow<1

$THETA
1.7       ; 1 KA
3.2       ; 2 CL
94        ; 3 V
.53       ; 4 F1~FASTED
-0.23     ; 5 F1~DOSE &FASTED
0.43      ; 6 KA~FASTED
-.18      ; 7 KA~DOSE
1.24      ; 8 CL~HV
1.8       ; 9 MTT
```

```
(-1,.85,2); 10 ErrPower
.17        ; 11 CL.SS
.3         ; 12 MTT~FASTED
.11        ; 13 V~DOSE
.21        ; 14 F1~FED &1000mg
-.17       ; 15 MTT~DOSE&FASTED
0.8        ; 16 F~MOX*PZA
0.95       ; 17 CL~MOX
0.67       ; 18 CL~MOX*PZA
0.15       ; 19 KA~NC5
0.000002  ; 20 MTT~NC5
-.88       ; 21 MTTV3
1.1        ; 22 F1~BDQ*MOX*PZA
1.1        ; 23 CL~MDR-NIX+MDRIT  +MDRNR+MDRXDR
1.1        ; 24 CL~BDQ*MOX*PZA
1.1        ; 25 V2~MDR-NIX+MDRIT+MDRNR
0.88       ; 26 F1~EFV
1.35       ; 27 CL~EFV
1.20       ; 28 CL~LPVR
1.1        ; 29 F1~HIV
1.1        ; 30 CL~HIV
1.1        ; 31 CL~INDUC
1.1        ; 32 CL~FEMALE
0.1        ; 33 F1~TBIL (ref=5)
0.1        ; 34 CL~ALB (ref=35)
1.1        ; 35 F1~NIX
1.1        ; 36 V2~MDRXDR
-.2        ; 37 F1VNIX
.17        ; 38 CL.NIX.WK6
.55        ; 39 Box-Cox V2 non-NIX
1.1        ; 40 Box-Cox V2 NIX
.55        ; 41 Box-Cox CL non-NIX
1.1        ; 42 Box-Cox CL NIX

$OMEGA
.04        ; 1 F1~Dose/Fasted
.05        ; 2 CL
.05        ; 3 V2

$OMEGA BLOCK(3)
.3         ; 4 KA
.03                  ; KA-MTT
           .5        ; 5 MTT
.01                            ; KA-F1
           .01                 ; MTT-F1
                     .04       ; 6 F1

$OMEGA BLOCK(2)
0.1        ; IOC.F1
0.1        ; IOC.F1.CL
0.2        ; IOC.CL
$OMEGA BLOCK(2) SAME
$OMEGA BLOCK(2) SAME

$SIGMA
0.32       ; PropErr
```

```
18	; AddErr

$ESTIMATION  METHOD=COND INTERACTION PRINT=1 MAXEVAL=59999  NSIG=3 SIGL=9
NOABORT MSFO=7201a.MSF
$COV PRINT=E UNCONDITIONAL MATRIX=S

$TABLE ID STUDYNO DOSE COHORT STDAY NTIME TIME NTAD ATAD DV AMT BQL CMT
EVID MDV FASTED WT BMI AGE FEMALE CAU OTR CRCL EGFR ALT AST ALB TBIL DS MDR
NIX BDQ CFZ MOX PZA EFV LPVR INDUC RECNO IPRED PRED RES WRES IWRES CWRES
ETA7 ETA8 ETA9 ETA10 ETA11 ETA12 OCC1 OCC2 OCC3 F1 KA CL V MTT NOPRINT
ONEHEADER FORMAT=s1PE20.11 FILE=PA7201a.tab

$TABLE ID STUDYNO DOSE COHORT WT DS MDR MDRIT MDRNR MDRXDR FASTED AGE
FEMALE CAU OTR CRCL EGFR ALT AST ALB TBIL NIX BDQ CFZ MOX PZA EFV LPVR INDUC
ETA1 ETA2 ETA3 ETA4 ETA5 ETA6 FIRSTONLY NOPRINT ONEHEADER FORMAT=s1PE20.11
FILE=PA7201apar.tab
```

MODEL APPLICATION

Figure S3 Forest plot of simulation results

$C_{24h,ss}$, $C_{max,ss}$, $t_{max,ss}$, and steady state $t_{½}$, CL/F1, V2/F1, F1 and MTT +1/KA

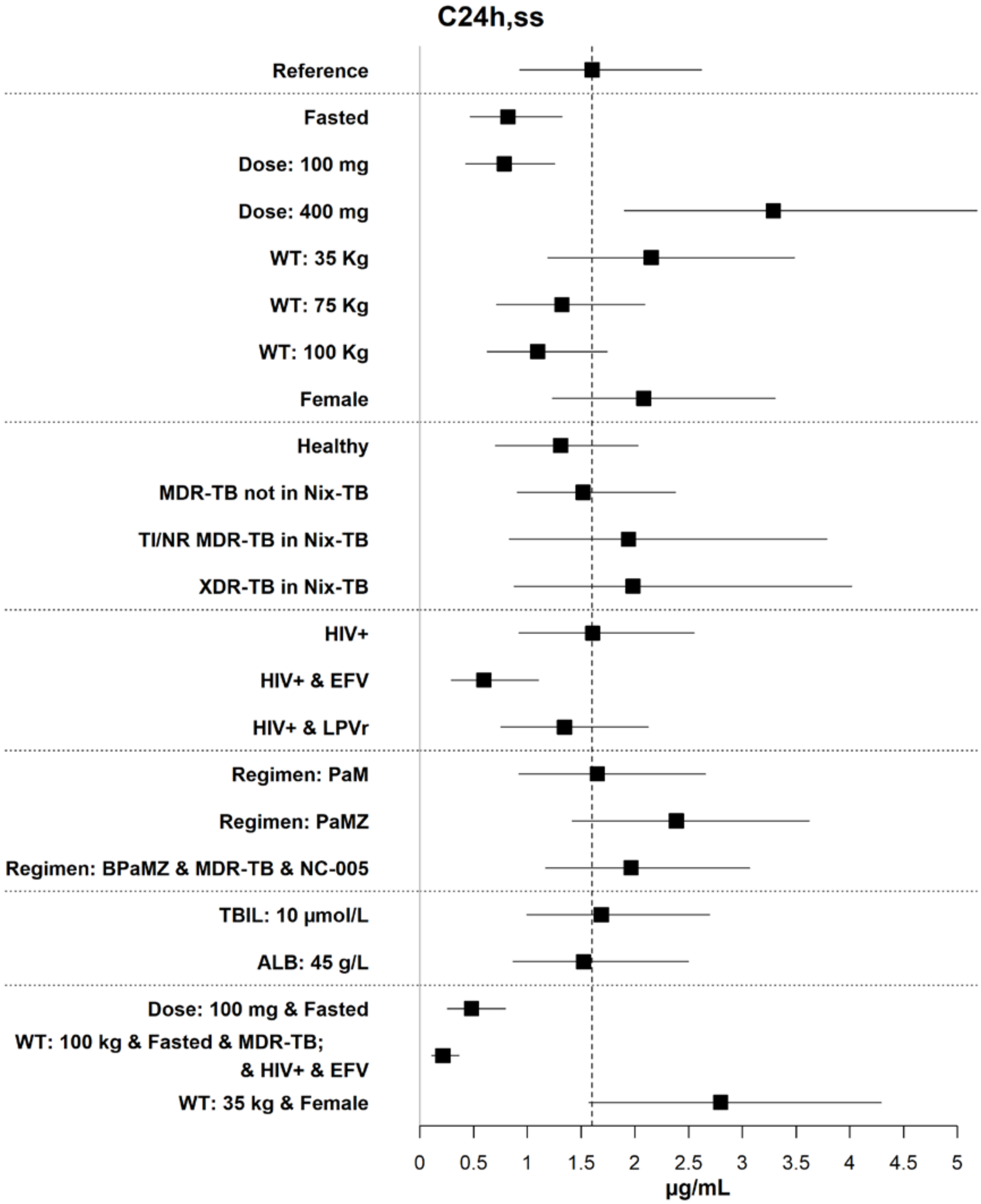

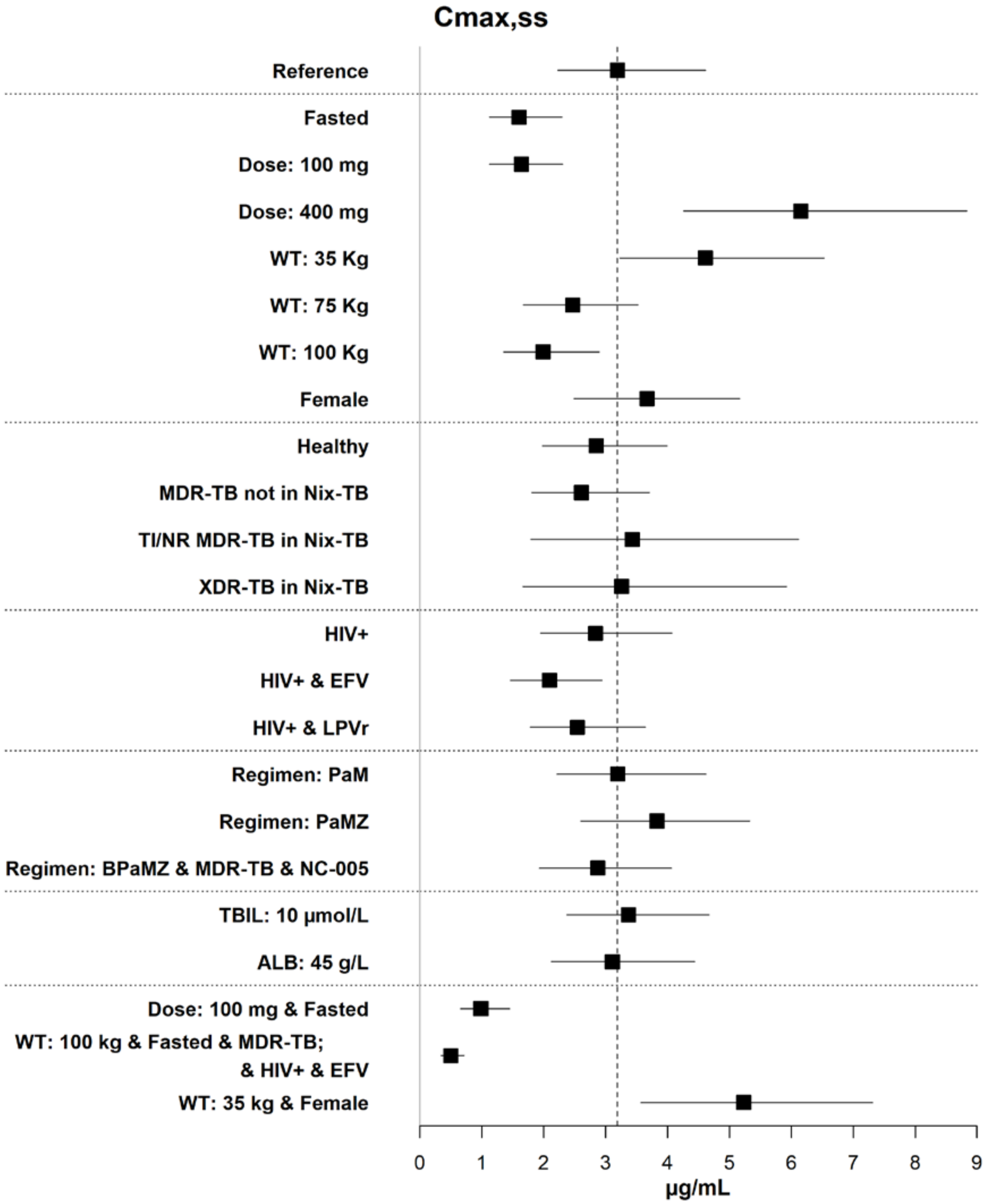

Cmax,ss
Reference
Fasted
Dose: 100 mg
Dose: 400 mg
WT: 35 Kg
WT: 75 Kg
WT: 100 Kg
Female
Healthy
MDR-TB not in Nix-TB
TI/NR MDR-TB in Nix-TB
XDR-TB in Nix-TB
HIV+
HIV+ & EFV
HIV+ & LPVr
Regimen: PaM
Regimen: PaMZ
Regimen: BPaMZ & MDR-TB & NC-005
TBIL: 10 μmol/L
ALB: 45 g/L
Dose: 100 mg & Fasted
WT: 100 kg & Fasted & MDR-TB;
& HIV+ & EFV
WT: 35 kg & Female
0
1
2
3
4
5
6
7
8
9
μg/mL

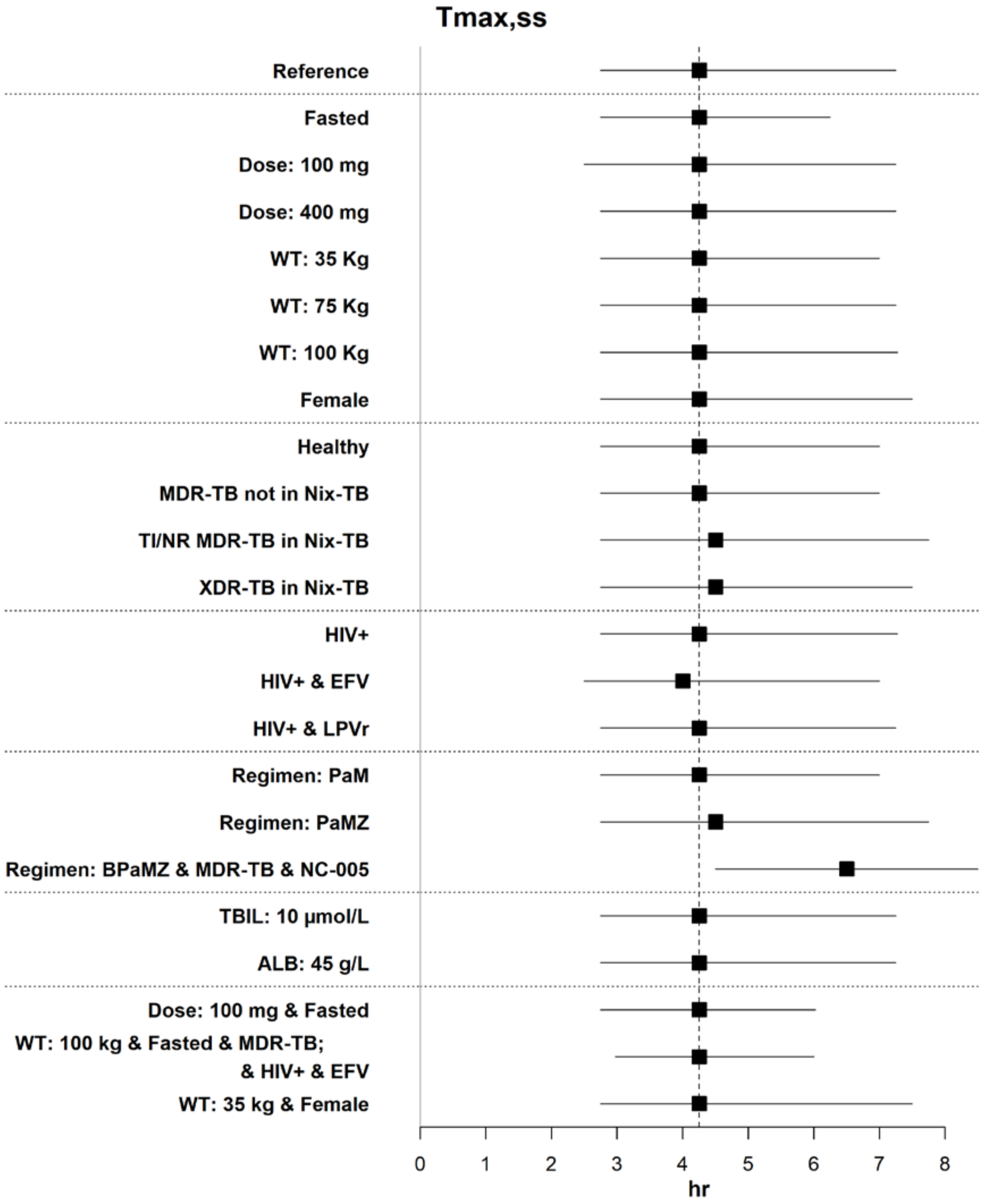
Tmax,ss
Reference
Fasted
Dose: 100 mg
Dose: 400 mg
WT: 35 Kg
WT: 75 Kg
WT: 100 Kg
Female
Healthy
MDR-TB not in Nix-TB
TI/NR MDR-TB in Nix-TB
XDR-TB in Nix-TB
HIV+
HIV+ & EFV
HIV+ & LPVr
Regimen: PaM
Regimen: PaMZ
Regimen: BPaMZ & MDR-TB & NC-005
TBIL: 10 μmol/L
ALB: 45 g/L
Dose: 100 mg & Fasted
WT: 100 kg & Fasted & MDR-TB;
& HIV+ & EFV
WT: 35 kg & Female
0
1
2
3
4
5
6
7
8
hr

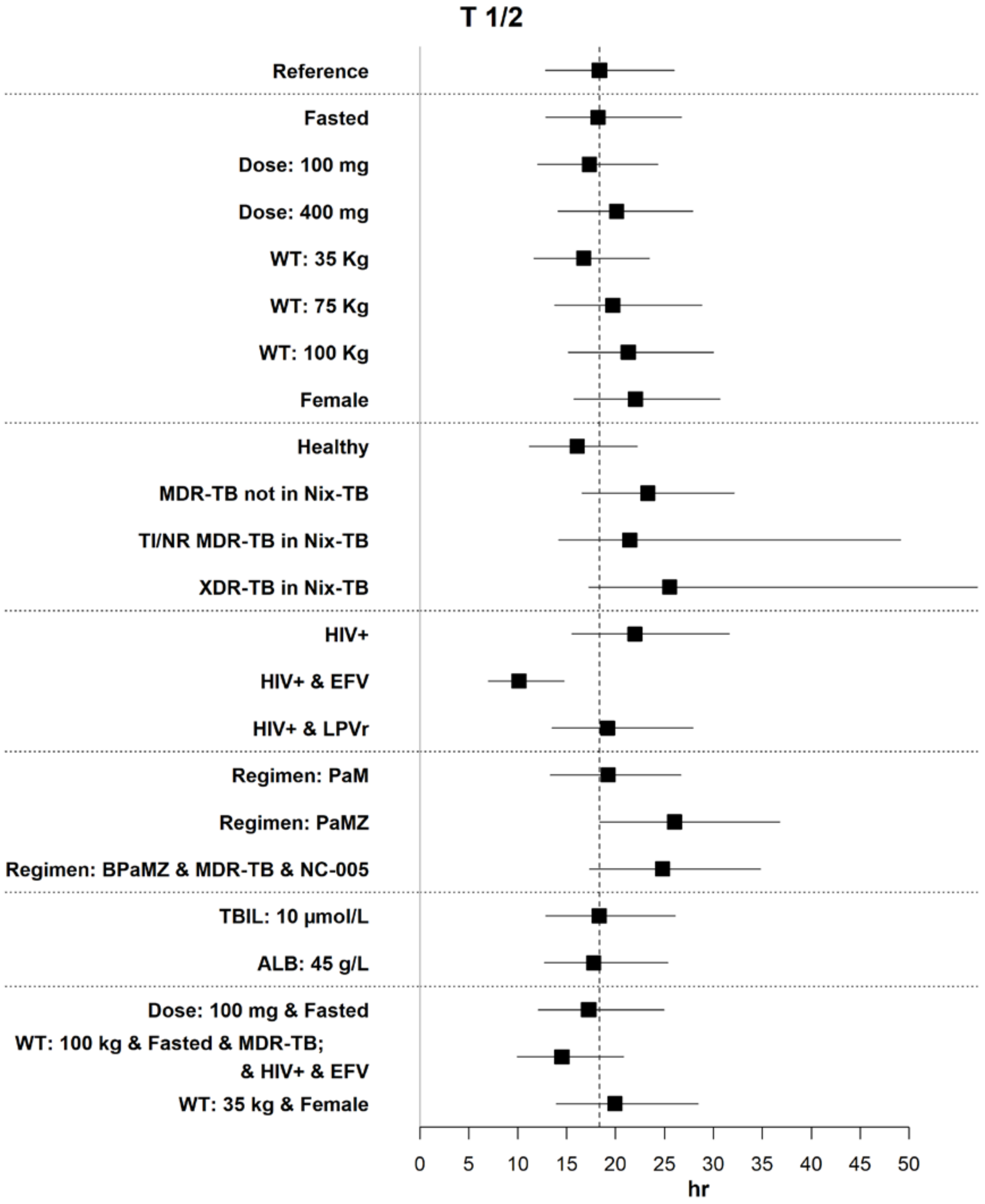
T 1/2
Reference
Fasted
Dose: 100 mg
Dose: 400 mg
WT: 35 Kg
WT: 75 Kg
WT: 100 Kg
Female
Healthy
MDR-TB not in Nix-TB
TI/NR MDR-TB in Nix-TB
XDR-TB in Nix-TB
HIV+
HIV+ & EFV
HIV+ & LPVr
Regimen: PaM
Regimen: PaMZ
Regimen: BPaMZ & MDR-TB & NC-005
TBIL: 10 µmol/L
ALB: 45 g/L
Dose: 100 mg & Fasted
WT: 100 kg & Fasted & MDR-TB;
& HIV+ & EFV
WT: 35 kg & Female
0 5 10 15 20 25 30 35 40 45 50
hr

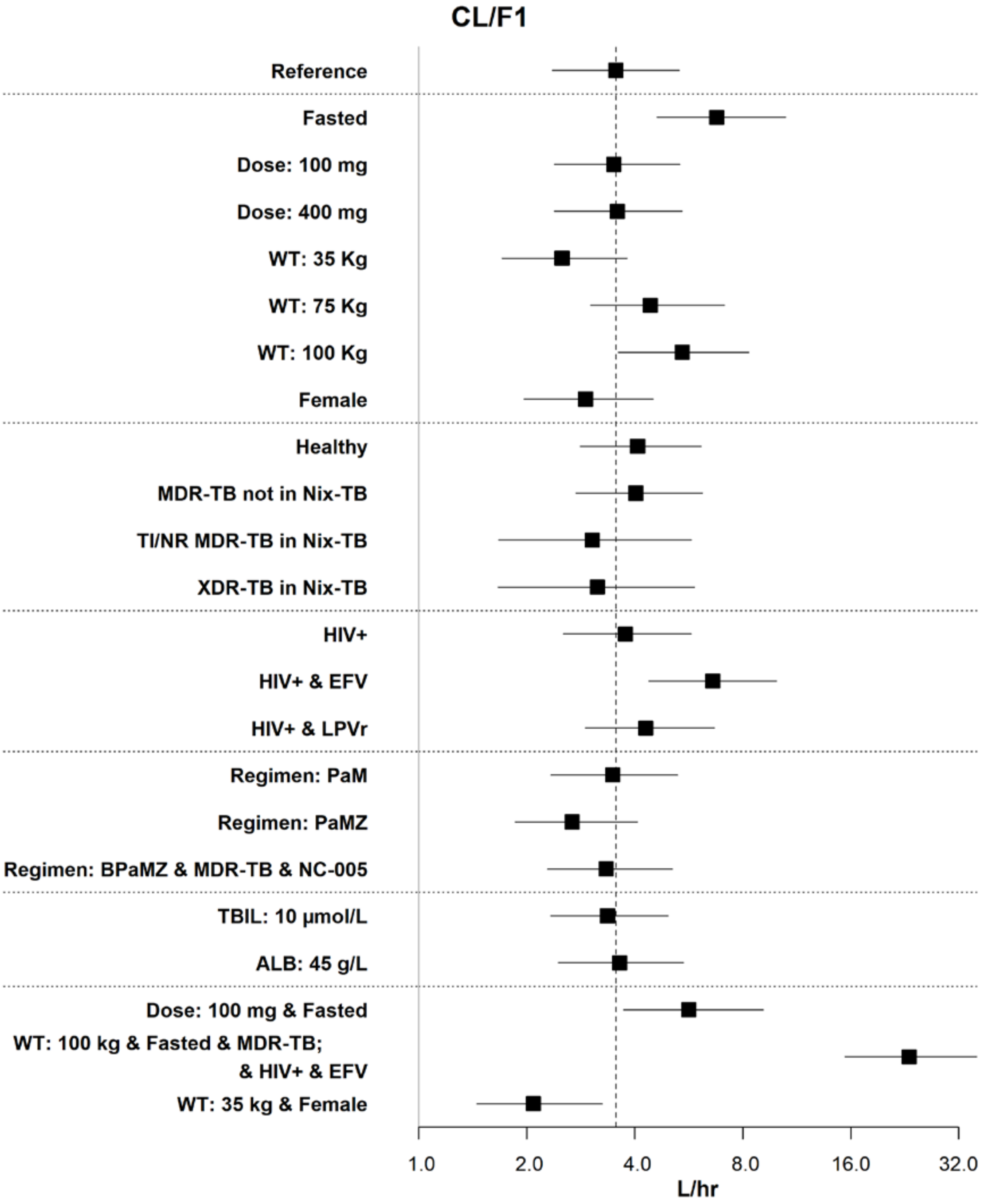

CL/F1
Reference
Fasted
Dose: 100 mg
Dose: 400 mg
WT: 35 Kg
WT: 75 Kg
WT: 100 Kg
Female
Healthy
MDR-TB not in Nix-TB
TI/NR MDR-TB in Nix-TB
XDR-TB in Nix-TB
HIV+
HIV+ & EFV
HIV+ & LPVr
Regimen: PaM
Regimen: PaMZ
Regimen: BPaMZ & MDR-TB & NC-005
TBIL: 10 μmol/L
ALB: 45 g/L
Dose: 100 mg & Fasted
WT: 100 kg & Fasted & MDR-TB;
& HIV+ & EFV
WT: 35 kg & Female
1.0
2.0
4.0
8.0
16.0
32.0
L/hr

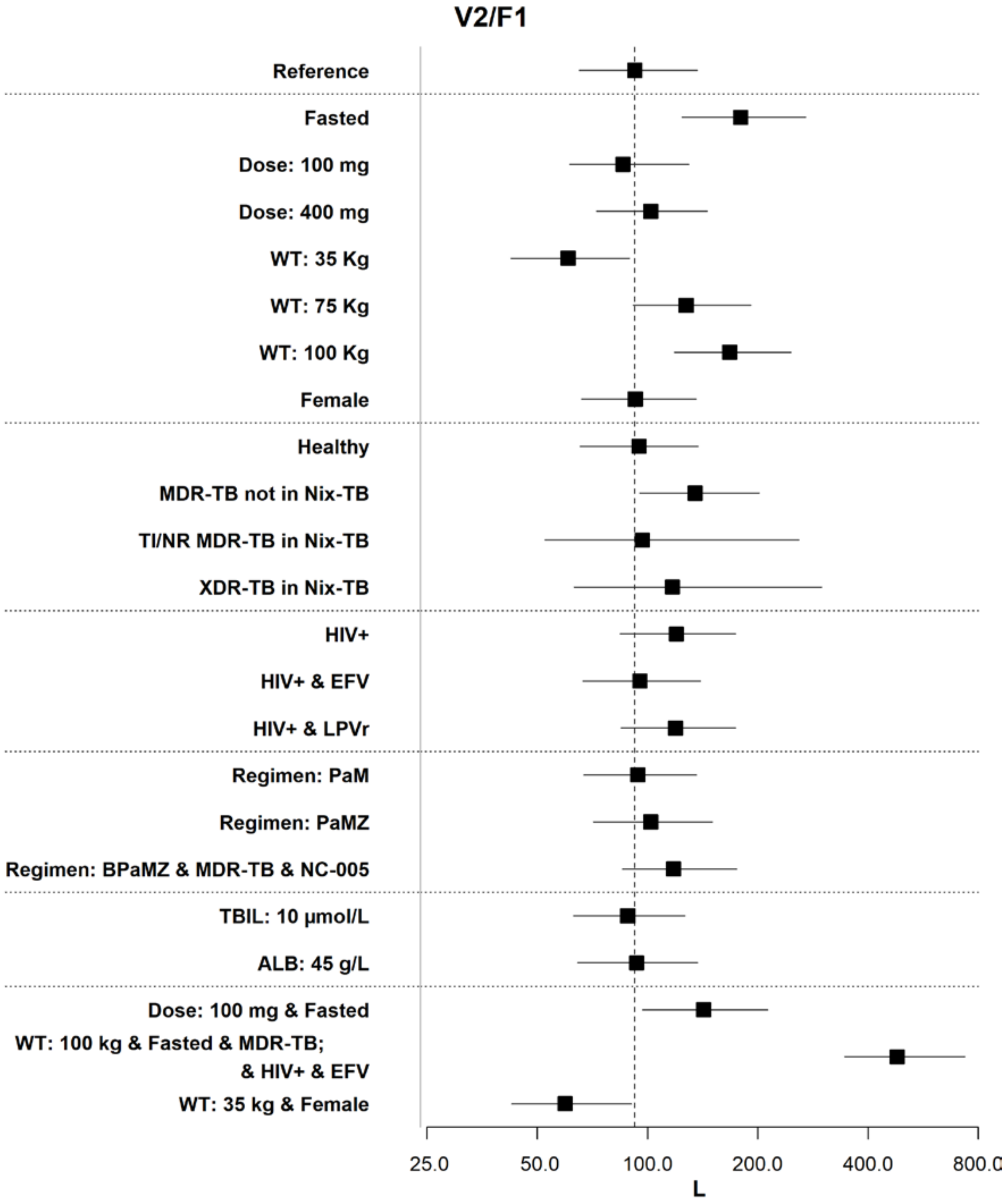
V2/F1
Reference
Fasted
Dose: 100 mg
Dose: 400 mg
WT: 35 Kg
WT: 75 Kg
WT: 100 Kg
Female
Healthy
MDR-TB not in Nix-TB
TI/NR MDR-TB in Nix-TB
XDR-TB in Nix-TB
HIV+
HIV+ & EFV
HIV+ & LPVr
Regimen: PaM
Regimen: PaMZ
Regimen: BPaMZ & MDR-TB & NC-005
TBIL: 10 µmol/L
ALB: 45 g/L
Dose: 100 mg & Fasted
WT: 100 kg & Fasted & MDR-TB;
& HIV+ & EFV
WT: 35 kg & Female
25.0
50.0
100.0
200.0
400.0
L

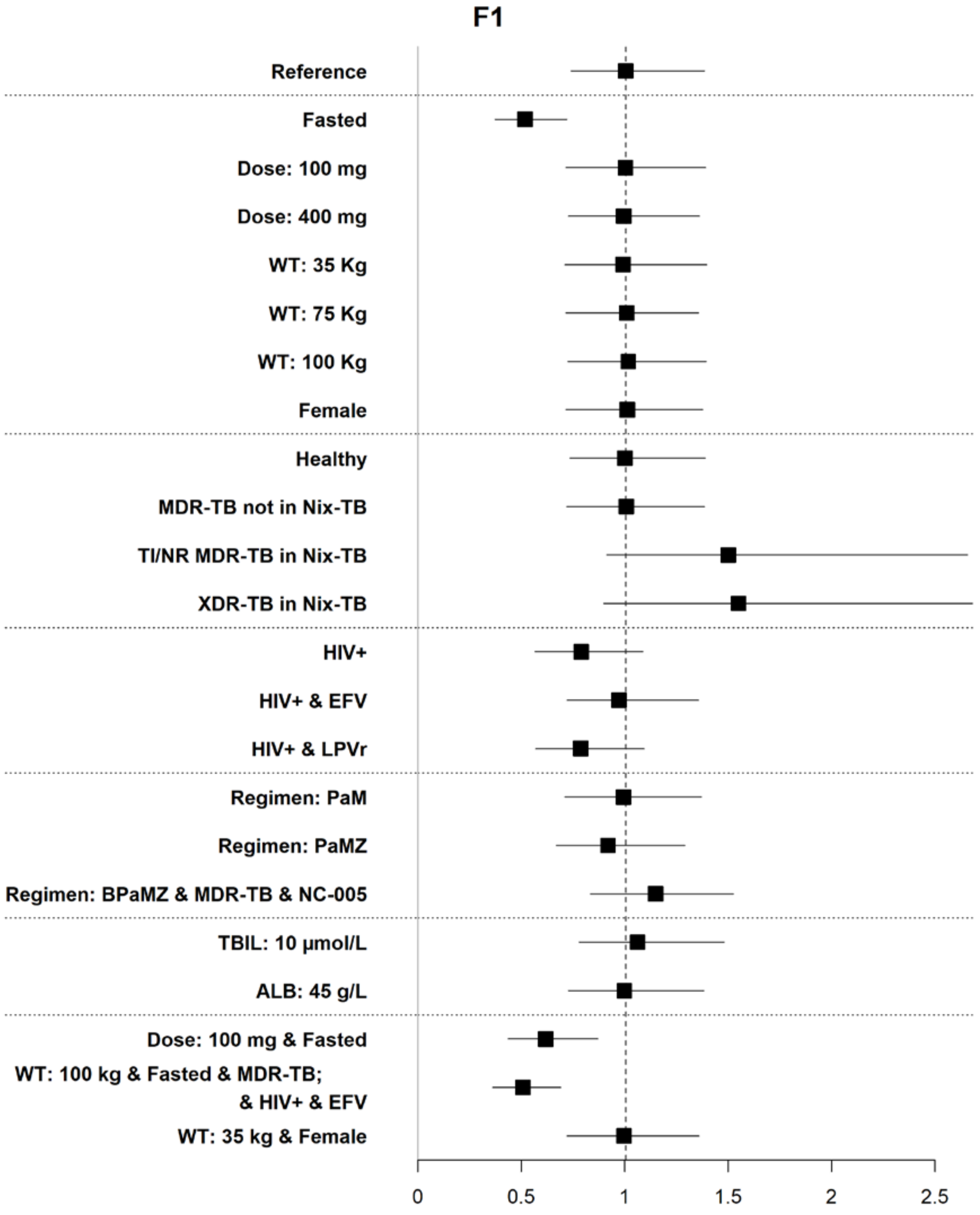
F1
Reference
Fasted
Dose: 100 mg
Dose: 400 mg
WT: 35 Kg
WT: 75 Kg
WT: 100 Kg
Female
Healthy
MDR-TB not in Nix-TB
TI/NR MDR-TB in Nix-TB
XDR-TB in Nix-TB
HIV+
HIV+ & EFV
HIV+ & LPVr
Regimen: PaM
Regimen: PaMZ
Regimen: BPaMZ & MDR-TB & NC-005
TBIL: 10 μmol/L
ALB: 45 g/L
Dose: 100 mg & Fasted
WT: 100 kg & Fasted & MDR-TB;
& HIV+ & EFV
WT: 35 kg & Female
0
0.5
1
1.5
2
2.5

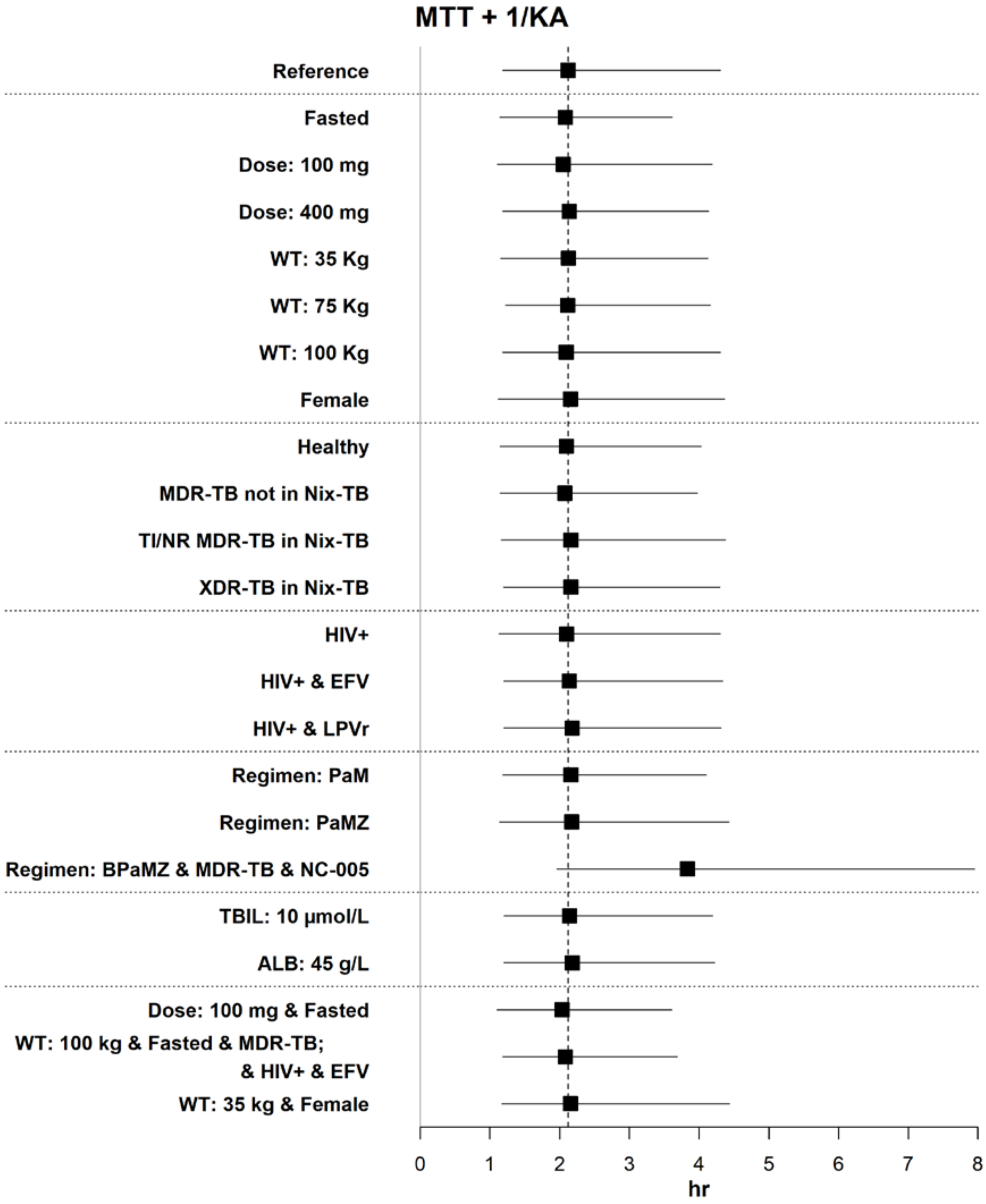
MTT + 1/KA
Reference
Fasted
Dose: 100 mg
Dose: 400 mg
WT: 35 Kg
WT: 75 Kg
WT: 100 Kg
Female
Healthy
MDR-TB not in Nix-TB
TI/NR MDR-TB in Nix-TB
XDR-TB in Nix-TB
HIV+
HIV+ & EFV
HIV+ & LPVr
Regimen: PaM
Regimen: PaMZ
Regimen: BPaMZ & MDR-TB & NC-005
TBIL: 10 μmol/L
ALB: 45 g/L
Dose: 100 mg & Fasted
WT: 100 kg & Fasted & MDR-TB;
& HIV+ & EFV
WT: 35 kg & Female
0
1
2
3
4
5
6
7
8
hr

Table S4 Summary of simulation results

Cavg, $C_{24h,ss}$, $C_{max,ss}$, $t_{max,ss}$, and steady state $t_{½}$, CL/F1, V2/F1, F1, and MTT +1/KA

**Cavg,ss (µg/mL)**							
	**Median**	**10th %tile**	**90th %tile**	**Mean**	**SD**	**GeoMean**	**CV%**
**Reference**	2.4	1.6	3.5	2.5	0.79	2.4	32
**Fasted**	1.2	0.79	1.8	1.3	0.40	1.2	32
**Dose: 100 mg**	1.2	0.78	1.7	1.2	0.39	1.2	32
**Dose: 400 mg**	4.7	3.1	7.0	4.9	1.6	4.7	32
**WT: 35 Kg**	3.3	2.2	4.9	3.5	1.1	3.3	32
**WT: 75 Kg**	1.9	1.2	2.8	1.9	0.63	1.8	32
**WT: 100 Kg**	1.5	1.0	2.3	1.6	0.51	1.5	32
**Female**	2.9	1.9	4.2	3.0	0.93	2.8	32
**Healthy**	2.0	1.4	3.0	2.1	0.68	2.0	32
**MDR-TB not in Nix-TB**	2.1	1.4	3.0	2.1	0.68	2.0	32
**TI/NR MDR-TB in Nix-TB**	2.7	1.5	5.0	3.0	1.5	2.7	50
**XDR-TB in Nix-TB**	2.7	1.4	5.0	3.0	1.5	2.7	51
**HIV+**	2.2	1.5	3.3	2.3	0.73	2.2	32
**HIV+ & EFV**	1.3	0.84	1.9	1.3	0.43	1.3	32
**HIV+ & LPVr**	1.9	1.3	2.9	2.0	0.65	1.9	32
**Regimen: PaM**	2.4	1.6	3.6	2.5	0.80	2.4	32
**Regimen: PaMZ**	3.1	2.1	4.5	3.2	1.0	3.1	31
**Regimen: BPaMZ & MDR-TB & NC-005**	2.5	1.6	3.7	2.6	0.82	2.5	32
**TBIL: 10 µmol/L**	2.5	1.7	3.6	2.6	0.80	2.5	31
**ALB: 45 g/L**	2.3	1.5	3.4	2.4	0.77	2.3	32
**Dose: 100 mg; Fasted**	0.74	0.46	1.1	0.77	0.27	0.73	36
**WT: 100 kg; Fasted; MDR-TB; HIV+ & EFV**	0.36	0.23	0.54	0.37	0.12	0.35	33
**WT: 35 kg; Female**	4.0	2.6	5.8	4.1	1.3	3.9	31

C24h,ss µg/mL							
	**Median**	**10th %tile**	**90th %tile**	**Mean**	**SD**	**GeoMean**	**CV%**
**Reference**	1.6	0.93	2.6	1.7	0.68	1.6	40
**Fasted**	0.82	0.47	1.3	0.86	0.34	0.79	39
**Dose: 100 mg**	0.79	0.43	1.3	0.82	0.33	0.76	40
**Dose: 400 mg**	3.3	1.9	5.2	3.5	1.4	3.2	39
**WT: 35 Kg**	2.2	1.2	3.5	2.3	0.94	2.1	41
**WT: 75 Kg**	1.3	0.71	2.1	1.4	0.55	1.3	40
**WT: 100 Kg**	1.1	0.63	1.7	1.2	0.44	1.1	39
**Female**	2.1	1.2	3.3	2.2	0.81	2	37
**Healthy**	1.3	0.7	2	1.4	0.56	1.2	42
**MDR-TB not in Nix-TB**	1.5	0.91	2.4	1.6	0.59	1.5	37
**TI/NR MDR-TB in Nix-TB**	1.9	0.83	3.8	2.1	1.2	0.91	58
**XDR-TB in Nix-TB**	2	0.88	4	2.2	1.3	1.1	58
**HIV+**	1.6	0.92	2.6	1.7	0.64	1.6	38
**HIV+ & EFV**	0.6	0.29	1.1	0.66	0.33	0.57	51
**HIV+ & LPVr**	1.3	0.75	2.1	1.4	0.56	1.3	40
**Regimen: PaM**	1.7	0.92	2.7	1.7	0.7	1.6	40
**Regimen: PaMZ**	2.4	1.4	3.6	2.5	0.9	2.3	36
**Regimen: BPaMZ & MDR-TB & NC-005**	2	1.2	3.1	2.1	0.77	1.9	37
**TBIL: 10 µmol/L**	1.7	1	2.7	1.8	0.69	1.7	39
**ALB: 45 g/L**	1.5	0.87	2.5	1.6	0.65	1.5	40
**Dose: 100 mg & Fasted**	0.48	0.25	0.79	0.51	0.23	0.46	45
**WT: 100 kg & Fasted & MDR-TB & HIV+ & EFV**	0.22	0.11	0.36	0.23	0.1	0.21	44
**WT: 35 kg & Female**	2.8	1.6	4.3	2.9	1.1	2.7	38

**Cmax,ss µg/mL**							
	**Median**	**10th %tile**	**90th %tile**	**Mean**	**SD**	**GeoMean**	**CV%**
**Reference**	3.2	2.2	4.6	3.3	0.96	3.2	29
**Fasted**	1.6	1.1	2.3	1.7	0.47	1.6	28
**Dose: 100 mg**	1.6	1.1	2.3	1.7	0.47	1.6	28
**Dose: 400 mg**	6.2	4.3	8.8	6.4	1.8	6.2	29
**WT: 35 Kg**	4.6	3.2	6.5	4.7	1.3	4.6	28
**WT: 75 Kg**	2.5	1.7	3.5	2.5	0.73	2.4	29
**WT: 100 Kg**	2	1.4	2.9	2	0.59	2	29
**Female**	3.7	2.5	5.2	3.8	1.1	3.6	28
**Healthy**	2.9	2	4	2.9	0.84	2.8	28
**MDR-TB not in Nix-TB**	2.6	1.8	3.7	2.7	0.78	2.6	29
**TI/NR MDR-TB in Nix-TB**	3.4	1.8	6.1	3.7	1.9	1.6	51
**XDR-TB in Nix-TB**	3.3	1.7	5.9	3.6	1.8	1.7	51
**HIV+**	2.8	2	4.1	2.9	0.84	2.8	28
**HIV+ & EFV**	2.1	1.5	2.9	2.2	0.6	2.1	28
**HIV+ & LPVr**	2.5	1.8	3.6	2.7	0.76	2.6	29
**Regimen: PaM**	3.2	2.2	4.6	3.3	0.94	3.2	28
**Regimen: PaMZ**	3.8	2.6	5.3	3.9	1.1	3.8	29
**Regimen: BPaMZ & MDR-TB & NC-005**	2.9	1.9	4.1	3	0.86	2.8	29
**TBIL: 10 µmol/L**	3.4	2.4	4.7	3.5	0.94	3.4	27
**ALB: 45 g/L**	3.1	2.1	4.4	3.2	0.93	3.1	29
**Dose: 100 mg & Fasted**	0.99	0.66	1.5	1	0.32	0.98	31
**WT: 100 kg & Fasted & MDR-TB & HIV+ & EFV**	0.5	0.34	0.72	0.52	0.15	0.5	28
**WT: 35 kg & Female**	5.2	3.6	7.3	5.4	1.5	5.2	28

Tmax,ss hr							
	**Median**	**10th %tile**	**90th %tile**	**Mean**	**SD**	**GeoMean**	**CV%**
**Reference**	4.2	2.8	7.2	4.7	2.1	4.4	44
**Fasted**	4.2	2.8	6.2	4.5	1.3	4.3	29
**Dose: 100 mg**	4.2	2.5	7.2	4.6	2	4.2	43
**Dose: 400 mg**	4.2	2.8	7.2	4.8	2	4.4	42
**WT: 35 Kg**	4.2	2.8	7	4.6	2	4.3	44
**WT: 75 Kg**	4.2	2.8	7.2	4.7	1.9	4.4	40
**WT: 100 Kg**	4.2	2.8	7.3	4.7	2	4.4	42
**Female**	4.2	2.8	7.5	4.8	2.2	4.4	45
**Healthy**	4.2	2.8	7	4.6	2	4.3	42
**MDR-TB not in Nix-TB**	4.2	2.8	7	4.7	1.9	4.3	41
**TI/NR MDR-TB in Nix-TB**	4.5	2.8	7.8	4.9	2.2	4.5	44
**XDR-TB in Nix-TB**	4.5	2.8	7.5	4.9	2.1	4.5	43
**HIV+**	4.2	2.8	7.3	4.8	2.1	4.4	44
**HIV+ & EFV**	4	2.5	7	4.5	2	4.2	43
**HIV+ & LPVr**	4.2	2.8	7.2	4.8	2	4.5	41
**Regimen: PaM**	4.2	2.8	7	4.7	1.9	4.4	40
**Regimen: PaMZ**	4.5	2.8	7.8	4.9	2.1	4.5	43
**Regimen: BPaMZ & MDR-TB & NC-005**	6.5	4.5	8.5	6.5	1.5	6.3	23
**TBIL: 10 µmol/L**	4.2	2.8	7.2	4.7	1.8	4.4	39
**ALB: 45 g/L**	4.2	2.8	7.2	4.8	2.1	4.4	43
**Dose: 100 mg & Fasted**	4.2	2.8	6	4.4	1.3	4.2	30
**WT: 100 kg & Fasted & MDR-TB & HIV+ & EFV**	4.2	3	6	4.4	1.4	4.2	31
**WT: 35 kg & Female**	4.2	2.8	7.5	4.8	2	4.4	42

T 1/2 hr							
	Median	10th %tile	90th %tile	Mean	SD	GeoMean	CV%
**Reference**	18	13	26	19	7	18	36
**Fasted**	18	13	27	20	13	18	67
**Dose: 100 mg**	17	12	24	18	5.7	17	32
**Dose: 400 mg**	20	14	28	21	7.2	20	35
**WT: 35 Kg**	17	12	23	18	15	17	85
**WT: 75 Kg**	20	14	29	21	9.6	20	46
**WT: 100 Kg**	21	15	30	22	7.4	21	33
**Female**	22	16	31	23	8.7	22	37
**Healthy**	16	11	22	17	7.5	16	45
**MDR-TB not in Nix-TB**	23	17	32	24	8.7	23	35
**TI/NR MDR-TB in Nix-TB**	21	14	49	3.90E+96	1.20E+98	54	3200
**XDR-TB in Nix-TB**	26	17	57	1.60E+49	4.20E+50	58	2600
**HIV+**	22	16	32	23	7.7	22	33
**HIV+ & EFV**	10	7	15	11	3.8	10	35
**HIV+ & LPVr**	19	13	28	20	7.4	19	36
**Regimen: PaM**	19	13	27	20	6.1	19	31
**Regimen: PaMZ**	26	18	37	27	8.9	26	32
**Regimen: BPaMZ & MDR-TB & NC-005**	25	17	35	26	8.4	25	33
**TBIL: 10 µmol/L**	18	13	26	19	6.4	18	33
**ALB: 45 g/L**	18	13	25	19	6.3	18	34
**Dose: 100 mg & Fasted**	17	12	25	18	6.5	17	36
**WT: 100 kg & Fasted & MDR-TB & HIV+ & EFV**	15	9.9	21	15	5.3	14	35
**WT: 35 kg & Female**	20	14	28	21	7.4	20	35

**CL/F1 L/hr**							
	**Median**	**10th %tile**	**90th %tile**	**Mean**	**SD**	**GeoMean**	**CV%**
**Reference**	3.5	2.4	5.3	3.7	1.3	3.5	34
**Fasted**	6.8	4.6	11	7.3	2.6	6.9	36
**Dose: 100 mg**	3.5	2.4	5.4	3.7	1.2	3.5	33
**Dose: 400 mg**	3.6	2.4	5.4	3.8	1.3	3.6	33
**WT: 35 Kg**	2.5	1.7	3.8	2.7	0.91	2.5	34
**WT: 75 Kg**	4.4	3	7.1	4.8	1.7	4.5	35
**WT: 100 Kg**	5.4	3.6	8.3	5.8	2	5.5	35
**Female**	2.9	2	4.5	3.1	1.1	3	34
**Healthy**	4.1	2.8	6.1	4.4	1.5	4.1	35
**MDR-TB not in Nix-TB**	4	2.7	6.2	4.3	1.4	4.1	33
**TI/NR MDR-TB in Nix-TB**	3	1.7	5.7	3.5	2	3.1	56
**XDR-TB in Nix-TB**	3.2	1.7	5.9	3.6	2.5	3.1	69
**HIV+**	3.8	2.5	5.7	4	1.3	3.8	33
**HIV+ & EFV**	6.6	4.4	9.9	7	2.4	6.6	35
**HIV+ & LPVr**	4.3	2.9	6.7	4.6	1.5	4.3	34
**Regimen: PaM**	3.5	2.3	5.3	3.7	1.3	3.5	36
**Regimen: PaMZ**	2.7	1.9	4.1	2.9	0.97	2.7	34
**Regimen: BPaMZ & MDR-TB & NC-005**	3.3	2.3	5.1	3.6	1.2	3.4	34
**TBIL: 10 µmol/L**	3.4	2.3	5	3.5	1.1	3.4	32
**ALB: 45 g/L**	3.6	2.4	5.5	3.9	1.3	3.7	34
**Dose: 100 mg & Fasted**	5.6	3.7	9.1	6.1	2.3	5.7	37
**WT: 100 kg & Fasted & MDR-TB & HIV+ & EFV**	23	15	36	25	8.8	24	35
**WT: 35 kg & Female**	2.1	1.4	3.2	2.3	0.81	2.1	36

V2/F1 L							
	**Median**	**10th %tile**	**90th %tile**	**Mean**	**SD**	**GeoMean**	**CV%**
**Reference**	92	65	140	99	36	94	36
**Fasted**	180	120	270	200	130	180	64
**Dose: 100 mg**	86	61	130	92	33	88	35
**Dose: 400 mg**	100	73	150	110	36	100	34
**WT: 35 Kg**	61	42	89	65	39	61	59
**WT: 75 Kg**	130	91	190	140	54	130	40
**WT: 100 Kg**	170	120	250	180	62	170	35
**Female**	93	66	140	100	49	95	49
**Healthy**	95	65	140	100	40	95	40
**MDR-TB not in Nix-TB**	130	95	200	140	52	140	36
**TI/NR MDR-TB in Nix-TB**	97	53	260	5.10E+97	1.60E+99	240	3200
**XDR-TB in Nix-TB**	120	63	300	7.70E+49	2.00E+51	260	2500
**HIV+**	120	84	170	130	44	120	35
**HIV+ & EFV**	95	67	140	100	39	97	38
**HIV+ & LPVr**	120	85	170	130	40	120	32
**Regimen: PaM**	94	67	140	100	31	96	31
**Regimen: PaMZ**	100	71	150	110	35	100	32
**Regimen: BPaMZ & MDR-TB & NC-005**	120	86	180	130	46	120	36
**TBIL: 10 µmol/L**	88	63	130	93	29	89	31
**ALB: 45 g/L**	93	65	140	99	37	94	37
**Dose: 100 mg & Fasted**	140	97	210	150	55	140	36
**WT: 100 kg & Fasted & MDR-TB & HIV+ & EFV**	480	350	740	520	210	490	40
**WT: 35 kg & Female**	60	43	91	65	25	61	39

F1							
	**Median**	**10th %tile**	**90th %tile**	**Mean**	**SD**	**GeoMean**	**CV%**
**Reference**	1	0.74	1.4	1	0.27	1	26
**Fasted**	0.52	0.37	0.72	0.54	0.15	0.52	27
**Dose: 100 mg**	1	0.72	1.4	1	0.27	1	26
**Dose: 400 mg**	0.99	0.73	1.4	1	0.27	1	26
**WT: 35 Kg**	0.99	0.71	1.4	1	0.26	0.99	26
**WT: 75 Kg**	1	0.71	1.4	1	0.25	1	25
**WT: 100 Kg**	1	0.73	1.4	1	0.27	1	26
**Female**	1	0.72	1.4	1	0.26	1	25
**Healthy**	1	0.73	1.4	1	0.28	1	27
**MDR-TB not in Nix-TB**	1	0.72	1.4	1	0.26	1	26
**TI/NR MDR-TB in Nix-TB**	1.5	0.91	2.7	1.7	0.73	1.5	44
**XDR-TB in Nix-TB**	1.5	0.9	2.7	1.7	0.75	1.5	45
**HIV+**	0.79	0.57	1.1	0.81	0.21	0.79	26
**HIV+ & EFV**	0.97	0.72	1.4	1	0.26	0.98	25
**HIV+ & LPVr**	0.79	0.57	1.1	0.82	0.21	0.79	26
**Regimen: PaM**	0.99	0.71	1.4	1	0.25	0.99	25
**Regimen: PaMZ**	0.92	0.67	1.3	0.95	0.25	0.92	27
**Regimen: BPaMZ & MDR-TB & NC-005**	1.1	0.83	1.5	1.2	0.28	1.1	24
**TBIL: 10 µmol/L**	1.1	0.78	1.5	1.1	0.27	1.1	25
**ALB: 45 g/L**	1	0.73	1.4	1	0.27	1	26
**Dose: 100 mg & Fasted**	0.62	0.44	0.87	0.64	0.19	0.62	29
**WT: 100 kg & Fasted & MDR-TB & HIV+ & EFV**	0.51	0.36	0.69	0.52	0.14	0.5	26
**WT: 35 kg & Female**	1	0.72	1.4	1	0.26	1	26

MTT + 1/KA hr							
	**Median**	**10th %tile**	**90th %tile**	**Mean**	**SD**	**GeoMean**	**CV%**
**Reference**	2.1	1.2	4.3	2.6	1.7	2.2	67
**Fasted**	2.1	1.1	3.6	2.3	1.1	2.1	46
**Dose: 100 mg**	2.1	1.1	4.2	2.4	1.5	2.1	61
**Dose: 400 mg**	2.1	1.2	4.1	2.5	1.5	2.2	59
**WT: 35 Kg**	2.1	1.2	4.1	2.5	1.8	2.2	71
**WT: 75 Kg**	2.1	1.2	4.2	2.5	1.4	2.2	56
**WT: 100 Kg**	2.1	1.2	4.3	2.5	1.5	2.2	60
**Female**	2.2	1.1	4.4	2.6	1.7	2.2	65
**Healthy**	2.1	1.1	4	2.5	1.5	2.2	62
**MDR-TB not in Nix-TB**	2.1	1.1	4	2.4	1.4	2.1	58
**TI/NR MDR-TB in Nix-TB**	2.2	1.2	4.4	2.6	1.6	2.2	63
**XDR-TB in Nix-TB**	2.2	1.2	4.3	2.5	1.4	2.2	57
**HIV+**	2.1	1.1	4.3	2.5	1.6	2.2	64
**HIV+ & EFV**	2.1	1.2	4.3	2.6	1.6	2.2	62
**HIV+ & LPVr**	2.2	1.2	4.3	2.6	1.5	2.3	60
**Regimen: PaM**	2.2	1.2	4.1	2.5	1.4	2.2	56
**Regimen: PaMZ**	2.2	1.1	4.4	2.6	1.6	2.2	61
**Regimen: BPaMZ & MDR-TB & NC-005**	3.8	2	8	4.6	2.8	3.9	60
**TBIL: 10 μmol/L**	2.1	1.2	4.2	2.5	1.4	2.2	54
**ALB: 45 g/L**	2.2	1.2	4.2	2.6	1.6	2.2	64
**Dose: 100 mg & Fasted**	2	1.1	3.6	2.3	1.1	2	48
**WT: 100 kg & Fasted & MDR-TB & HIV+ & EFV**	2.1	1.2	3.7	2.3	1.2	2.1	51
**WT: 35 kg & Female**	2.2	1.2	4.4	2.6	1.5	2.2	57

FINAL MODEL DIAGNOSTICS

Throughout the diagnostic plots, study numbers 1 – 10 indicate Studies CL-001 – CL-010, and 12 indicates DMID 10-0058. Study numbers 101 – 106 are Studies NC-001 – NC-006. Study number 201 is the Nix-TB study.

Models were evaluated by graphical diagnostics. Two types of diagnostics were used: prediction-based and simulation-based (Nguyen et al 2017).

Prediction-based diagnostics (Figures 3-8) included scatterplots or boxplots with observed values, residuals, or estimated individual random effects ($\hat{\eta}$'s) on the y-axis and with predicted values, time, or covariates on the x-axis. Prediction-based diagnostics assess the adequacy of modeled population and covariate effects and of modeled correlation structure for the between-subject random effects

Simulation-based diagnostics (Figures 9-13) consisted of prediction-corrected visual predictive checks (pcVPCs). 5th, 50th, and 95th percentiles of the prediction-corrected observations were plotted as dashed lines. 95% confidence regions for the 5th, 50th, and 95th percentiles of the prediction-corrected simulations were plotted as shaded areas. For pcVPCs examining the Nix-TB sub-study data alone, the 10th, 50th, and 90th percentiles were used instead due to the relatively small sample size. pcVPCs provided a holistic assessment of the ability of the model to replicate the observed trends and marginal variability in the concentration-time profile (Nguyen et al 2017).

Figure S4 Prediction-based diagnostics, set 1

OBS vs PRED, OBS vs IPRED; CWRES vs PRED, CWRES vs time after dose, CWRES vs STUDY, CWRES vs DOSE;   IWRES vs IPRED, IWRES vs time after dose, IWRES vs STUDY, IWRES vs DOSE, ηˇs vs STUDY, ηˇs vs DOSE, individual plots

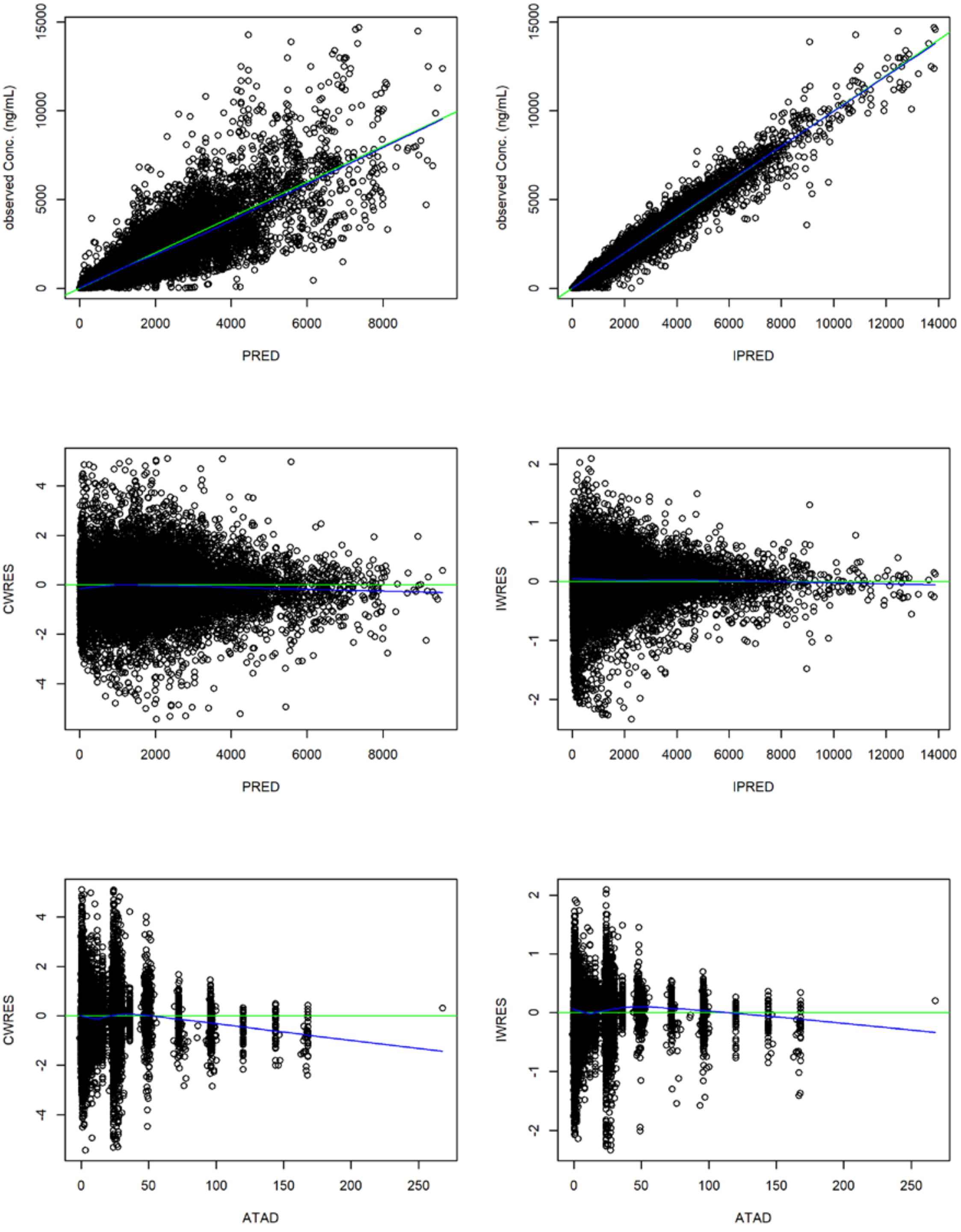

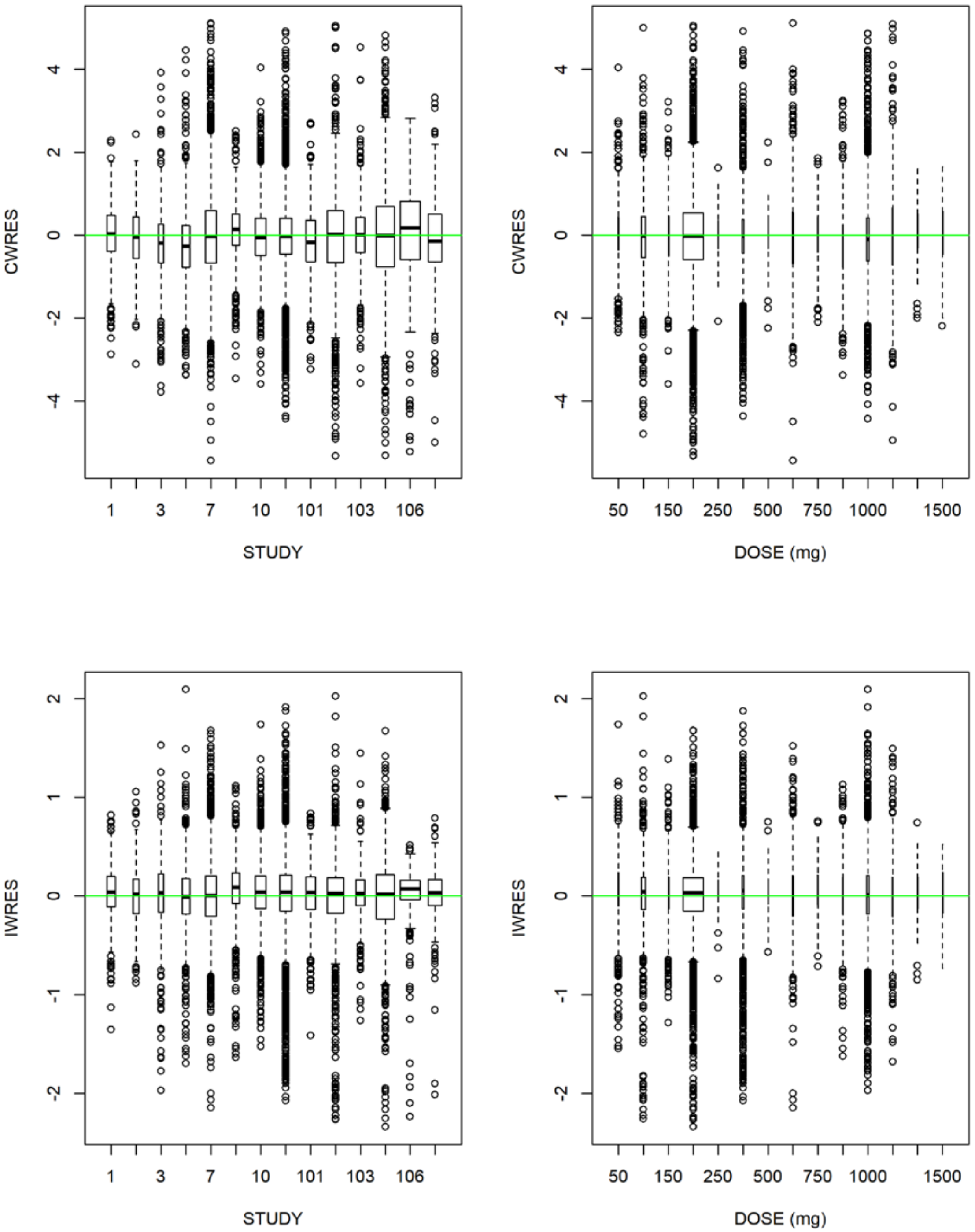
CWRES
STUDY
DOSE (mg)
IWRES
1 3 7 10 101 103 106
50 150 250 500 750 1000 1500

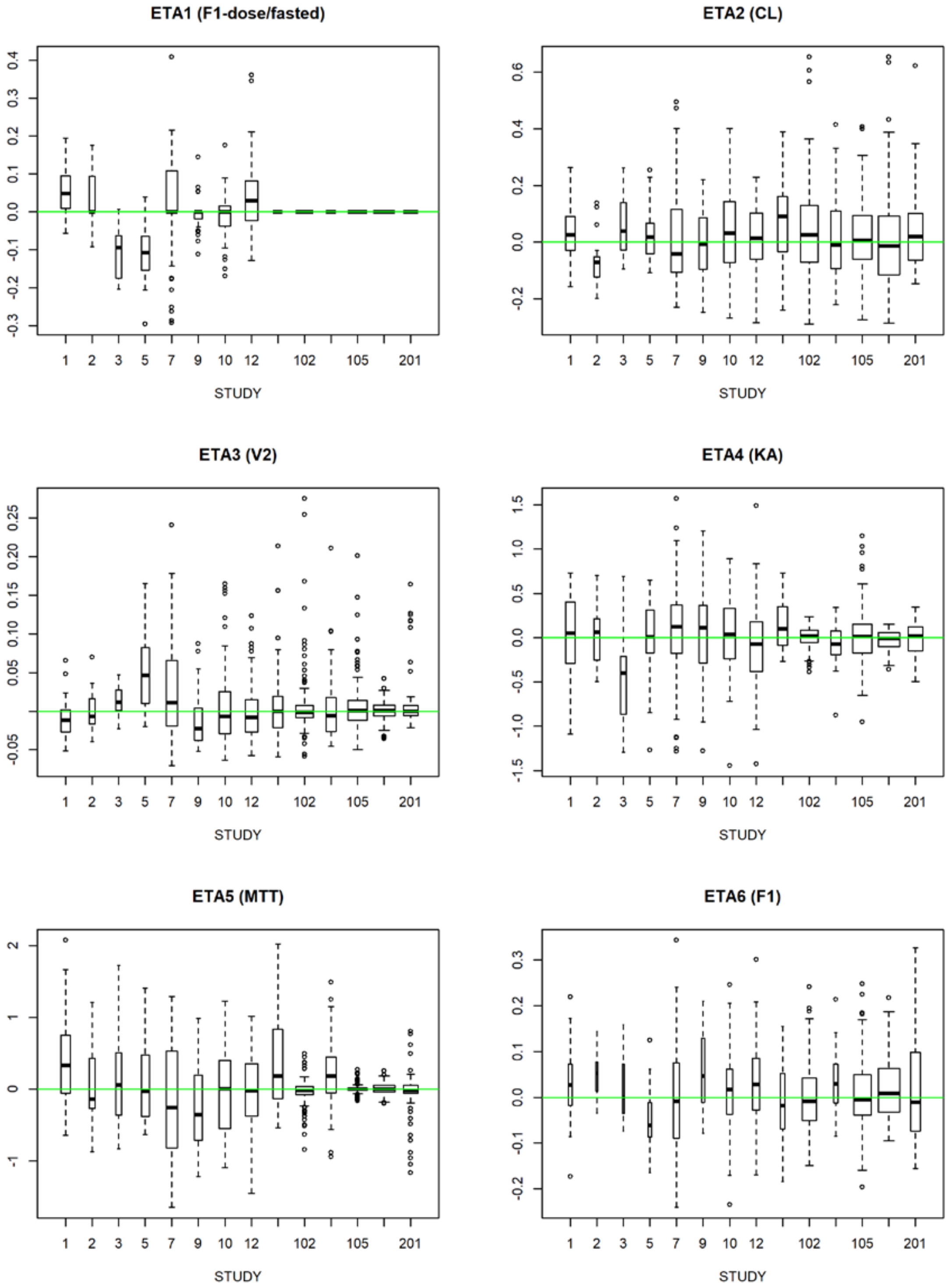

ETA1 (F1-dose/fasted)
0.4
0.3
0.2
0.1
0.0
-0.1
-0.2
-0.3
1 2 3 5 7 9 10 12 102 105 201
STUDY
ETA2 (CL)
0.6
0.4
0.2
0.0
-0.2
1 2 3 5 7 9 10 12 102 105 201
STUDY
ETA3 (V2)
0.25
0.20
0.15
0.10
0.05
-0.05
1 2 3 5 7 9 10 12 102 105 201
STUDY
ETA4 (KA)
1.5
1.0
0.5
0.0
-0.5
-1.0
-1.5
1 2 3 5 7 9 10 12 102 105 201
STUDY
ETA5 (MTT)
2
1
0
-1
1 2 3 5 7 9 10 12 102 105 201
STUDY
ETA6 (F1)
0.3
0.2
0.1
0.0
-0.1
-0.2
1 2 3 5 7 9 10 12 102 105 201
STUDY

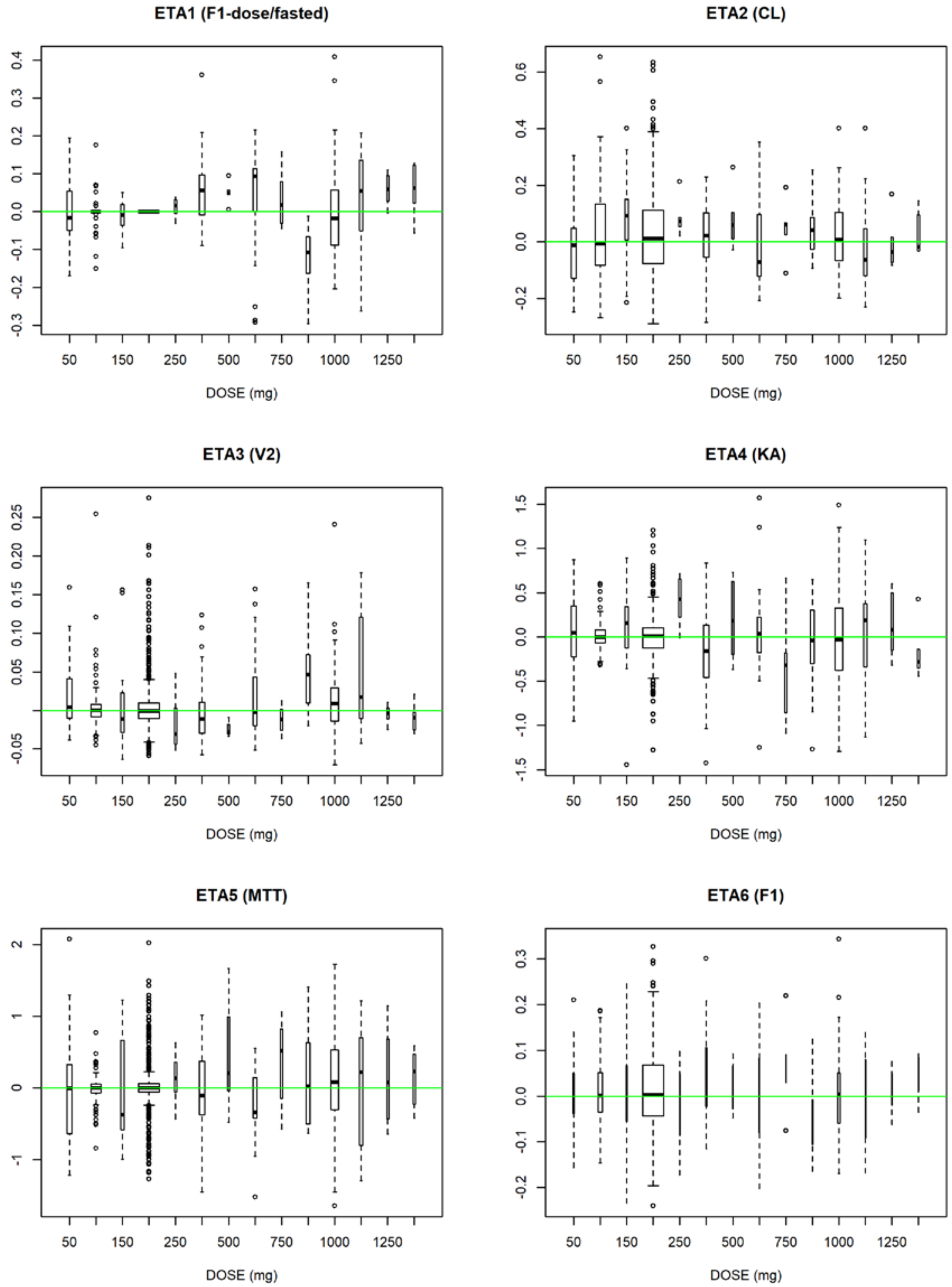
ETA1 (F1-dose/fasted)
0.4
0.3
0.2
0.1
0.0
-0.1
-0.2
-0.3
50 150 250 500 750 1000 1250
DOSE (mg)
ETA2 (CL)
0.6
0.4
0.2
0.0
-0.2
50 150 250 500 750 1000 1250
DOSE (mg)
ETA3 (V2)
0.25
0.20
0.15
0.10
0.05
-0.05
50 150 250 500 750 1000 1250
DOSE (mg)
ETA4 (KA)
1.5
1.0
0.5
0.0
-0.5
-1.0
-1.5
50 150 250 500 750 1000 1250
DOSE (mg)
ETA5 (MTT)
2
1
0
-1
50 150 250 500 750 1000 1250
DOSE (mg)
ETA6 (F1)
0.3
0.2
0.1
0.0
-0.1
-0.2
50 150 250 500 750 1000 1250
DOSE (mg)

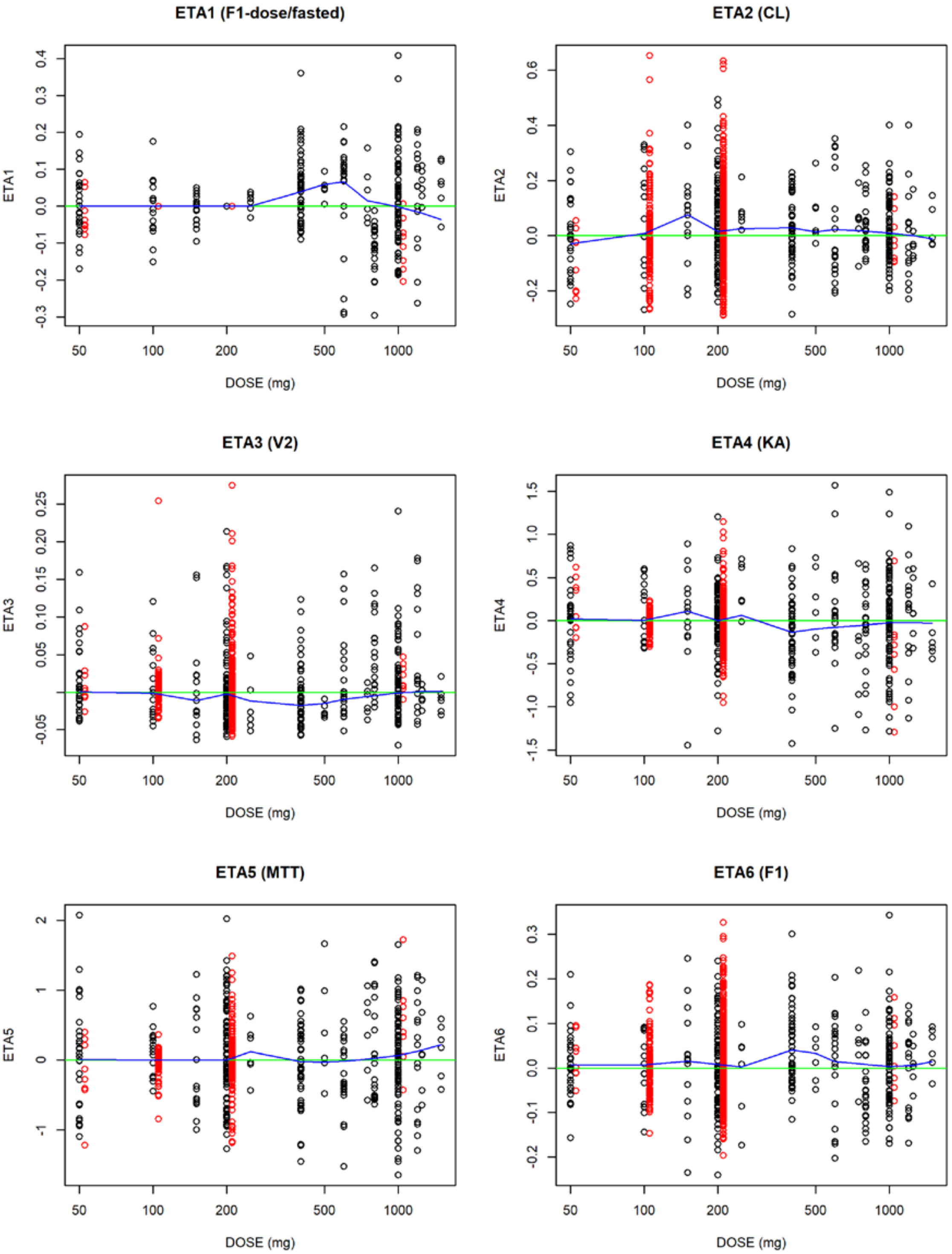
ETA1 (F1-dose/fasted)
ETA1
-0.3 -0.2 -0.1 0.0 0.1 0.2 0.3 0.4
50 100 200 500 1000
DOSE (mg)
ETA2 (CL)
ETA2
-0.2 0.0 0.2 0.4 0.6
50 100 200 500 1000
DOSE (mg)
ETA3 (V2)
ETA3
-0.05 0.05 0.10 0.15 0.20 0.25
50 100 200 500 1000
DOSE (mg)
ETA4 (KA)
ETA4
-1.5 -1.0 -0.5 0.0 0.5 1.0 1.5
50 100 200 500 1000
DOSE (mg)
ETA5 (MTT)
ETA5
-1 0 1 2
50 100 200 500 1000
DOSE (mg)
ETA6 (F1)
ETA6
-0.2 -0.1 0.0 0.1 0.2 0.3
50 100 200 500 1000
DOSE (mg)

Figure S5 Prediction-based diagnostics, set 2

CWRES and $\hat{\eta}$'s vs Population and weight

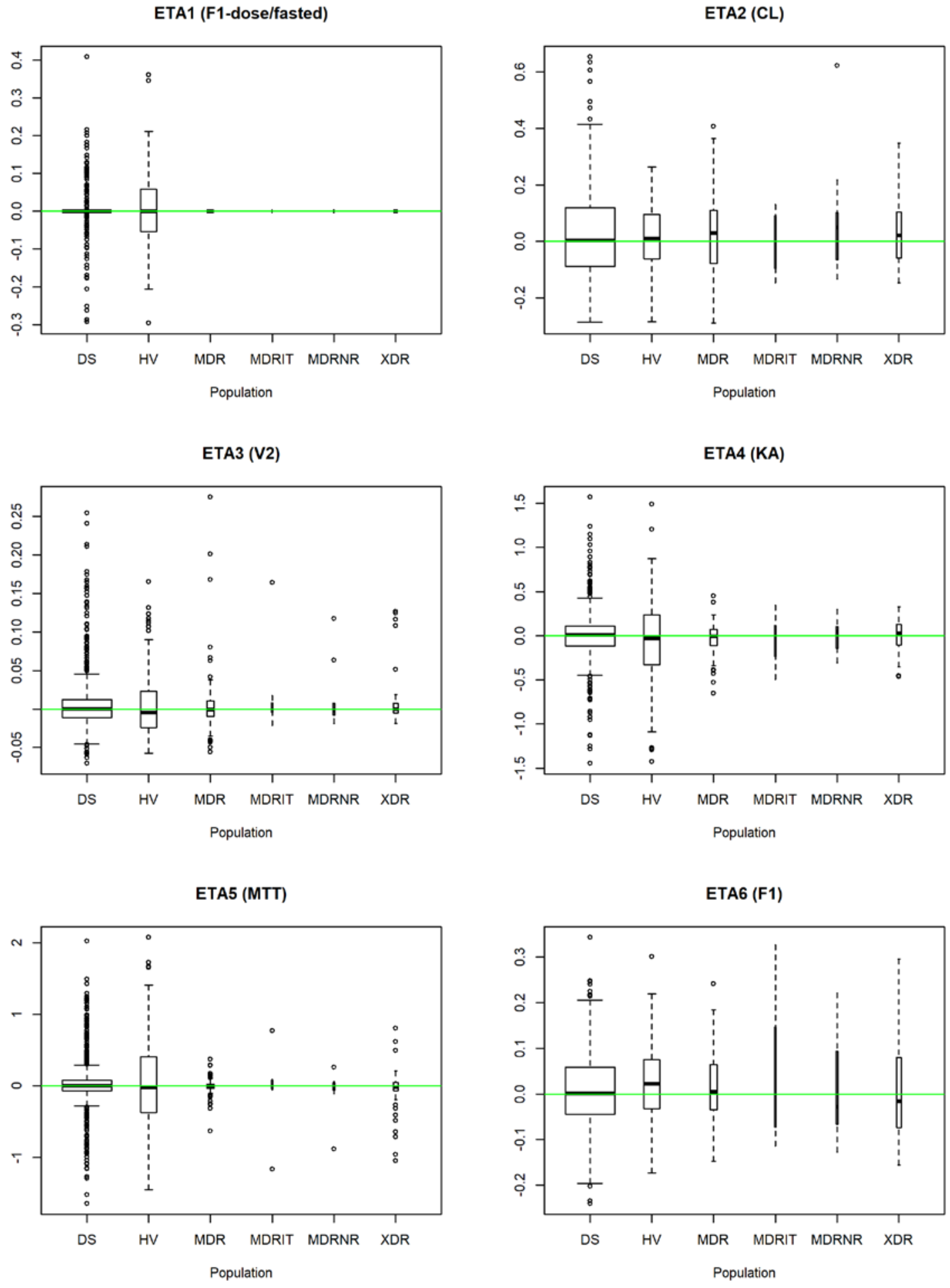

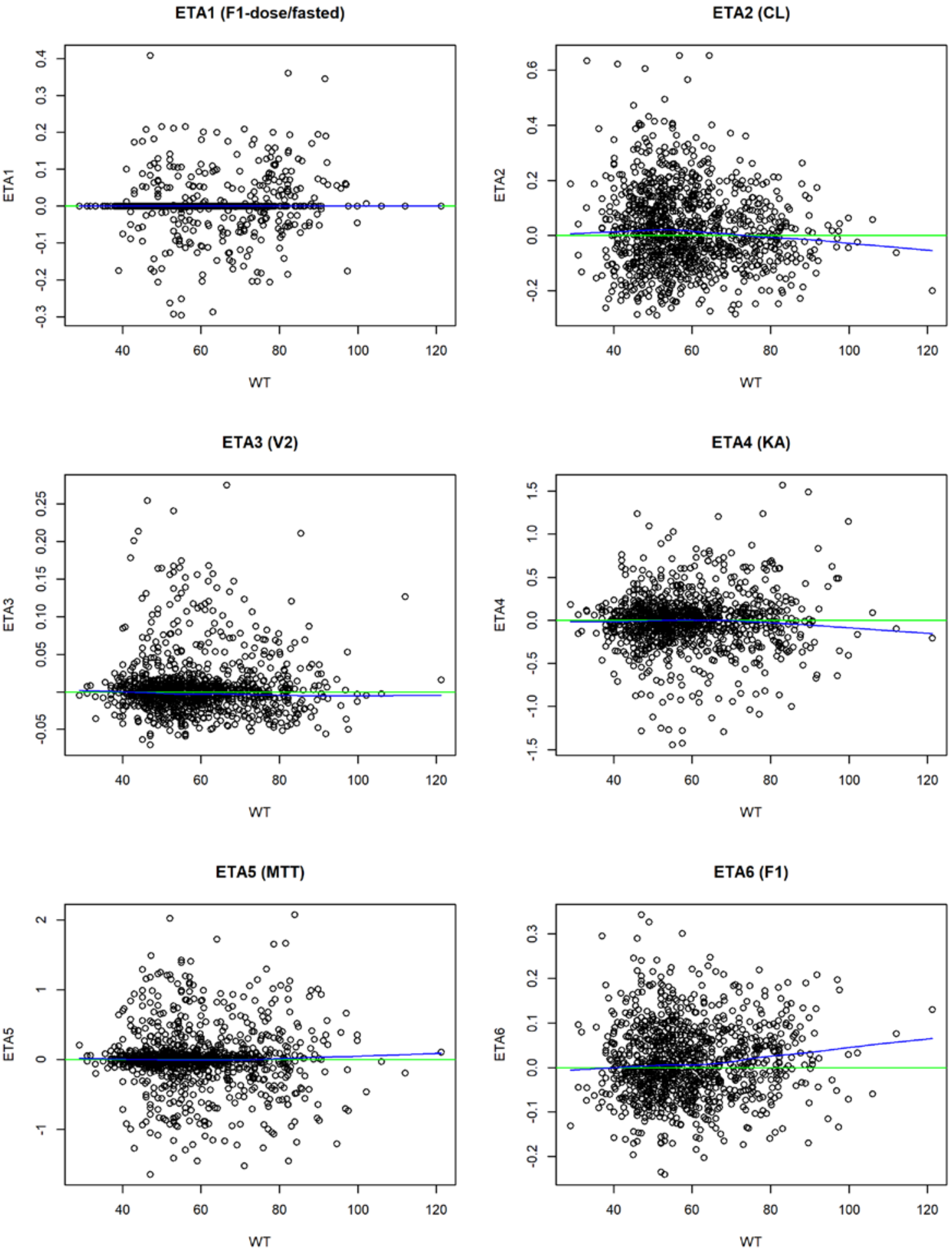
ETA1 (F1-dose/fasted)
ETA2 (CL)
ETA3 (V2)
ETA4 (KA)
ETA5 (MTT)
ETA6 (F1)
ETA1
ETA2
ETA3
ETA4
ETA5
ETA6
WT

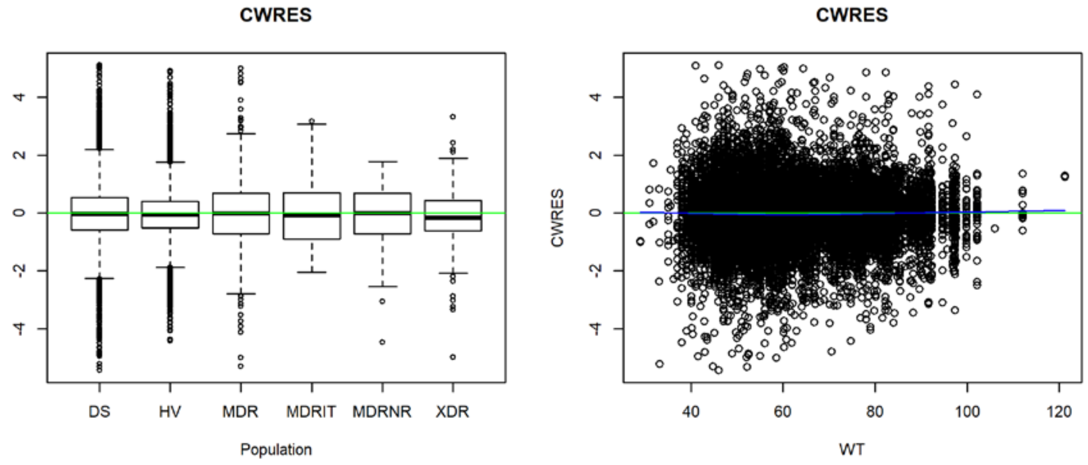
CWRES
DS
HV
MDR
MDRIT
MDRNR
XDR
Population
CWRES
CWRES
WT
4
2
0
-2
-4
40
60
80
100
120

Figure S6 Prediction-based diagnostics, set 3

$\hat{\eta}$'s and CWRES vs FED/FASTED and Regimen

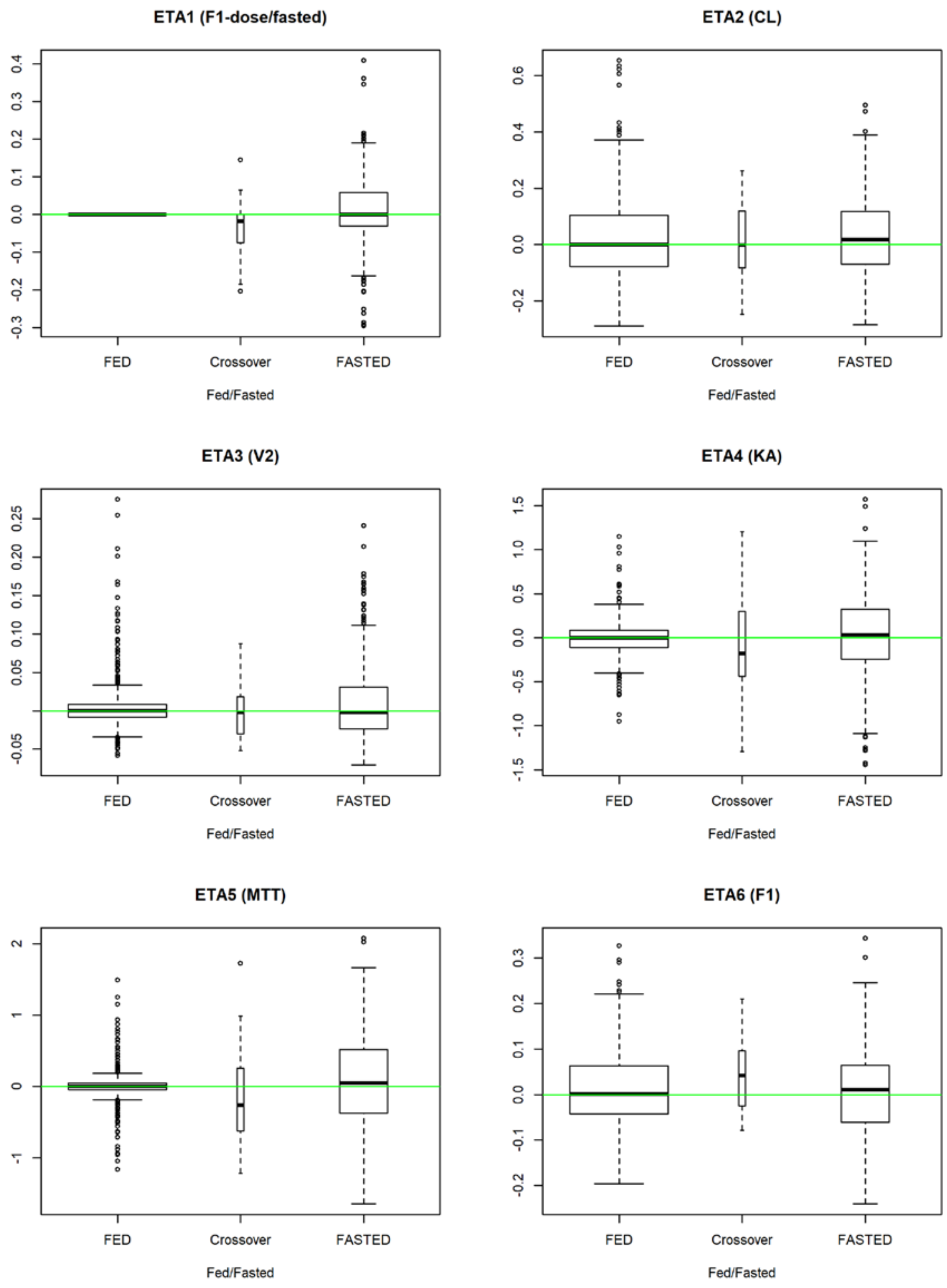

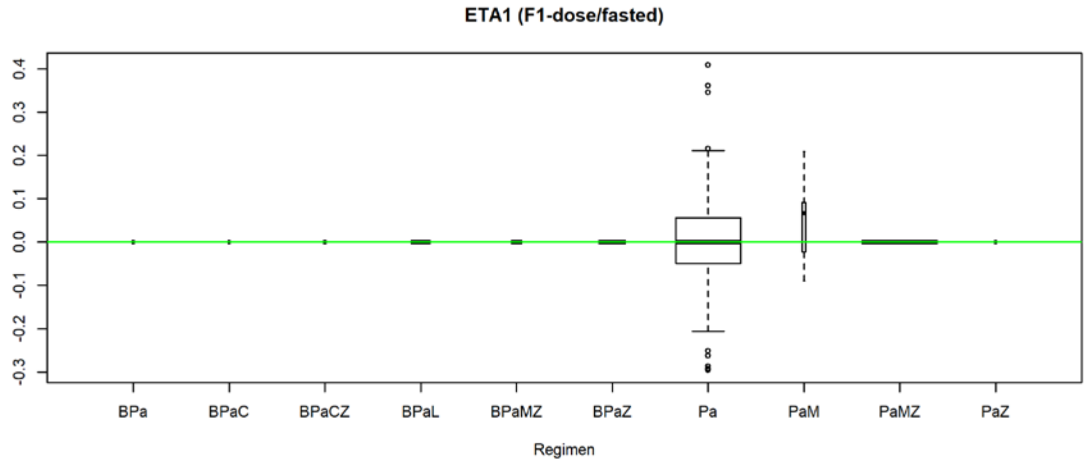
ETA1 (F1-dose/fasted)
0.4
0.3
0.2
0.1
0.0
-0.1
-0.2
-0.3
BPa
BPaC
BPaCZ
BPaL
BPaMZ
BPaZ
Pa
PaM
PaMZ
PaZ
Regimen

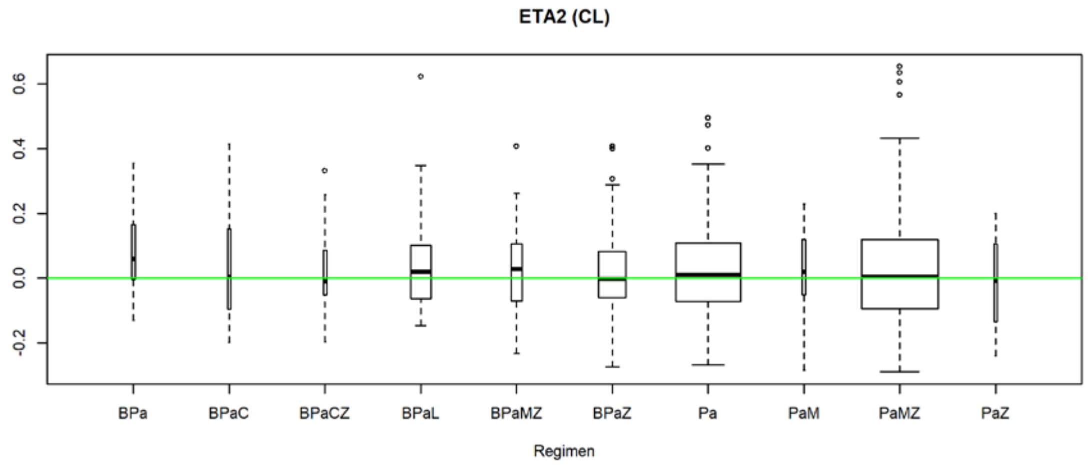
ETA2 (CL)
0.6
0.4
0.2
0.0
-0.2
BPa
BPaC
BPaCZ
BPaL
BPaMZ
BPaZ
Pa
PaM
PaMZ
PaZ
Regimen

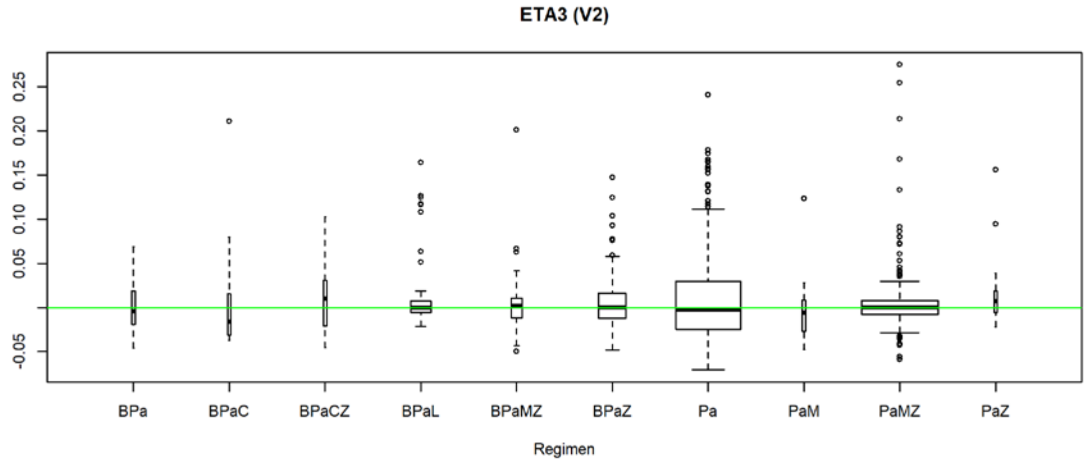
ETA3 (V2)
0.25
0.20
0.15
0.10
0.05
-0.05
BPa
BPaC
BPaCZ
BPaL
BPaMZ
BPaZ
Pa
PaM
PaMZ
PaZ
Regimen

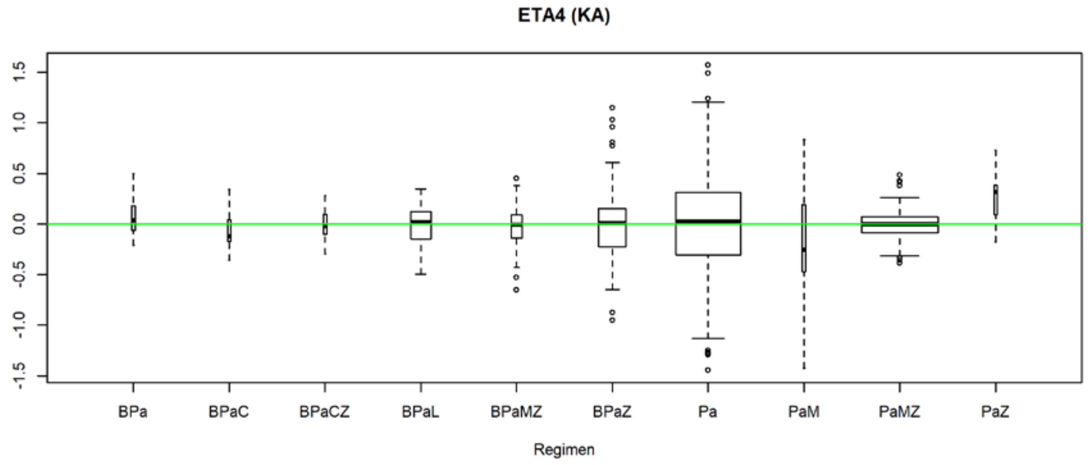
ETA4 (KA)
1.5
1.0
0.5
0.0
-0.5
-1.0
-1.5
BPa
BPaC
BPaCZ
BPaL
BPaMZ
BPaZ
Pa
PaM
PaMZ
PaZ
Regimen

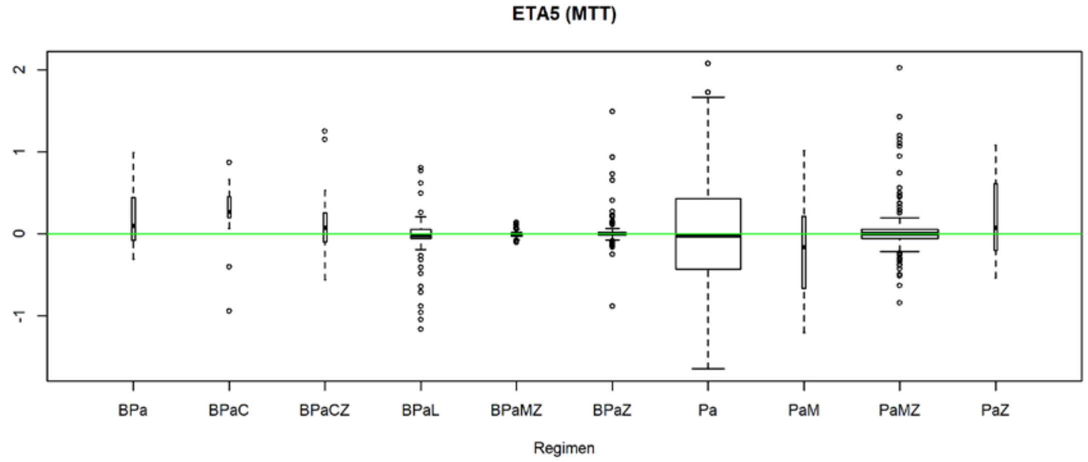
ETA5 (MTT)
2
1
0
-1
BPa
BPaC
BPaCZ
BPaL
BPaMZ
BPaZ
Pa
PaM
PaMZ
PaZ
Regimen

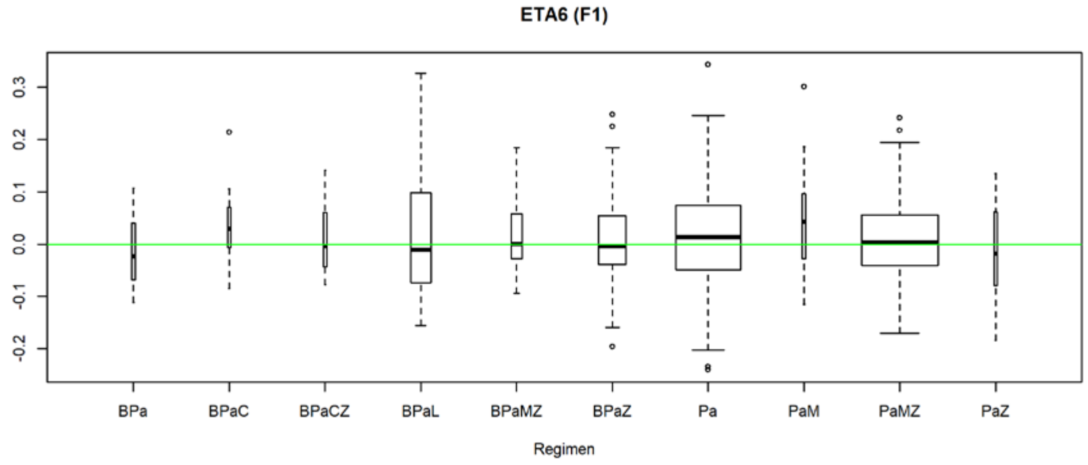
ETA6 (F1)
0.3
0.2
0.1
0.0
-0.1
-0.2
BPa
BPaC
BPaCZ
BPaL
BPaMZ
BPaZ
Pa
PaM
PaMZ
PaZ
Regimen

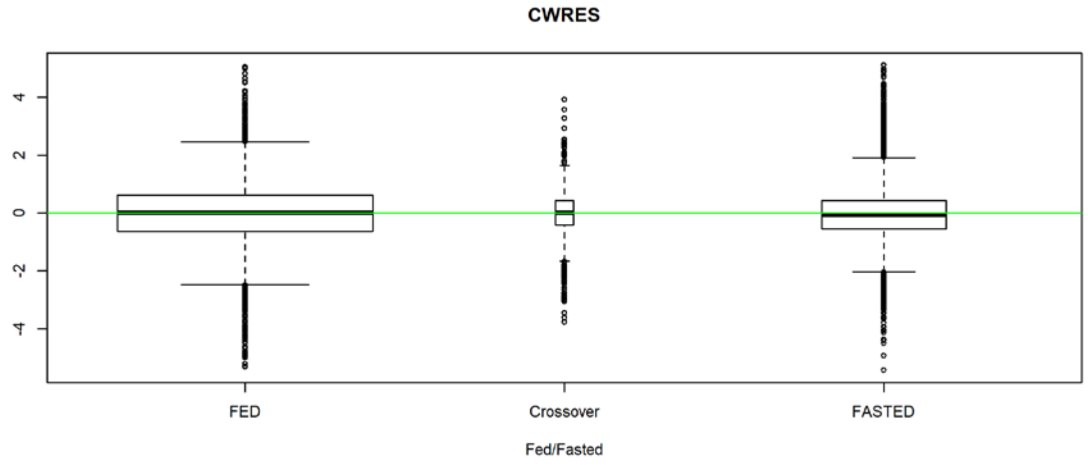
CWRES
4
2
0
-2
-4
FED
Crossover
FASTED
Fed/Fasted

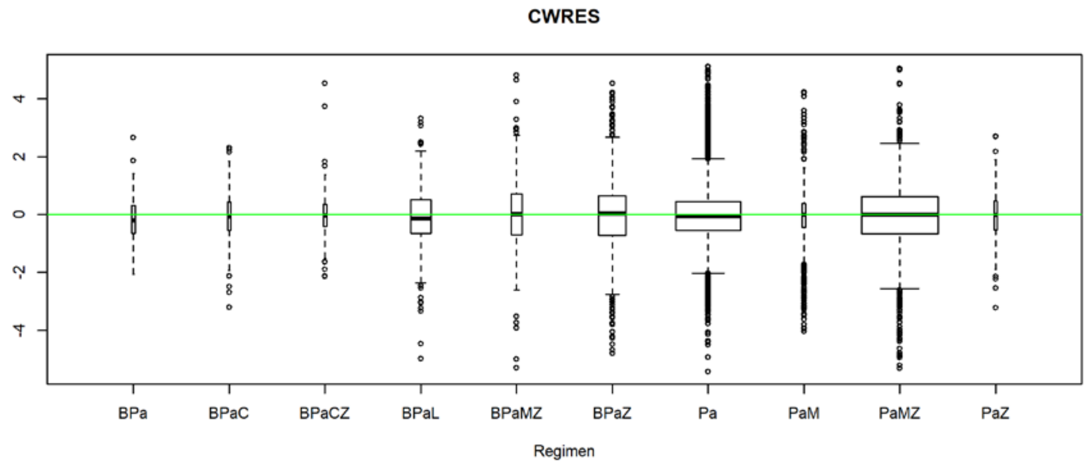
CWRES
4
2
0
-2
-4
BPa
BPaC
BPaCZ
BPaL
BPaMZ
BPaZ
Pa
PaM
PaMZ
PaZ
Regimen

Figure S7 Prediction-based diagnostics, set 4

$\hat{\eta}$'s and CWRES vs HIV status, age, sex, race, CRCL, EGFR, AST, ALT, TBIL, ALB

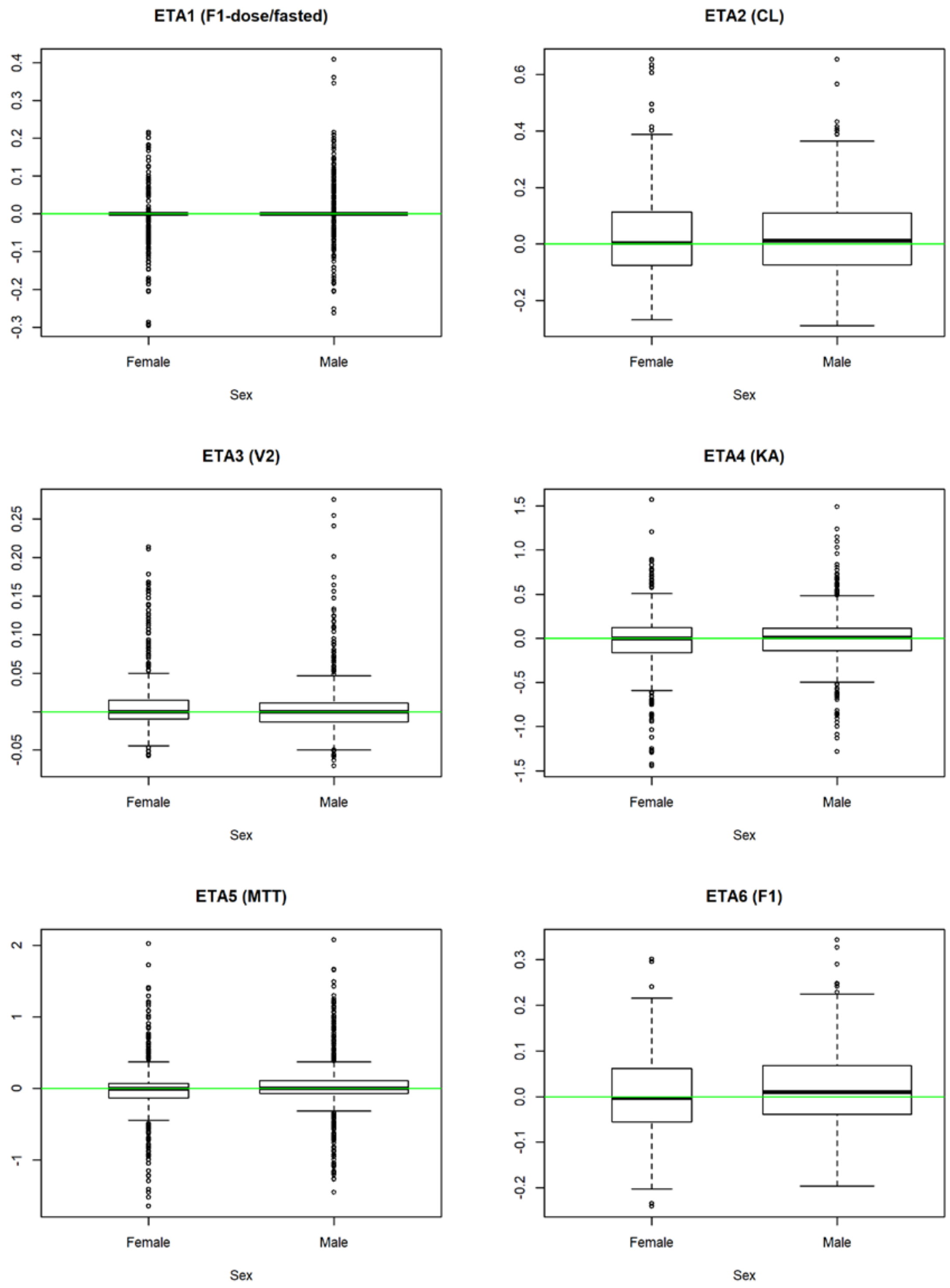

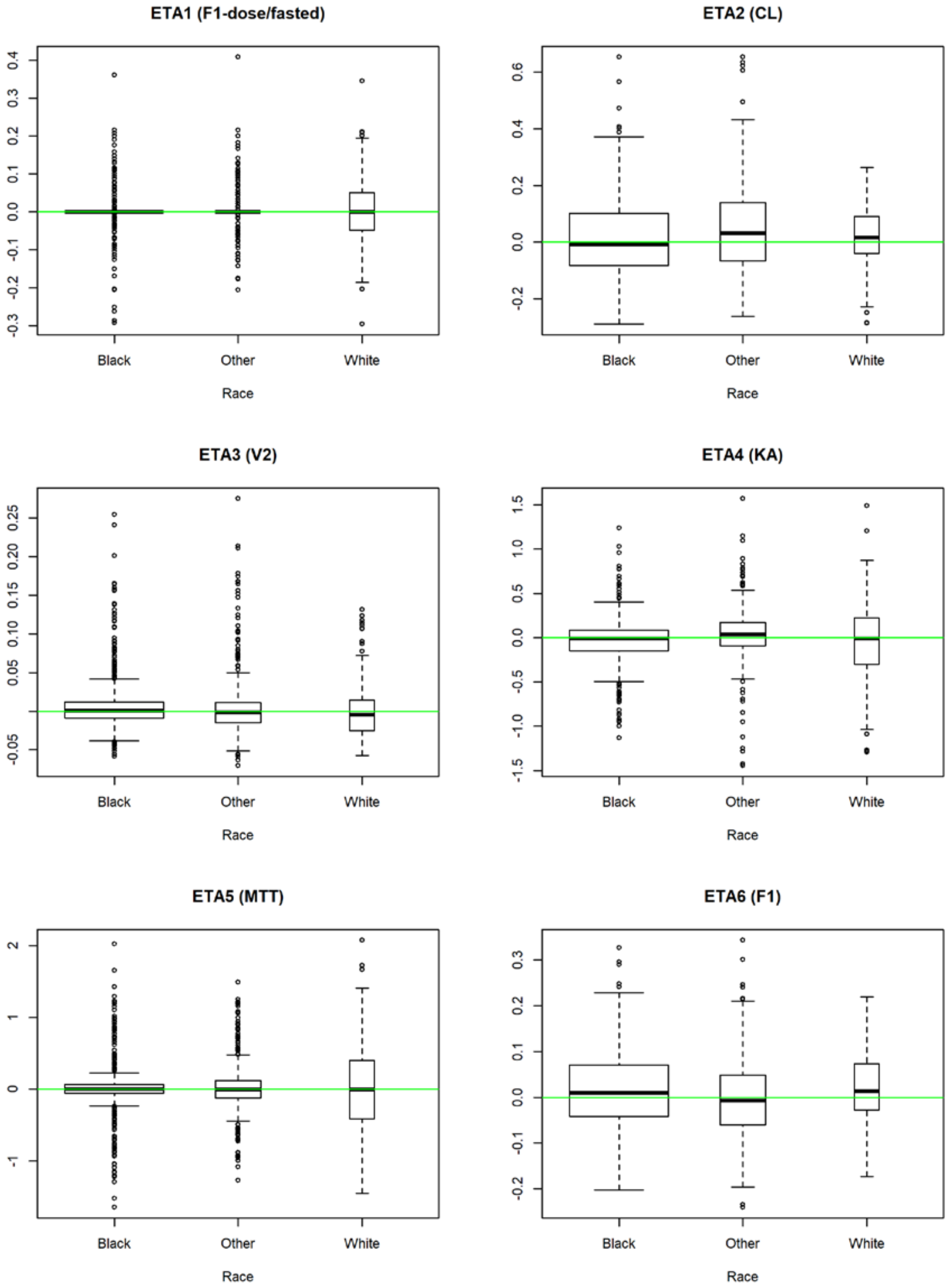
ETA1 (F1-dose/fasted)
ETA2 (CL)
ETA3 (V2)
ETA4 (KA)
ETA5 (MTT)
ETA6 (F1)
Black
Other
White
Race

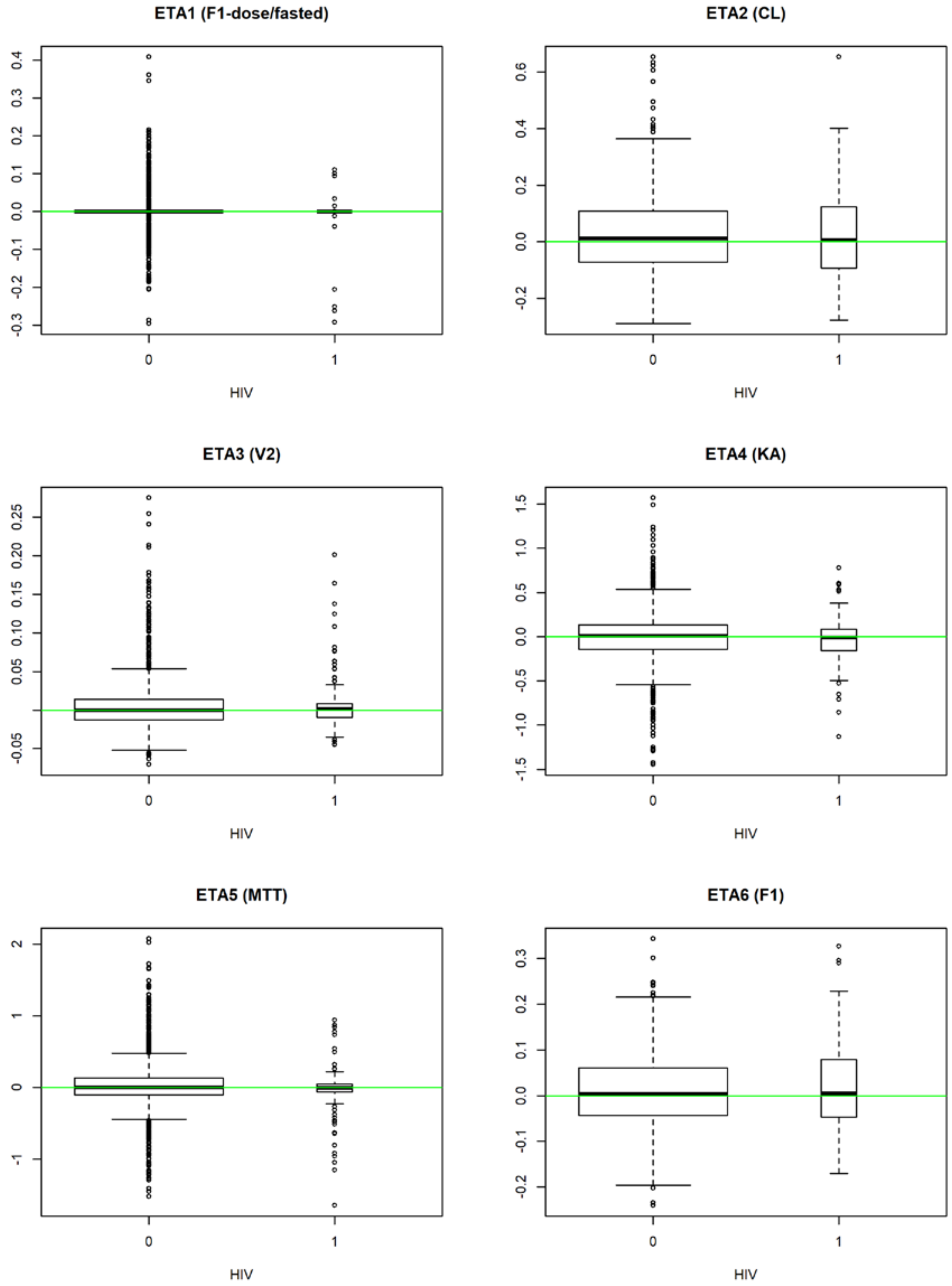
ETA1 (F1-dose/fasted)
ETA2 (CL)
ETA3 (V2)
ETA4 (KA)
ETA5 (MTT)
ETA6 (F1)
HIV
0
1

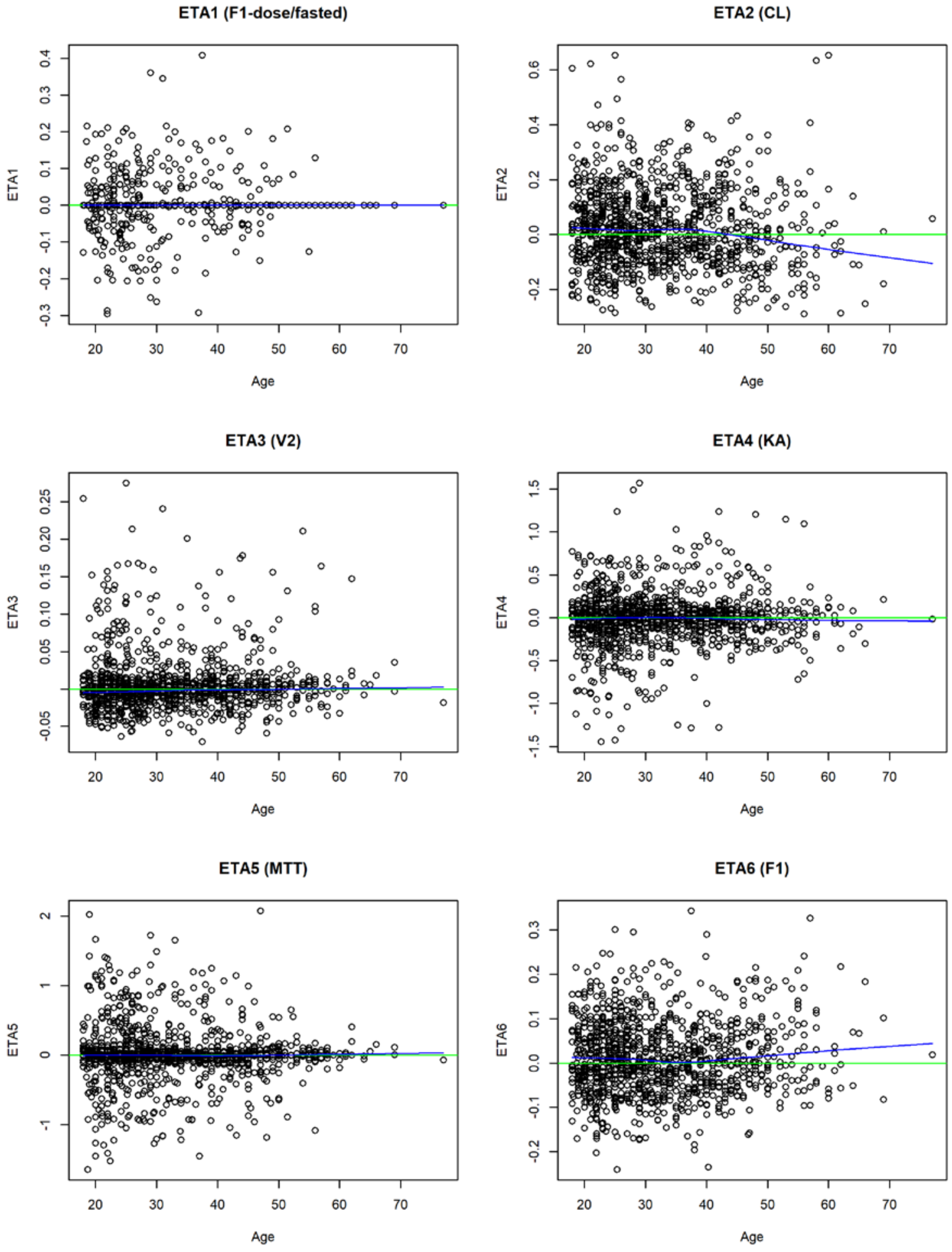
ETA1 (F1-dose/fasted)
ETA2 (CL)
ETA3 (V2)
ETA4 (KA)
ETA5 (MTT)
ETA6 (F1)
ETA1
ETA2
ETA3
ETA4
ETA5
ETA6
Age

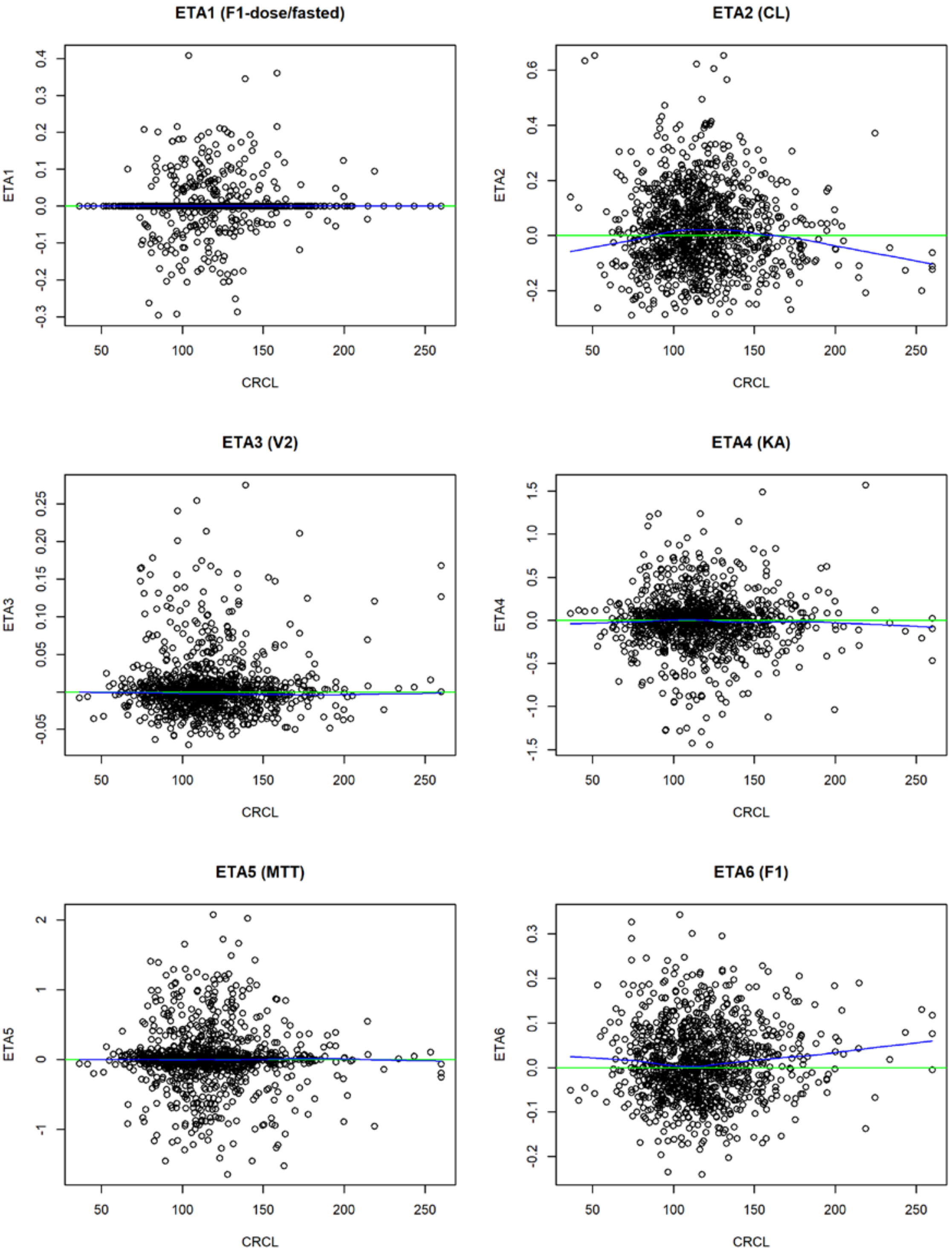
ETA1 (F1-dose/fasted)
ETA1
CRCL
ETA2 (CL)
ETA2
CRCL
ETA3 (V2)
ETA3
CRCL
ETA4 (KA)
ETA4
CRCL
ETA5 (MTT)
ETA5
CRCL
ETA6 (F1)
ETA6
CRCL

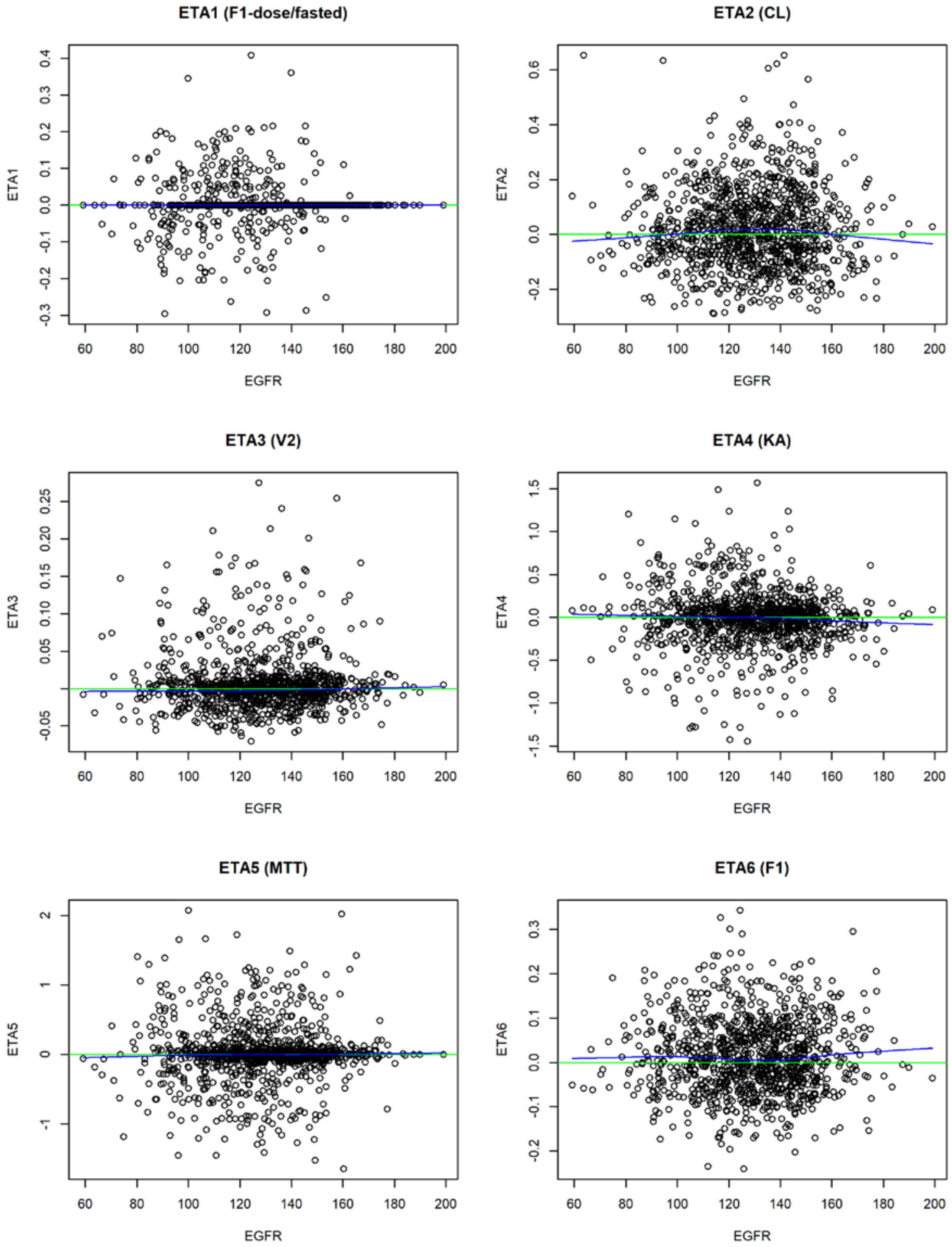
ETA1 (F1-dose/fasted)
ETA1
EGFR
ETA2 (CL)
ETA2
EGFR
ETA3 (V2)
ETA3
EGFR
ETA4 (KA)
ETA4
EGFR
ETA5 (MTT)
ETA5
EGFR
ETA6 (F1)
ETA6
EGFR

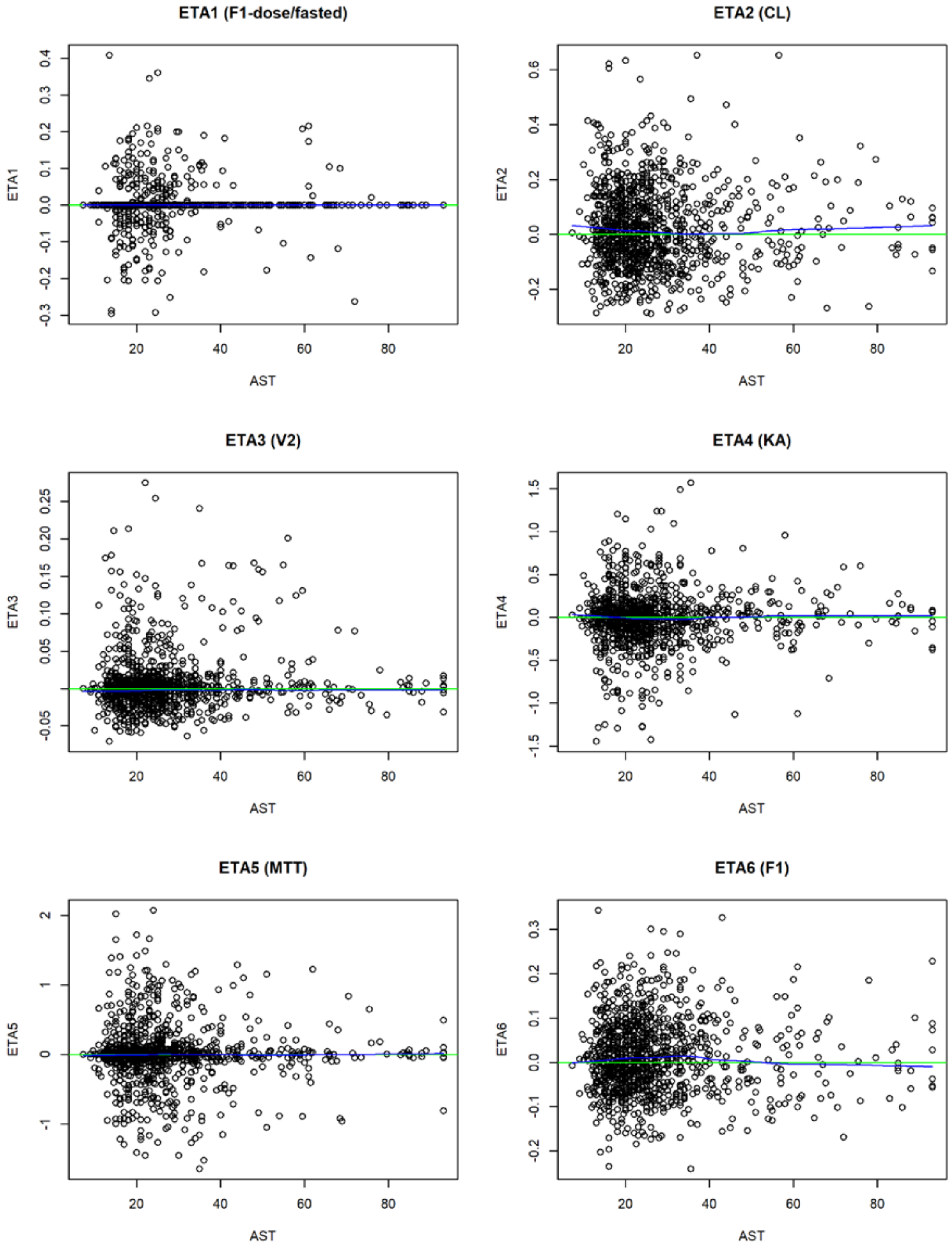
ETA1 (F1-dose/fasted)
ETA2 (CL)
ETA3 (V2)
ETA4 (KA)
ETA5 (MTT)
ETA6 (F1)
ETA1
ETA2
ETA3
ETA4
ETA5
ETA6
AST

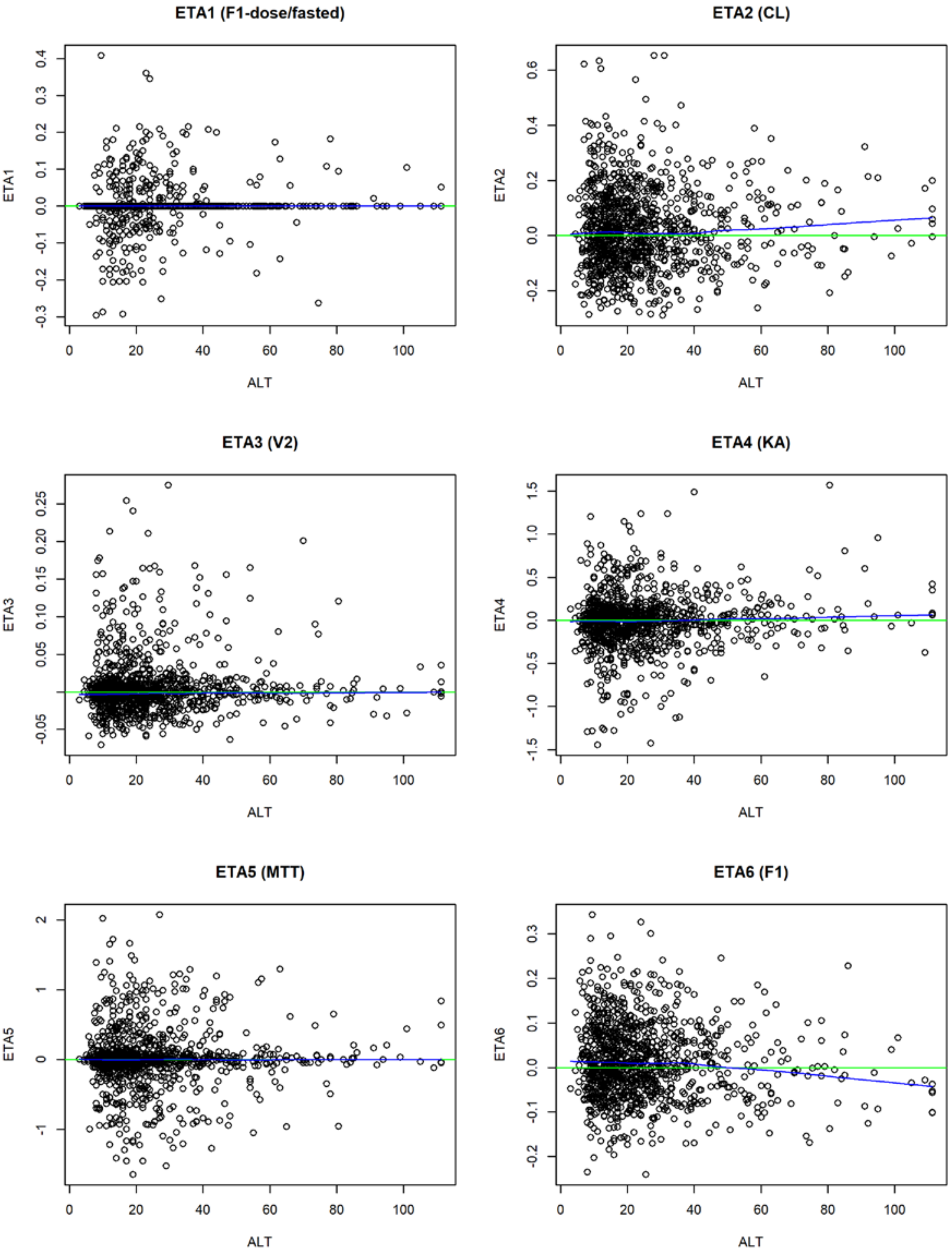
ETA1 (F1-dose/fasted)
ETA1
ALT
ETA2 (CL)
ETA2
ALT
ETA3 (V2)
ETA3
ALT
ETA4 (KA)
ETA4
ALT
ETA5 (MTT)
ETA5
ALT
ETA6 (F1)
ETA6
ALT

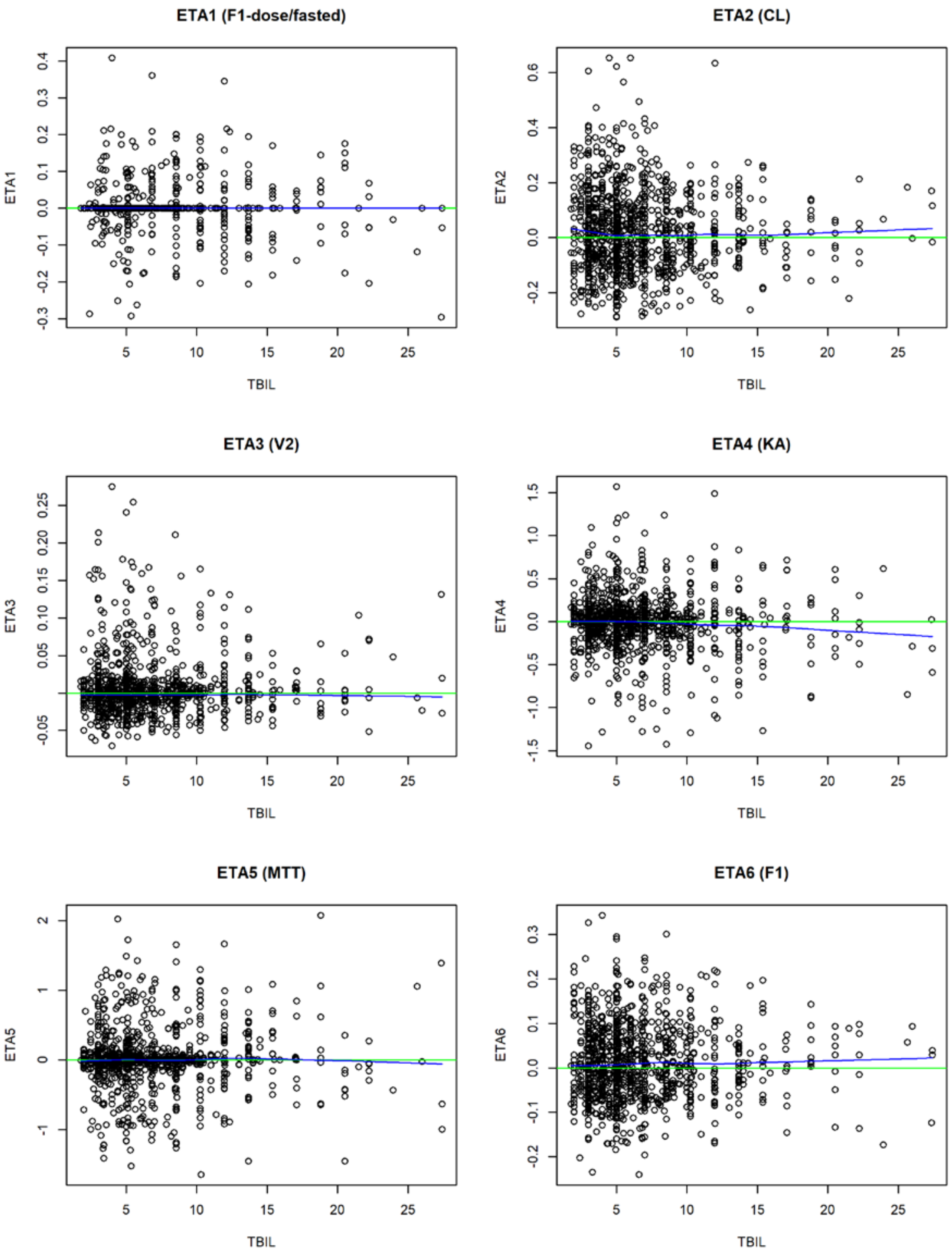
ETA1 (F1-dose/fasted)
ETA1
TBIL
ETA2 (CL)
ETA2
TBIL
ETA3 (V2)
ETA3
TBIL
ETA4 (KA)
ETA4
TBIL
ETA5 (MTT)
ETA5
TBIL
ETA6 (F1)
ETA6
TBIL

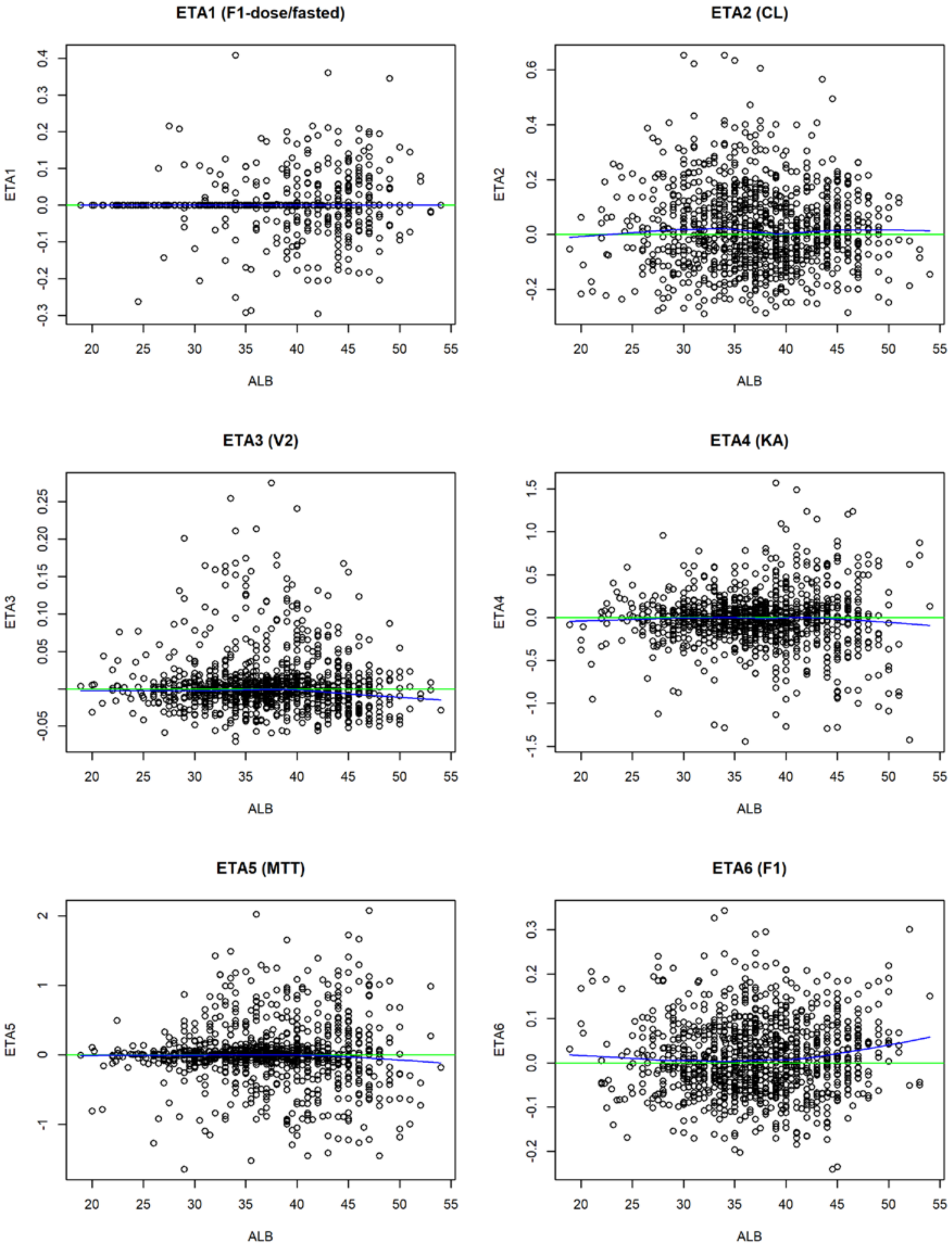
ETA1 (F1-dose/fasted)
ETA2 (CL)
ETA3 (V2)
ETA4 (KA)
ETA5 (MTT)
ETA6 (F1)
ETA1
ETA2
ETA3
ETA4
ETA5
ETA6
ALB

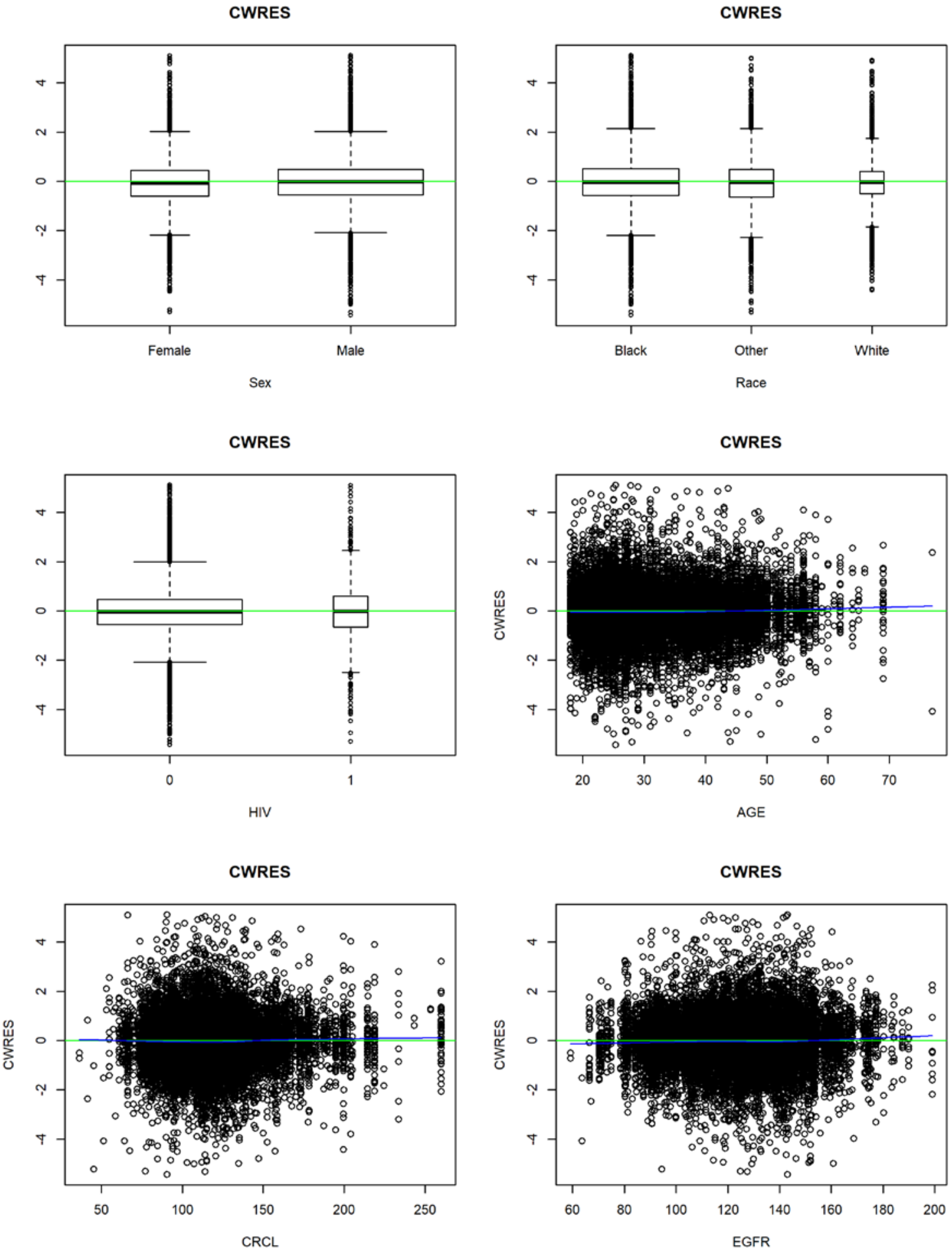
CWRES
Female
Male
Sex
CWRES
Black
Other
White
Race
CWRES
0
1
HIV
CWRES
CWRES
AGE
CWRES
CWRES
CRCL
CWRES
CWRES
EGFR

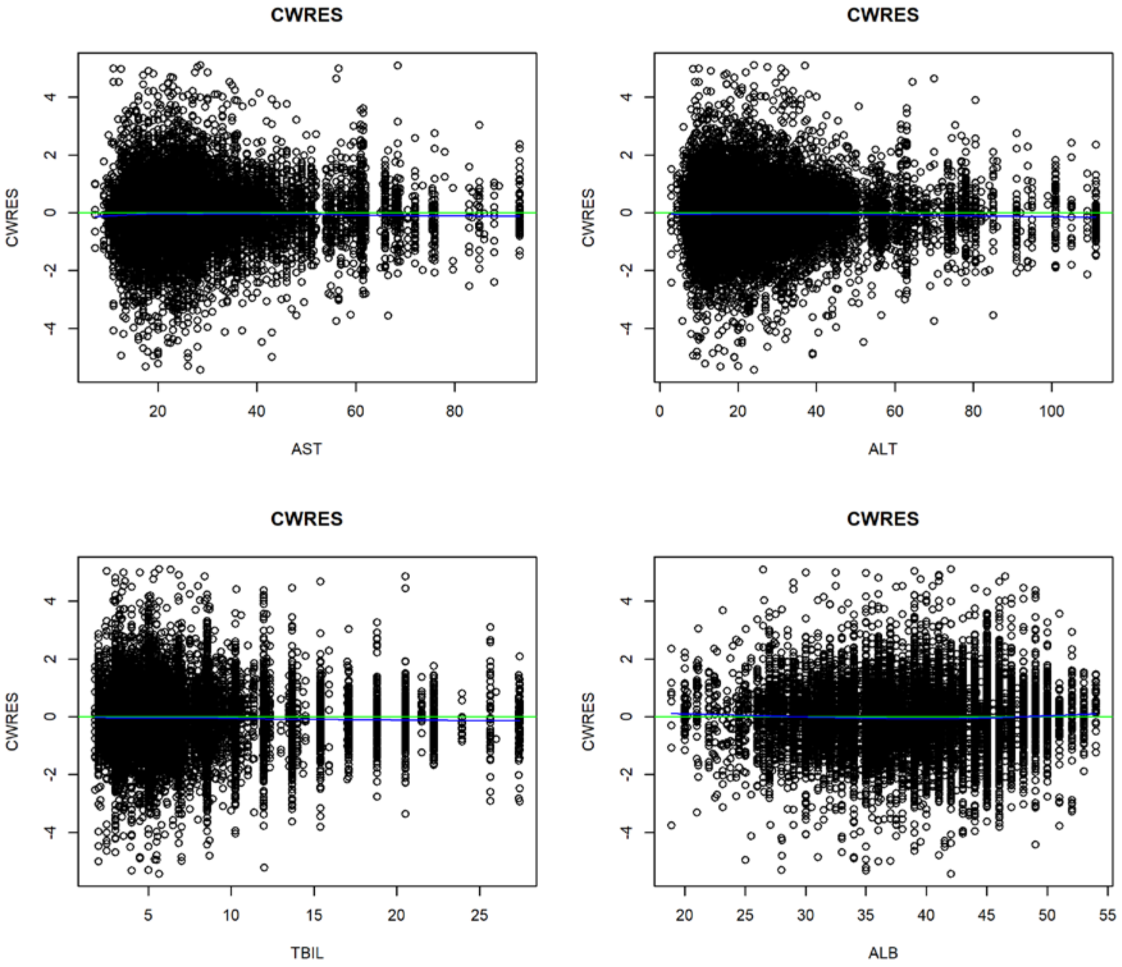
CWRES
CWRES
AST
CWRES
CWRES
ALT
CWRES
CWRES
TBIL
CWRES
CWRES
ALB

Figure S8 Prediction-based diagnostics, set 5

Histograms, densities, and normal probability plots of $\hat{\eta}$'s; scatterplot matrix of $\hat{\eta}$'s

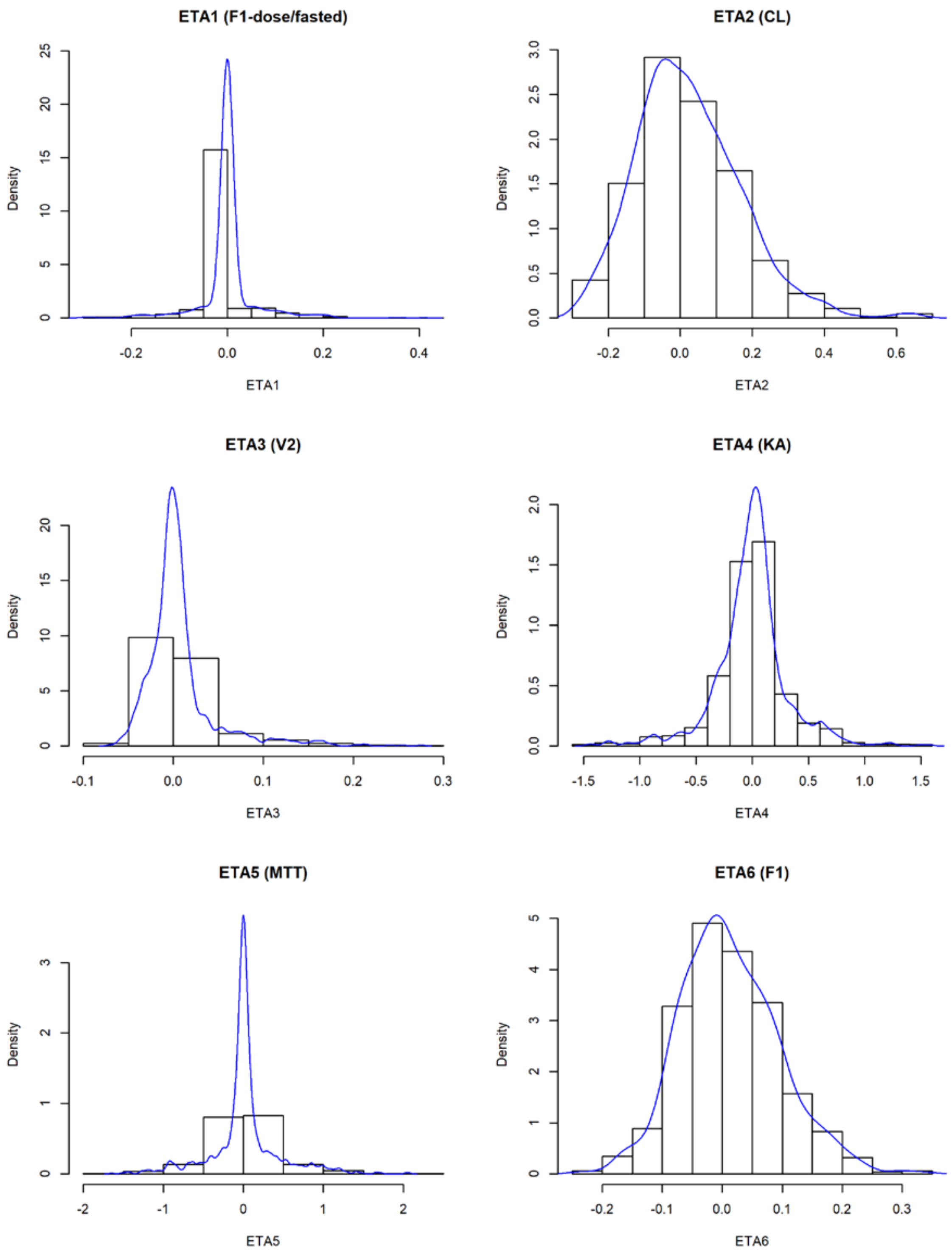

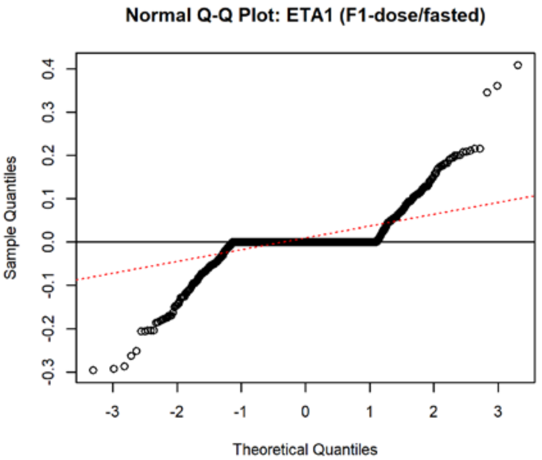
Normal Q-Q Plot: ETA1 (F1-dose/fasted)
Sample Quantiles
Theoretical Quantiles

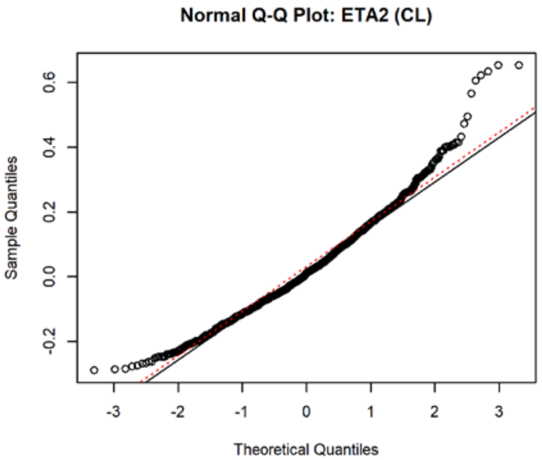
Normal Q-Q Plot: ETA2 (CL)
Sample Quantiles
Theoretical Quantiles

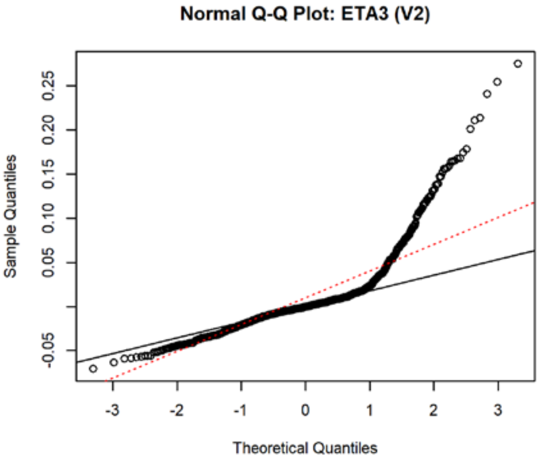
Normal Q-Q Plot: ETA3 (V2)
Sample Quantiles
Theoretical Quantiles

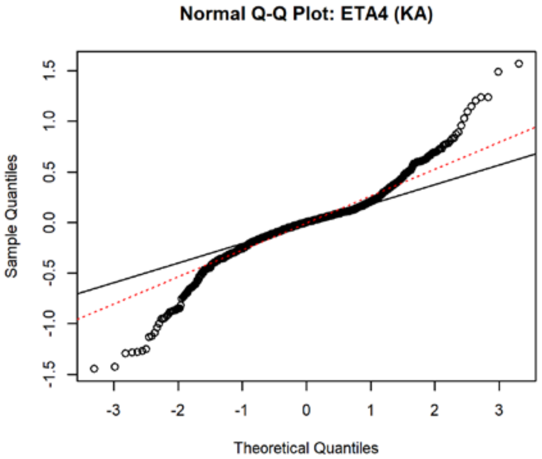
Normal Q-Q Plot: ETA4 (KA)
Sample Quantiles
Theoretical Quantiles

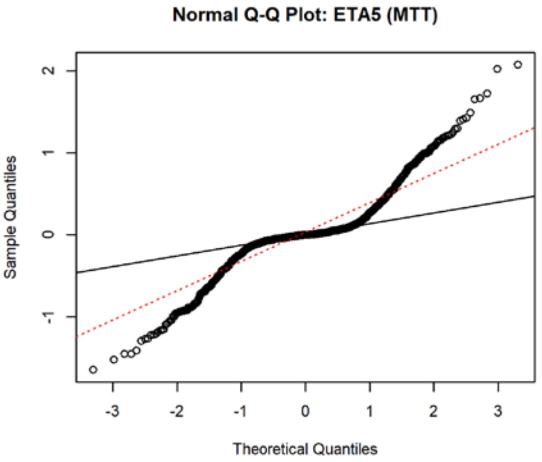
Normal Q-Q Plot: ETA5 (MTT)
Sample Quantiles
Theoretical Quantiles

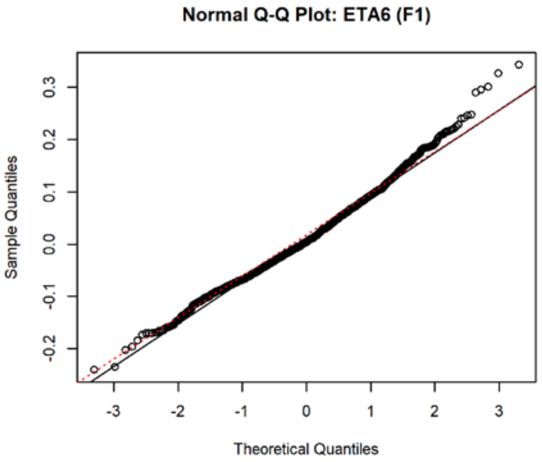
Normal Q-Q Plot: ETA6 (F1)
Sample Quantiles
Theoretical Quantiles

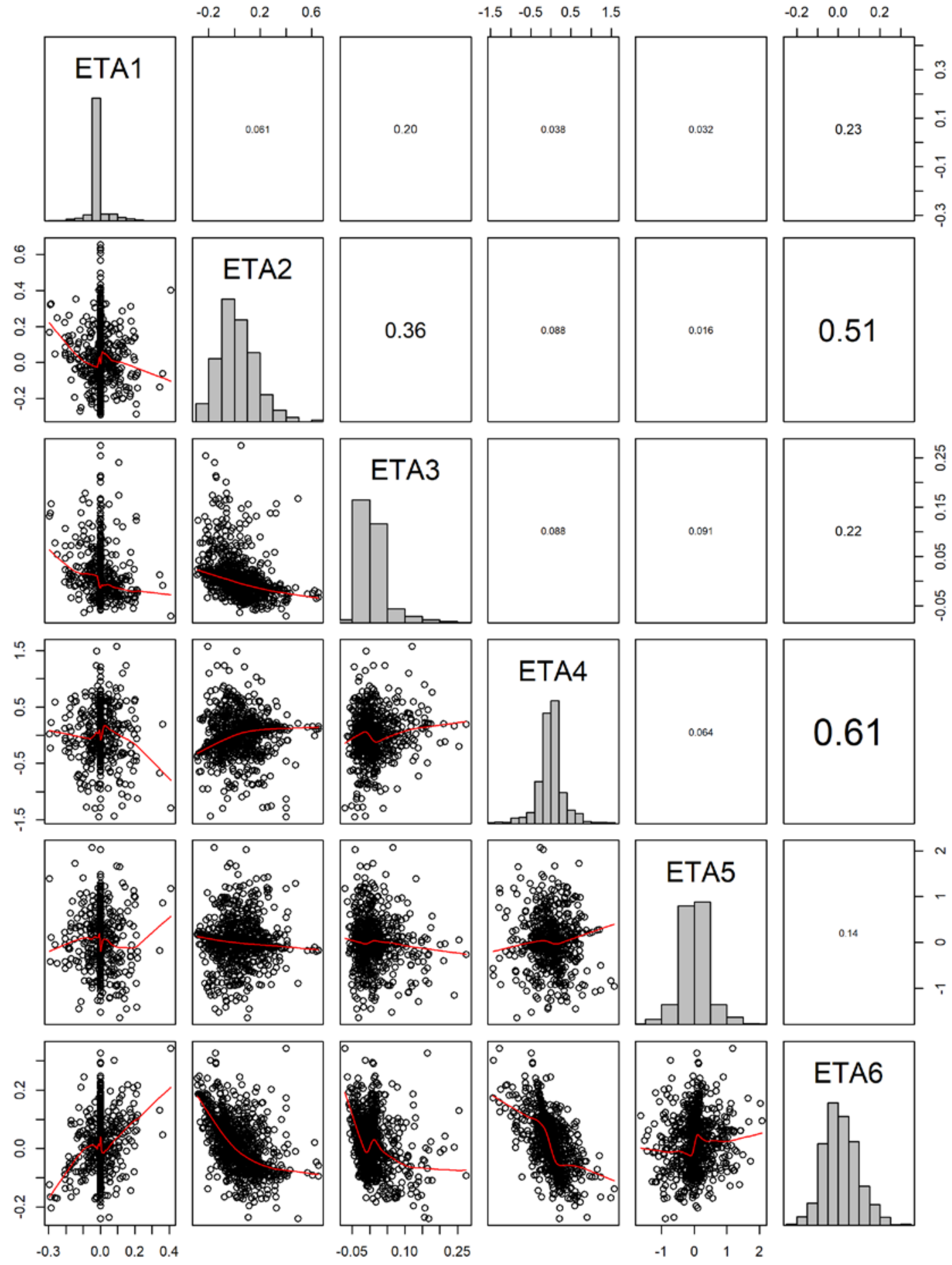

ETA1
ETA2
ETA3
ETA4
ETA5
ETA6
0.061
0.20
0.038
0.032
0.23
0.36
0.088
0.016
0.51
0.088
0.091
0.22
0.064
0.61
0.14

Figure S9 Simulation-based diagnostics, set 1

pcVPC for single-dose and steady-state profiles for pretomanid alone in healthy subjects, all doses together

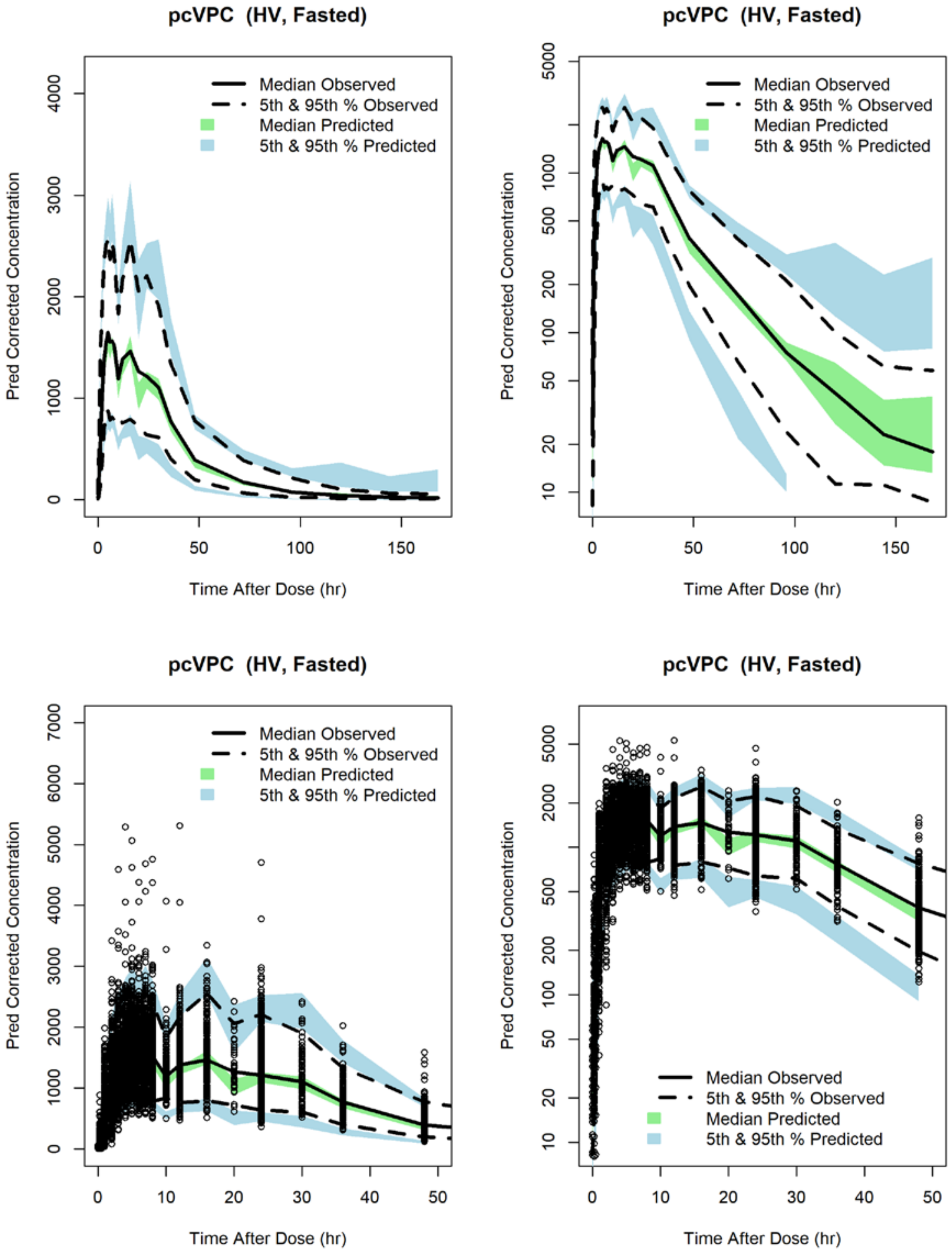

Figure S10 Simulation-based diagnostics, set 2

pcVPC for single-dose and steady-state profiles for pretomanid alone in DS patients (CL-007, CL-010), all doses together

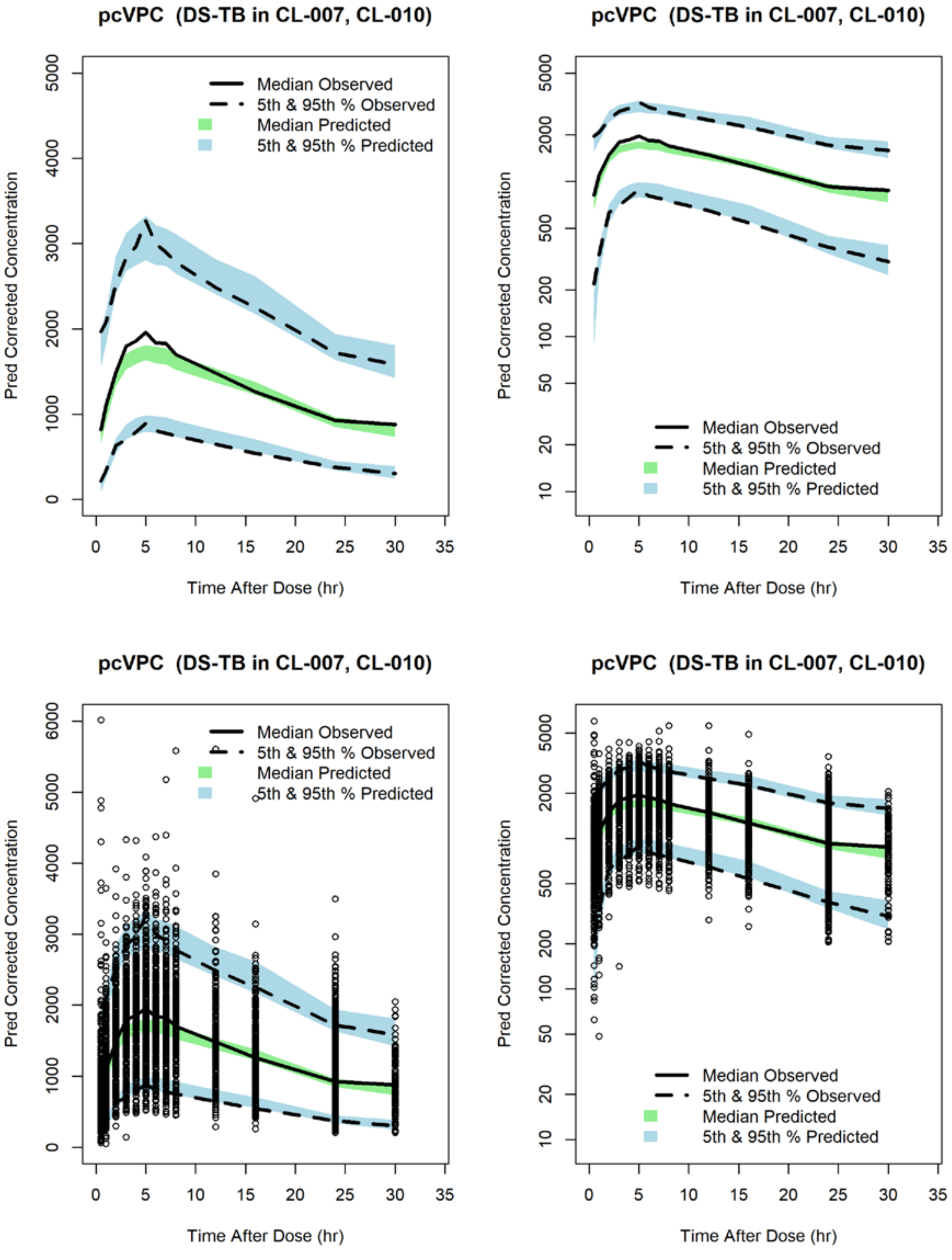

Figure S11 Simulation-based diagnostics, set 3

pcVPC for steady-state profiles for 50 – 200 mg in DS + MDR patients, all regimens together, separate plotting symbols for DS & MDR data points

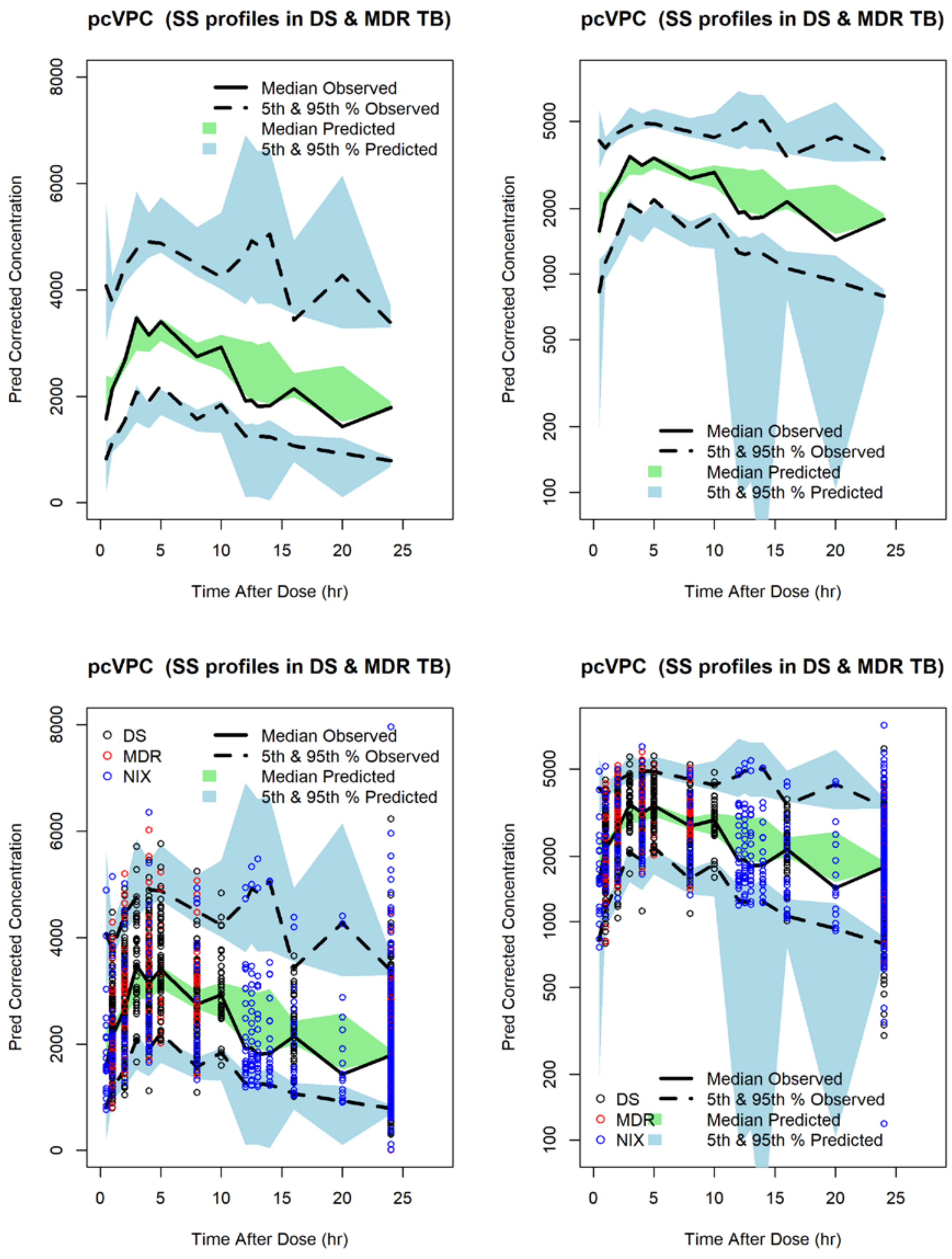

Note: Study NC-001 plotted separately

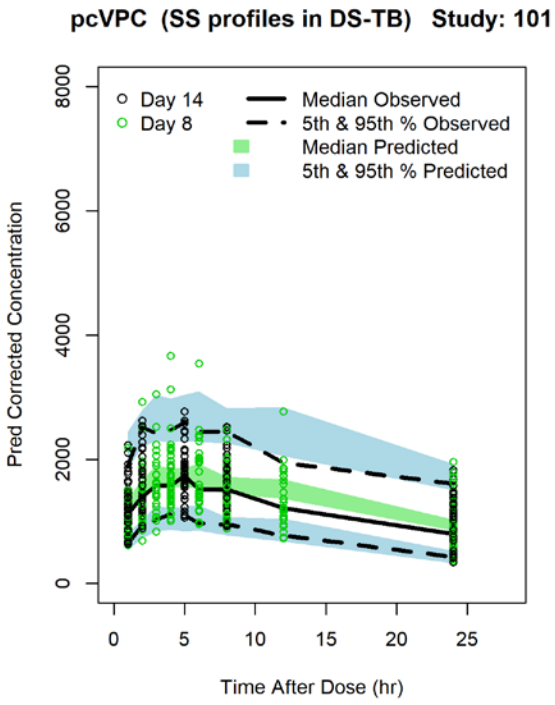

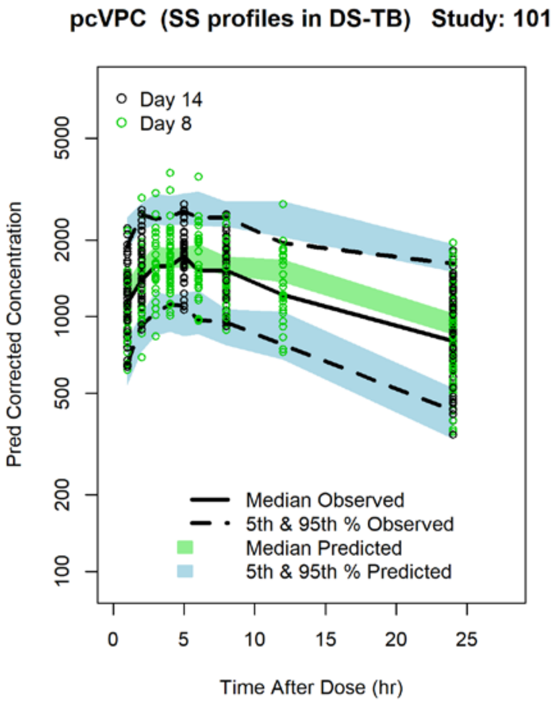

Note: Study NC-001 plotted separately

Figure S12 Simulation-based diagnostics, set 4
Nix-TB Data Only

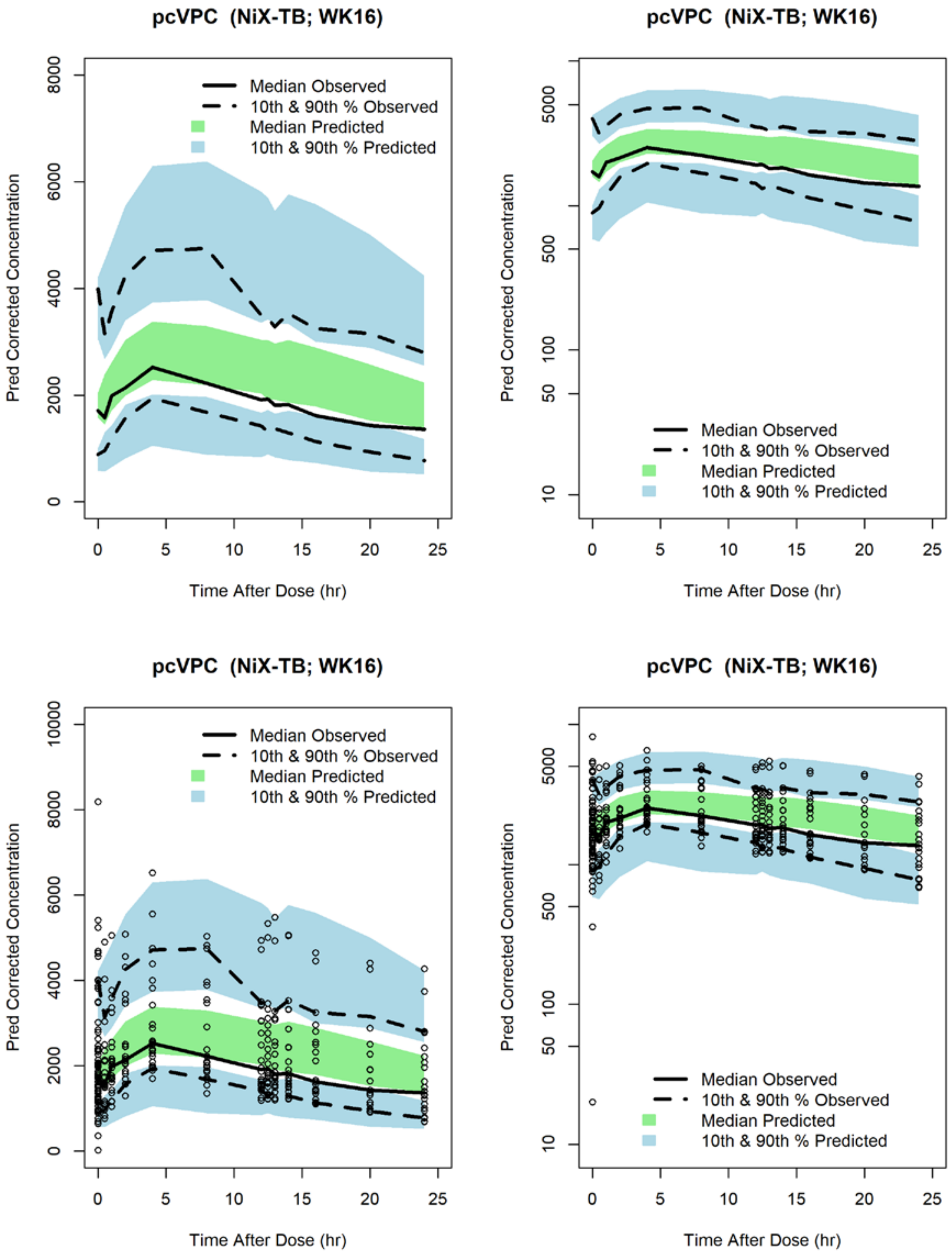

Figure S13 Simulation-based diagnostics, set 5

pcVPC for steady-state troughs for 50 – 200 mg in DS + MDR patients, all regimens together, separate plotting symbols for DS & MDR & NiX-study data points

Note: First row: through Week 8. Second row: Nix-TB data alone.
